# Supplementary material for: New evidence of Neandertal butchery traditions through the marrow extraction in southwestern Europe (MIS 5–3)
Source: PLoS One. 2022 Aug 17;17(8):e0271816. doi: 10.1371/journal.pone.0271816 (PMC9385001; doi:10.1371/journal.pone.0271816)
Supplement: S1 File — (DOCX) [file pone.0271816.s001.docx]

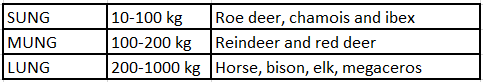


S1 Tableau: Mains size categories regarding the species involved in our analyses


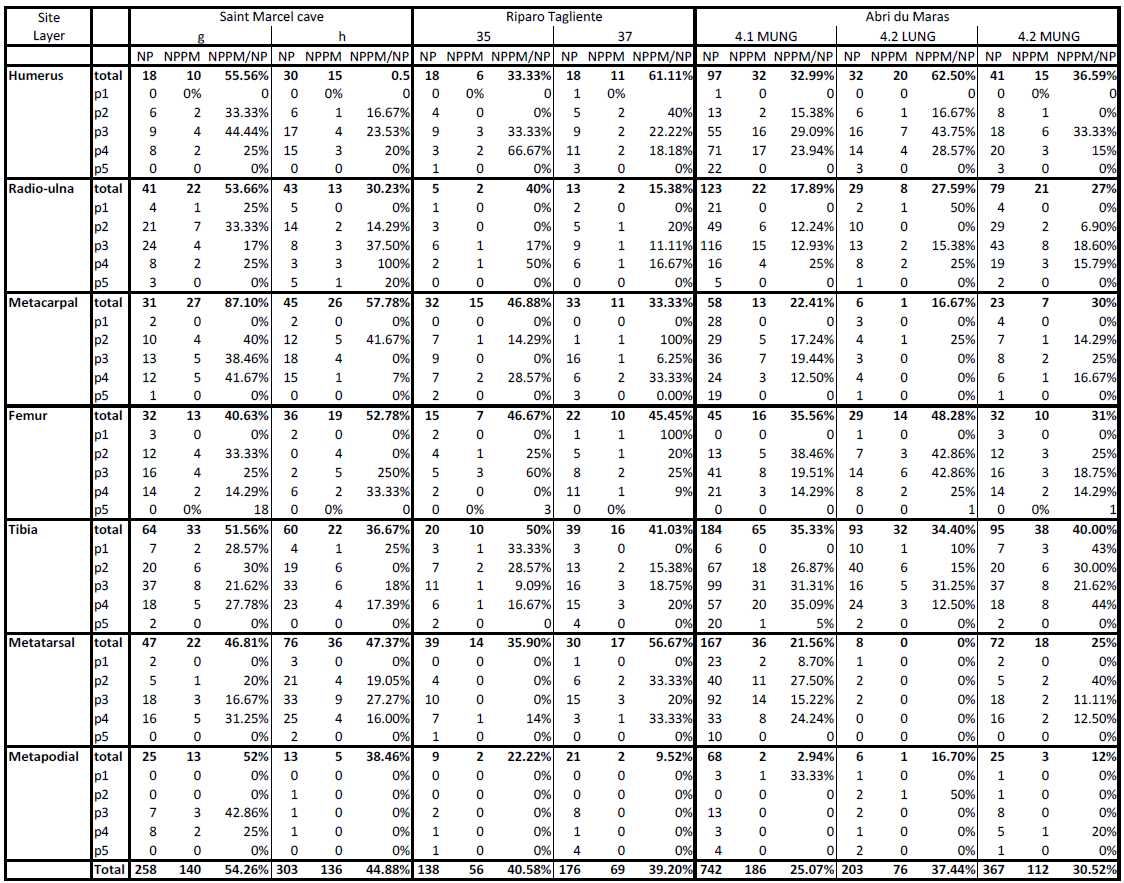
S2 Tableau: Number of portion by element (NP), number of percussion marks by portion (NPPM) by element and ratio of NP/NPM; for each site and level.


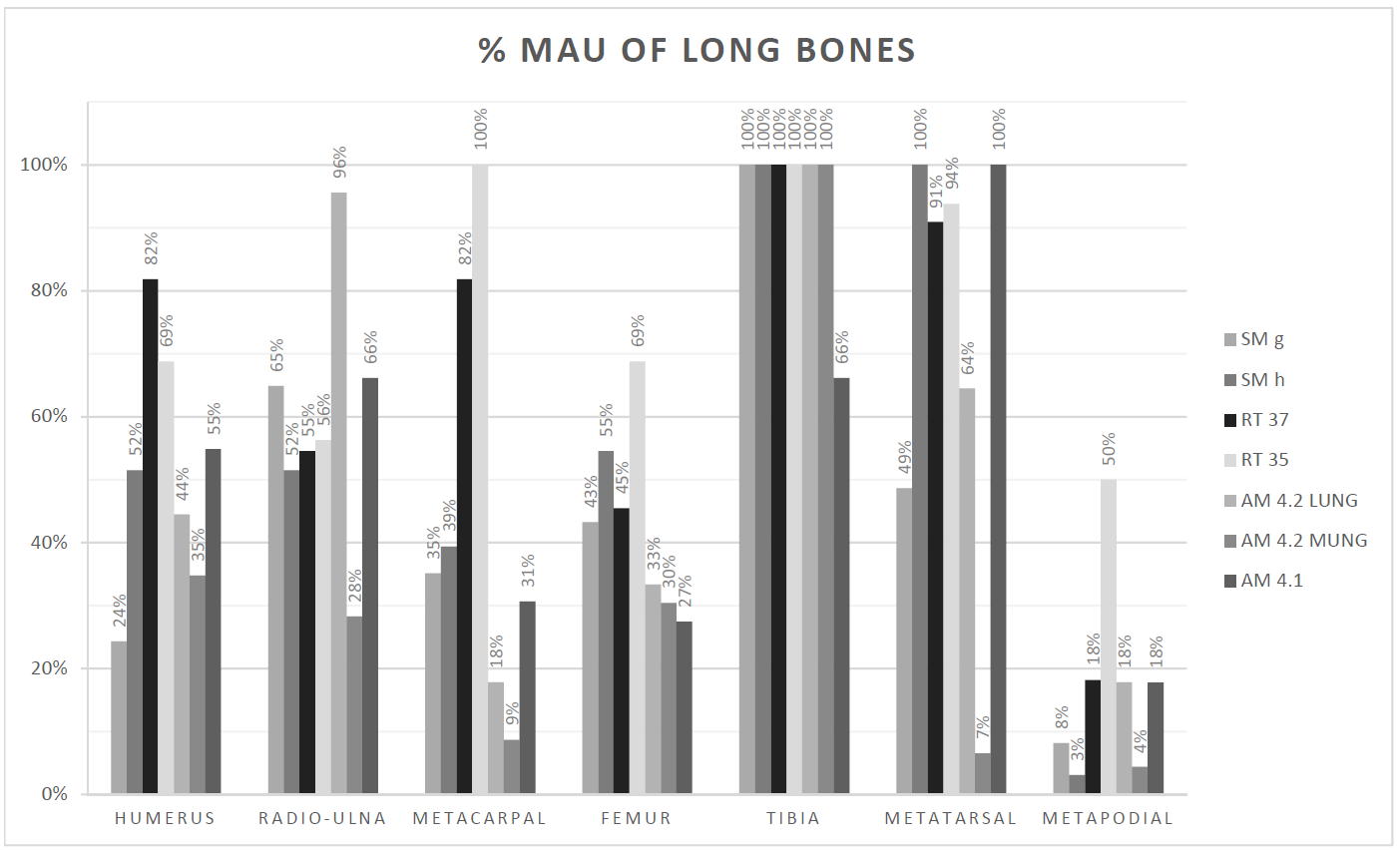


S1 Figure: % MAU regarding each element for each level of the studied sites. Saint Marcel Cave level g (SMg); Saint Marcel Cave level h (SMh); Riparo Tagliente level 35 (RT35); Riparo Tagliente level 37 (RT37); Abri du Maras level 4.1 (AM4.1); Abri du Maras level 4.2 (AM4.2 LUNG and AM 4.2MUNG).


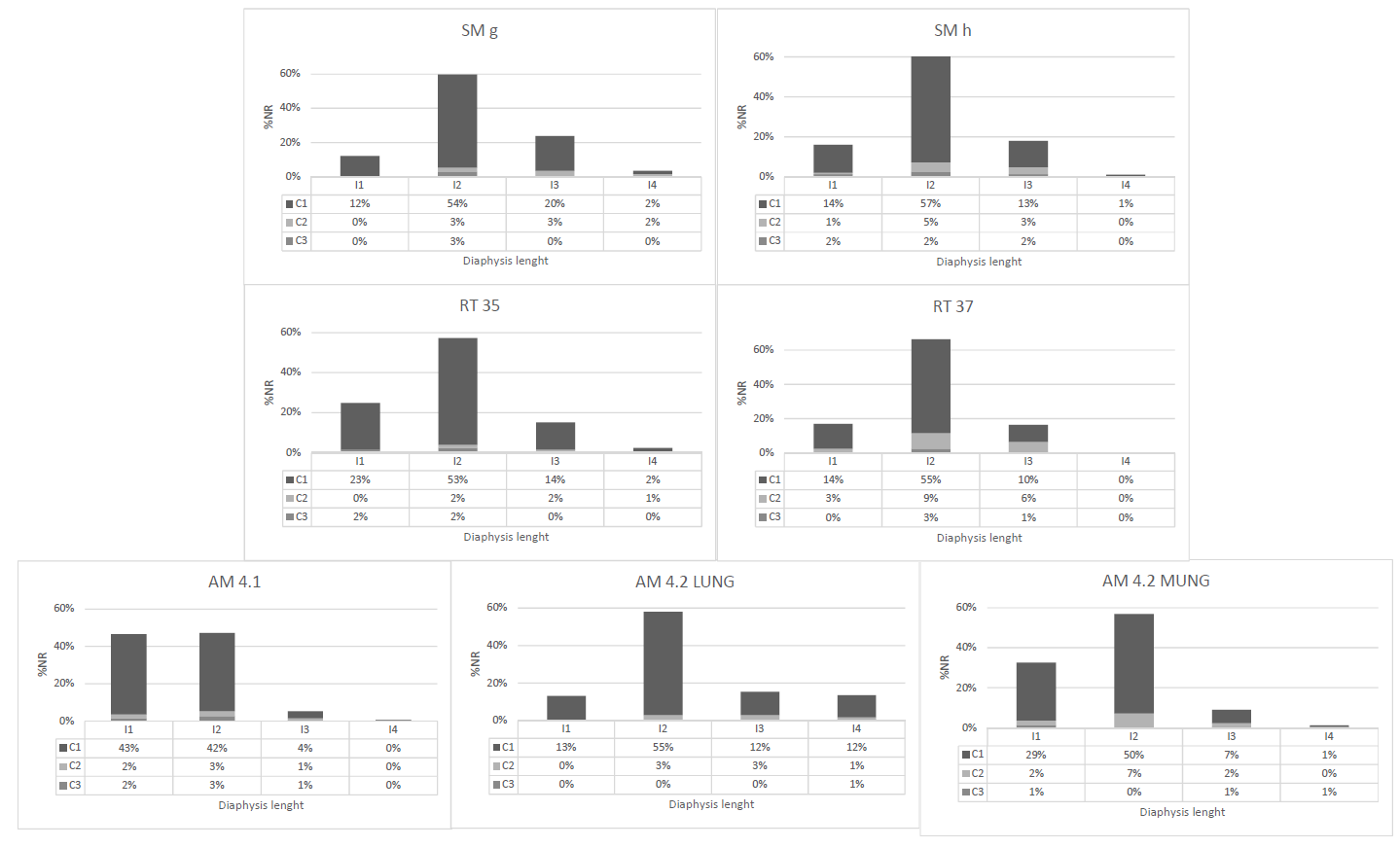


S2 Figure: NISP values for each of the measurement intervals along the length and circumference of the all types of bone together. Saint Marcel Cave level g (SMg); Saint Marcel Cave level h (SMh); Riparo Tagliente level 35 (RT35); Riparo Tagliente level 37 (RT37); Abri du Maras level 4.1 (AM4.1); Abri du Maras level 4.2 (AM4.2 LUNG and AM 4.2MUNG).


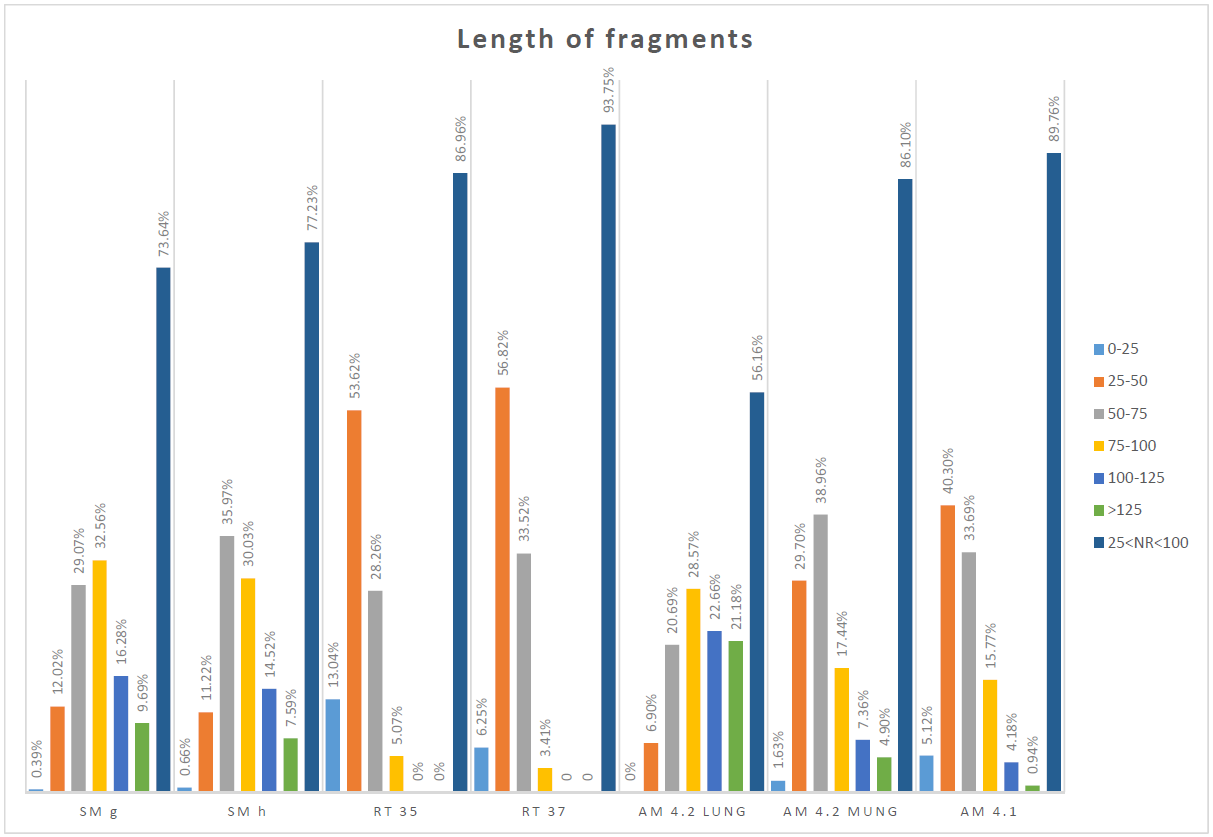


S3 Figure: Diagram showing relative frequencies of shaft length for each levels of our sample of long bone elements. Saint Marcel Cave level g (SMg); Saint Marcel Cave level h (SMh); Riparo Tagliente level 35 (RT35); Riparo Tagliente level 37 (RT37); Abri du Maras level 4.1 (AM4.1); Abri du Maras level 4.2 (AM4.2 LUNG and AM 4.2MUNG).


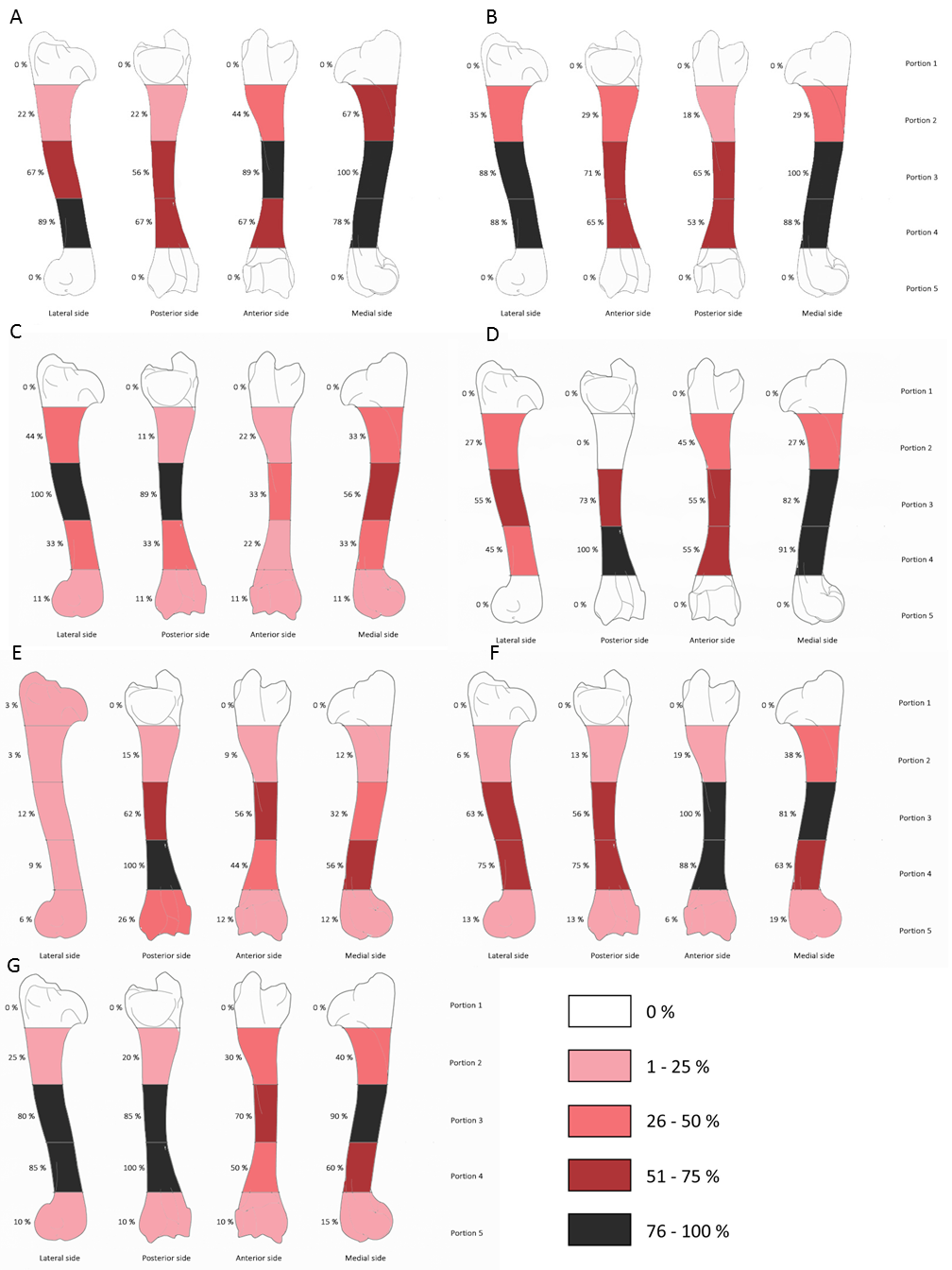


S4 Figure: Percentage of humerus survivorship areas of each sample; Saint Marcel Cave level g (A), level h (B); Riparo Tagliente level 35 (C), level 37 (D); Abri du Maras level 4.1 (E), level 4.2 LUNG (F) and level 4.2 MUNG.


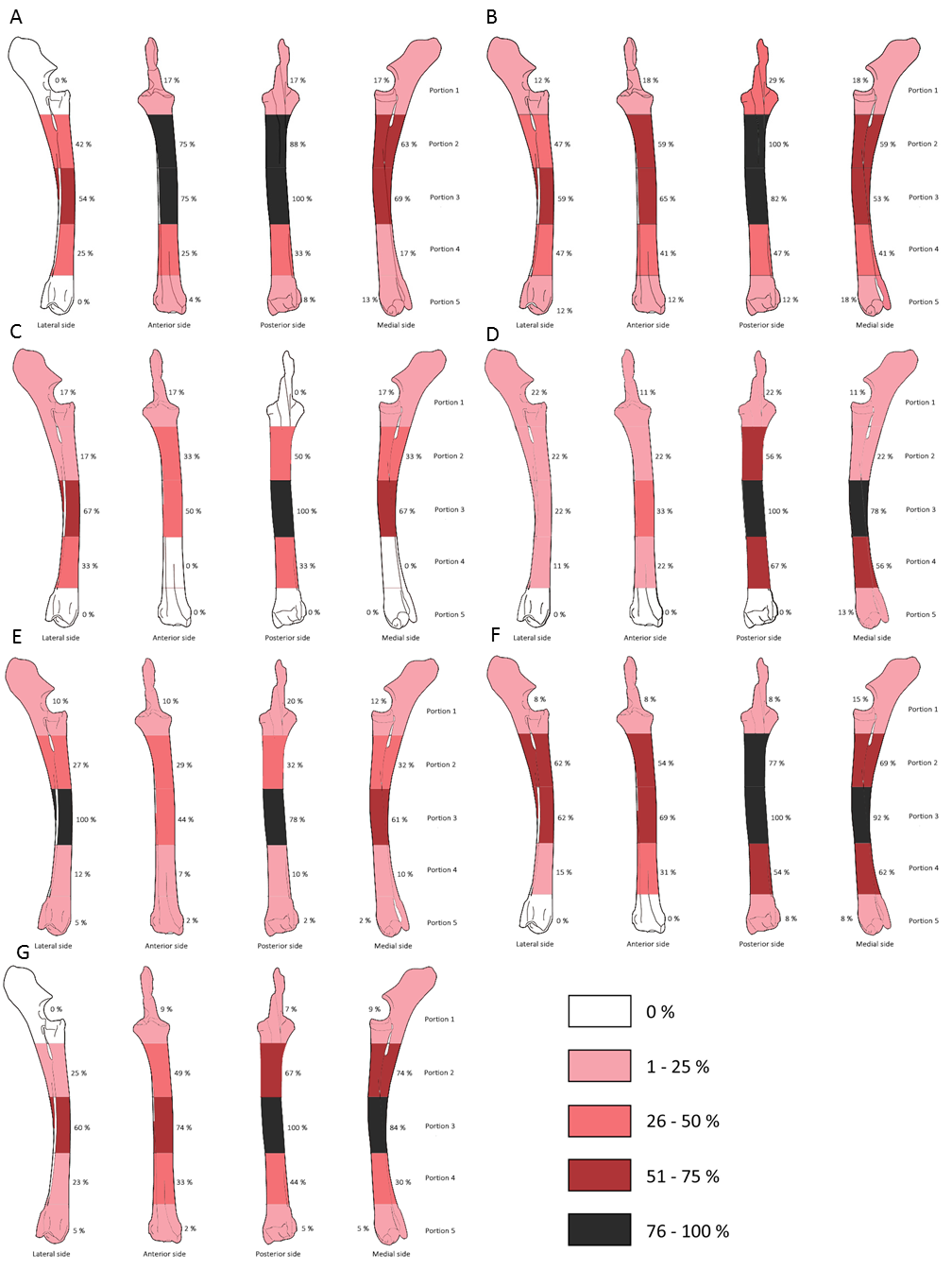


S5 Figure: Percentage of radio-ulna survivorship areas of each sample; Saint Marcel Cave level g (A), level h (B); Riparo Tagliente level 35 (C), level 37 (D); Abri du Maras level 4.1 (E), level 4.2 LUNG (F) and level 4.2 MUNG.


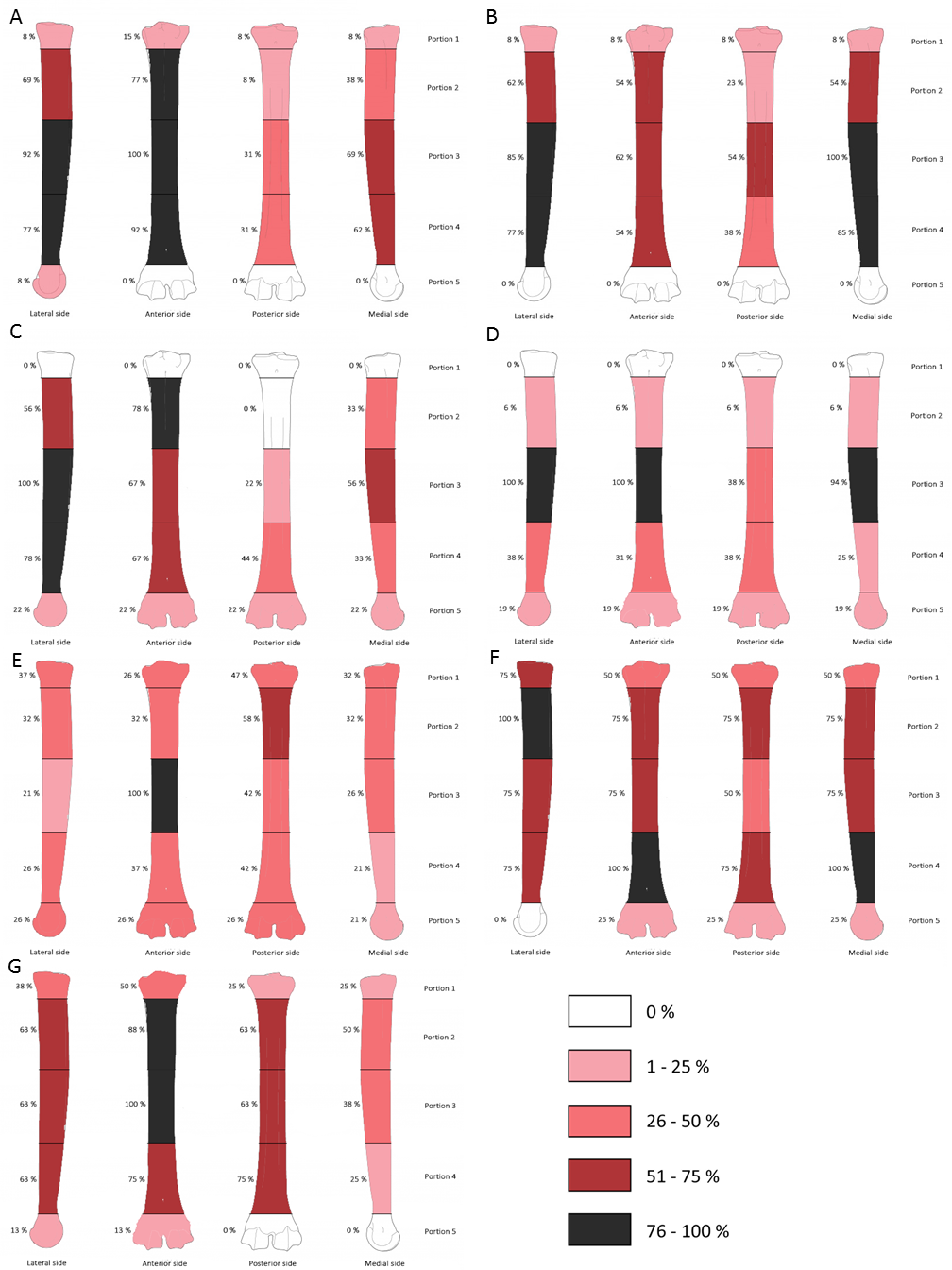


S6 Figure: Percentage of metacarpal survivorship areas of each sample; Saint Marcel Cave level g (A), level h (B); Riparo Tagliente level 35 (C), level 37 (D); Abri du Maras level 4.1 (E), level 4.2 LUNG (F) and level 4.2 MUNG.


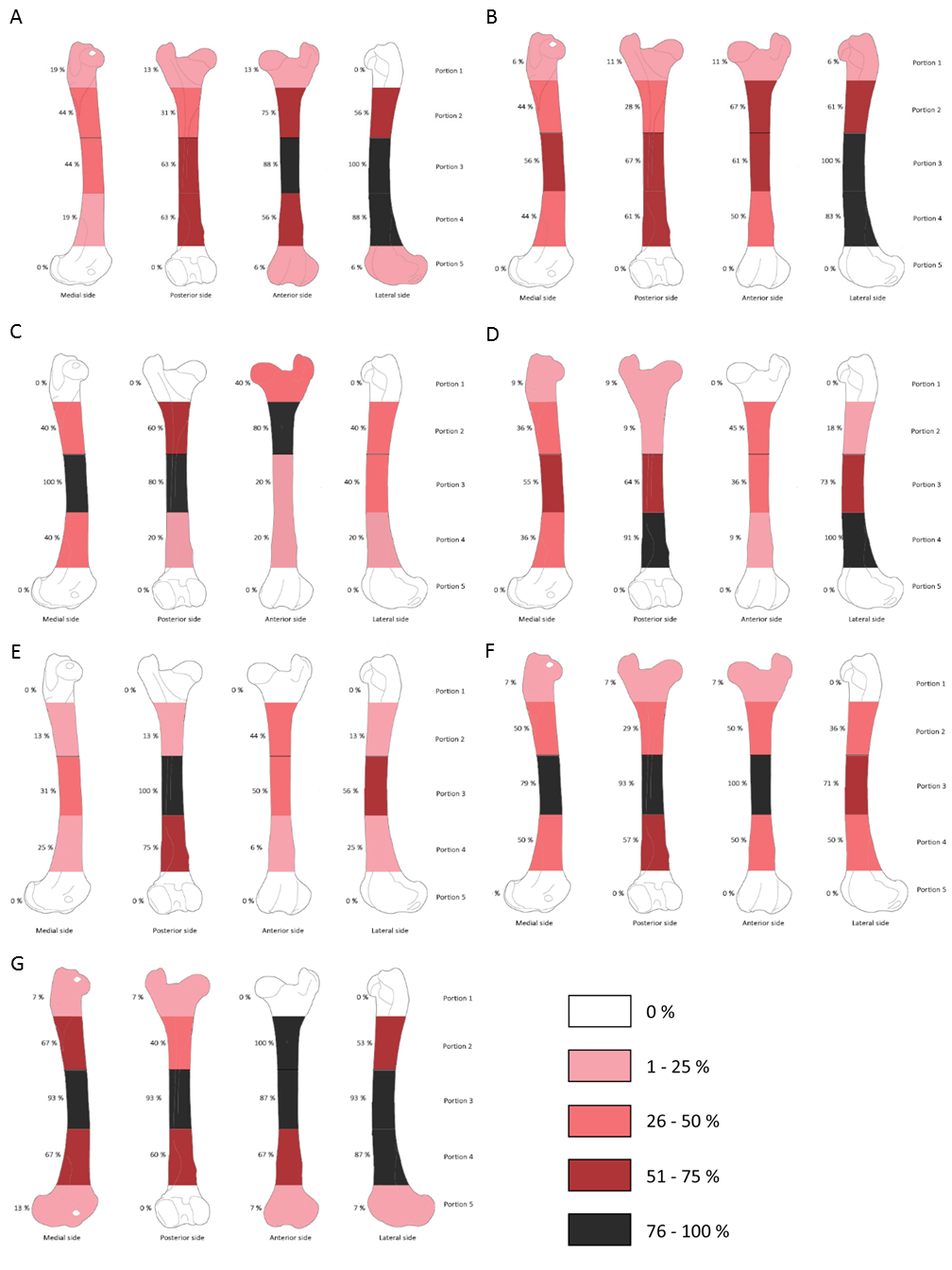


S7 Figure: Percentage of femur survivorship areas of each sample; Saint Marcel Cave level g (A), level h (B); Riparo Tagliente level 35 (C), level 37 (D); Abri du Maras level 4.1 (E), level 4.2 LUNG (F) and level 4.2 MUNG


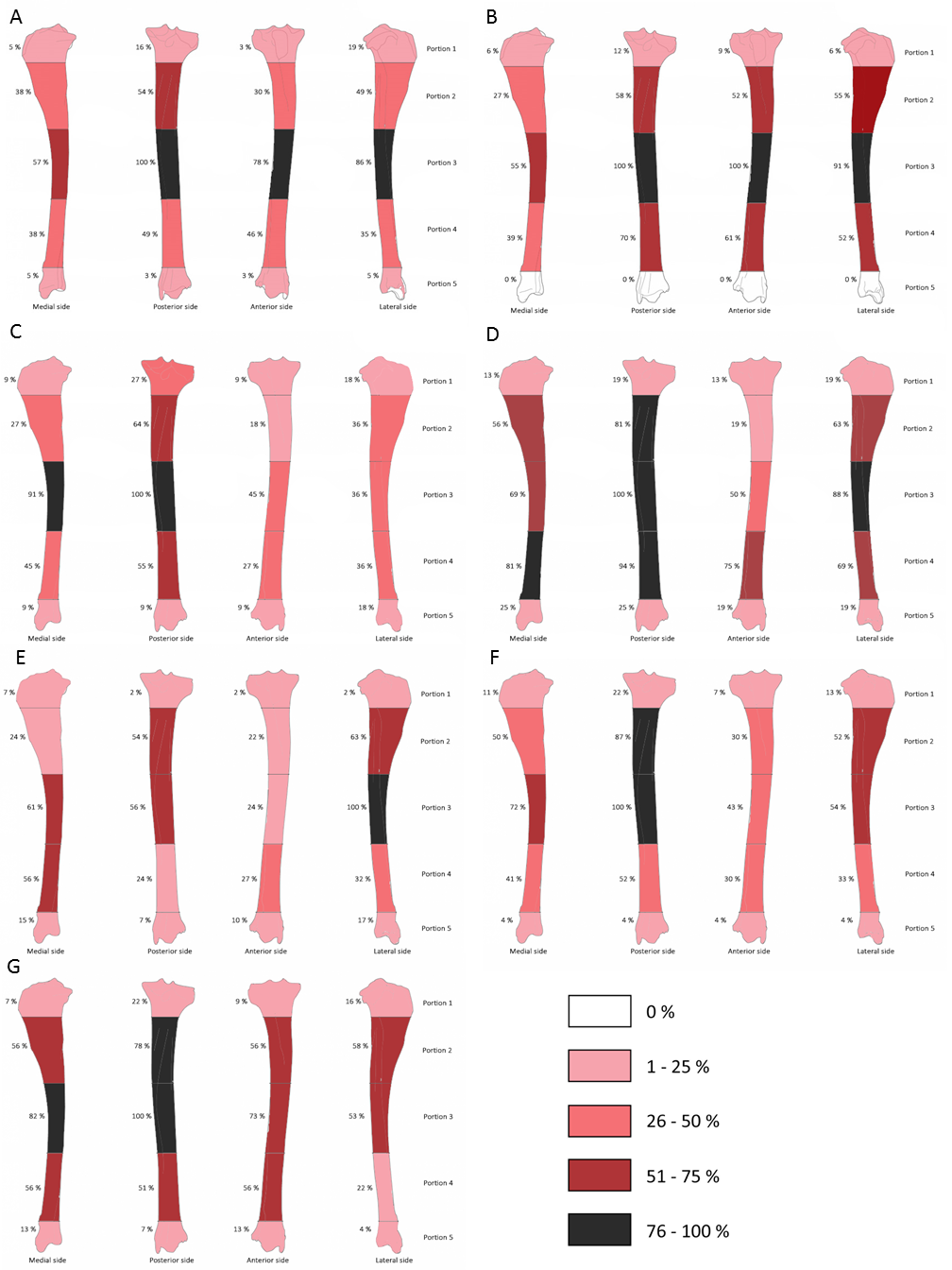


S8 Figure: Percentage of tibia survivorship areas of each sample; Saint Marcel Cave level g (A), level h (B); Riparo Tagliente level 35 (C), level 37 (D); Abri du Maras level 4.1 (E), level 4.2 LUNG (F) and level 4.2 MUNG.


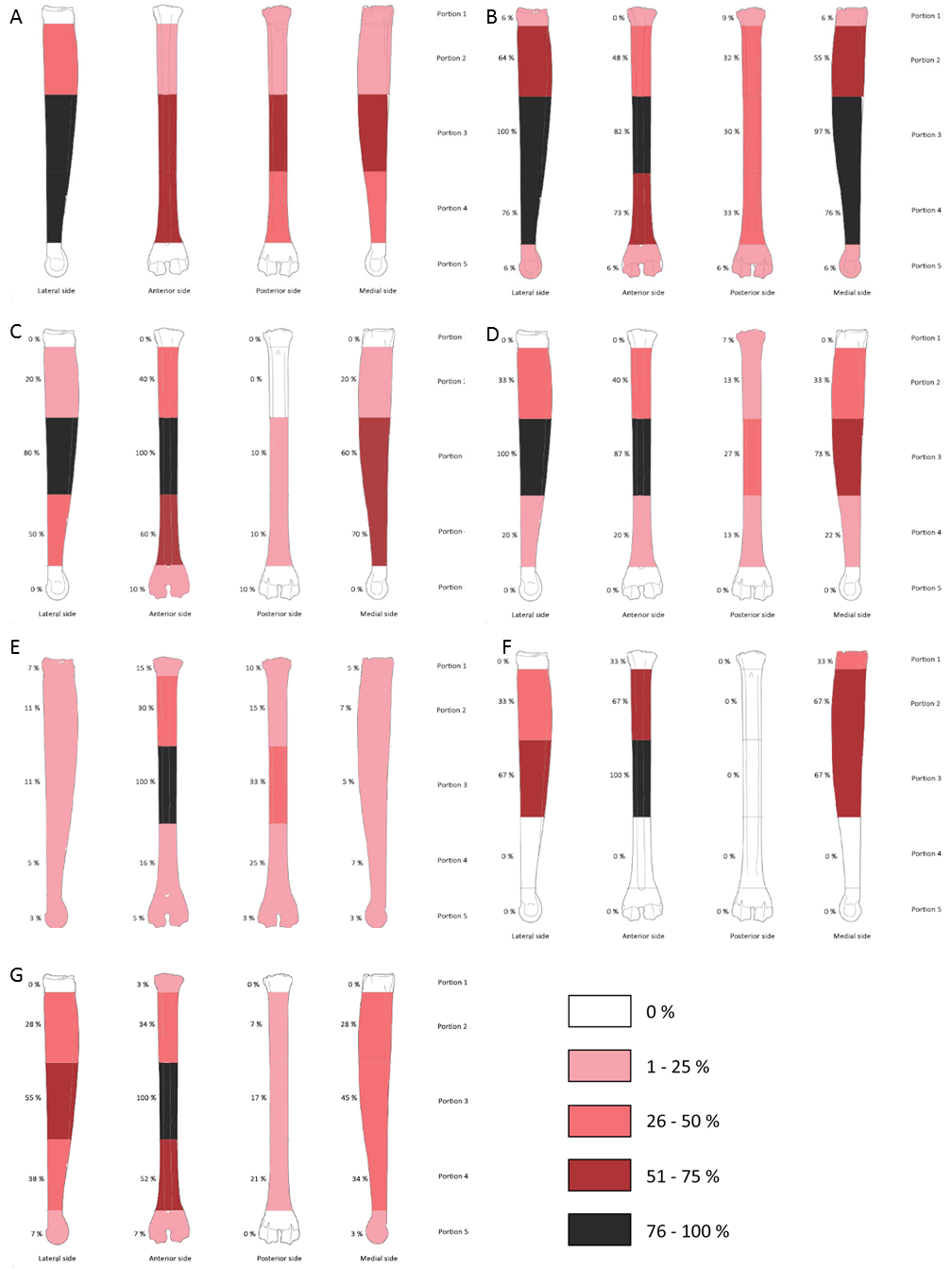


S9 Figure: Percentage of metatarsal survivorship areas of each sample; Saint Marcel Cave level g (A), level h (B); Riparo Tagliente level 35 (C), level 37 (D); Abri du Maras level 4.1 (E), level 4.2 LUNG (F) and level 4.2 MUNG.


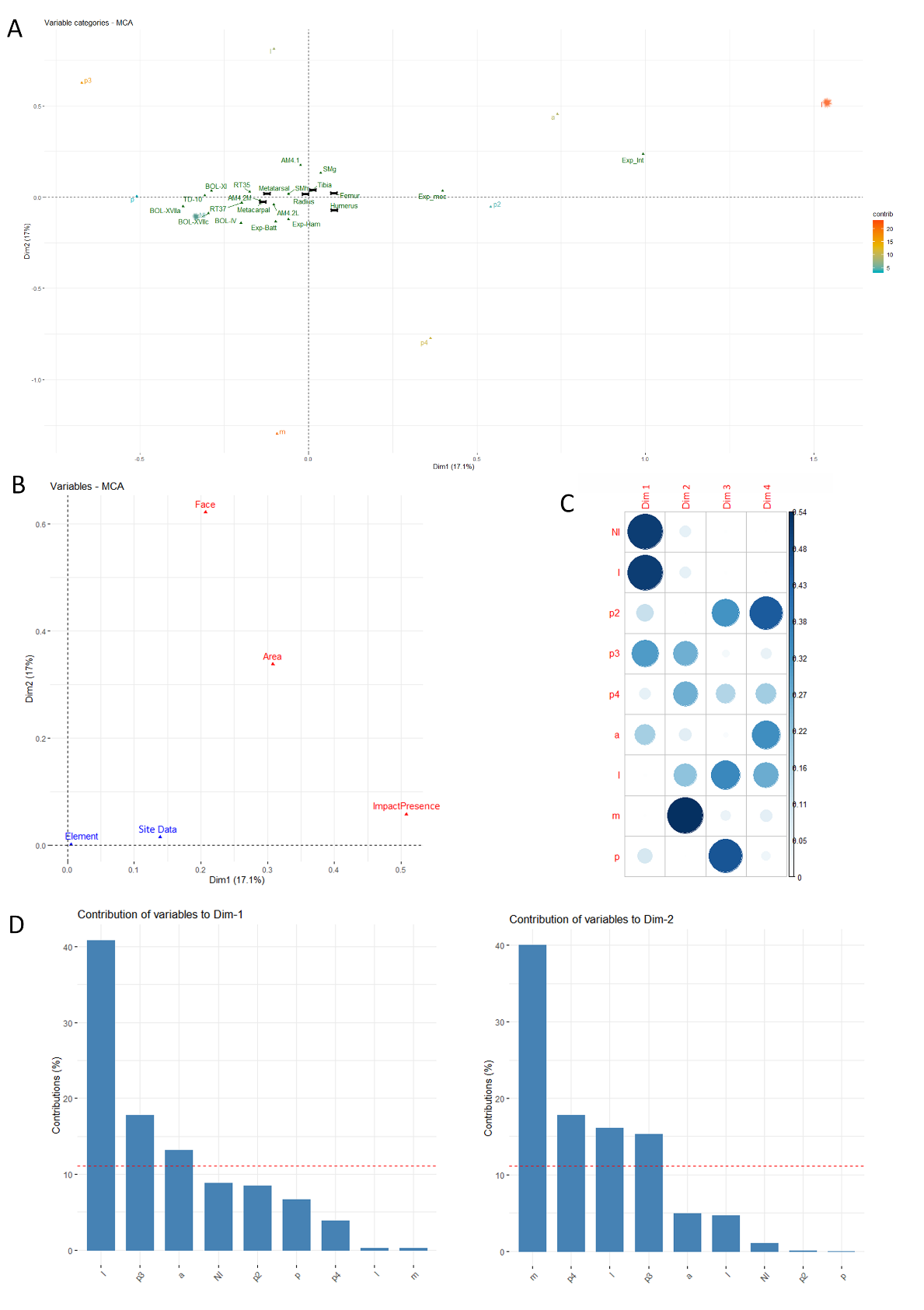


S10 Figure: A- MCA of full data, BOL: Bolomor, TD10: Grand Dolina TD10-1, RT: Riparo Tagliente, AM: Abri du Maras, SM: Saint-Marcel and Exp: Experiment; Int: Intuitivity, Moc: Moclan and Dominguez-Rodrigo 2018, Ham: Hammerstone on anvil and Batt: by batting: Blasco et al. 2013. NI: without impact and I: with impact B- Contribution of variables (in blue, the illustrative ones); C- Contribution of the variables for each dimension; D- Contribution for the first and second dimension


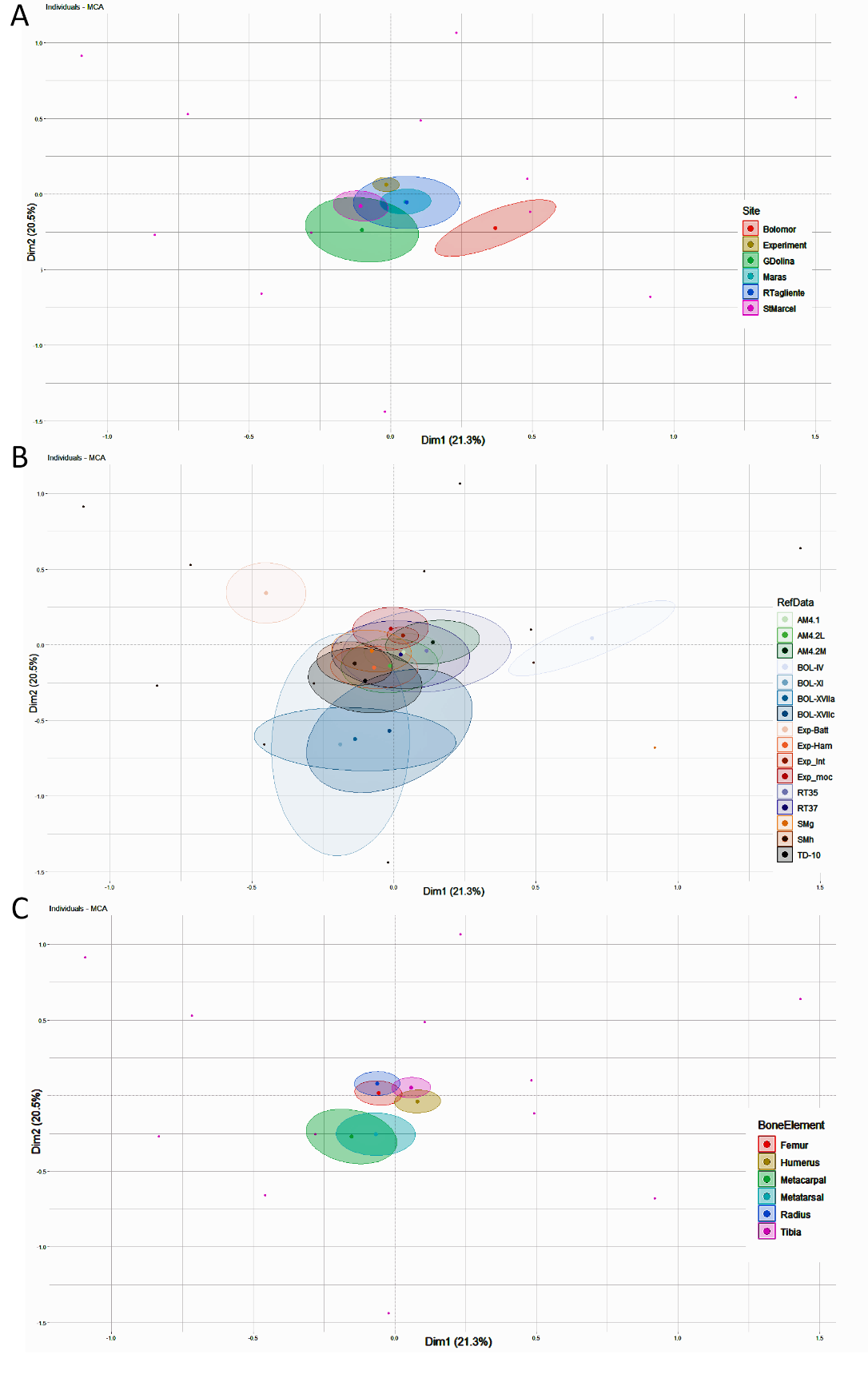


S11 Figure: MCA of full data of our site according to the qualitative and illustrative variables


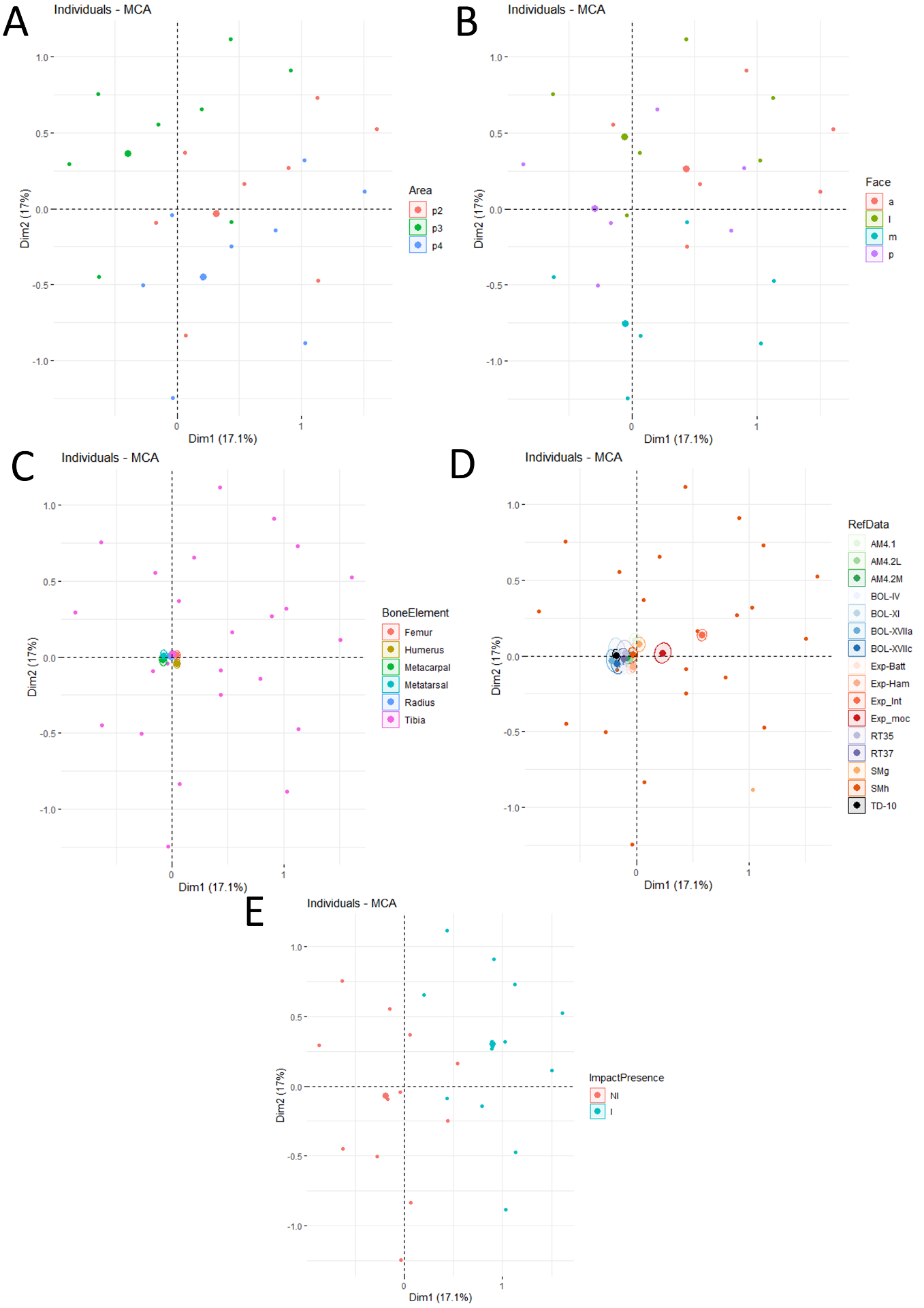


S12 Figure: MCA of all levels and element taking into account only the portion and side with percussion marks

S13 Dataset: Area with and without percussion marks on the sites of Bolomor (levels IV and XIIIc), Saint Marcel Cave (StMarcel, levels g g), Abri du Maras (Maras, level 4.1 and 4.2), Riparo Tagliente (RTagliente, levels 35 and 37) and Gran Dolina (GDolina, levels TD 10) and regarding the experiments Vettese et al. 2020 (Exp_Int), Blasco et al. 2013 (Exp_ham for hammerstone and Exp_bat for batting) and Moclan et al. 2018 (Exp_moc). figshare. Dataset: <https://doi.org/10.6084/m9.figshare.19249544.v1>

S14 Script R: Code available: <https://github.com/AntonyBorel/New-evidence-of-Neandertal-butchery-traditions>
