# Supplementary material for: New evidence of Neandertal butchery traditions through the marrow extraction in southwestern Europe (MIS 5–3)
Source: PLoS One. 2022 Aug 17;17(8):e0271816. doi: 10.1371/journal.pone.0271816 (PMC9385001; doi:10.1371/journal.pone.0271816)
Supplement: Attachment — Submitted filename: PONE-D-21-28076_with comments AV.pdf [file pone.0271816.s002.pdf]

PLOS ONE

New evidence of Neandertal butchery traditions in Southwestern Europe (MIS 3-5)

--Manuscript Draft--

**Manuscript Number:**	PONE-D-21-28076
**Article Type:**	Research Article
**Full Title:**	New evidence of Neandertal butchery traditions in Southwestern Europe (MIS 3-5)
**Short Title:**	New evidence of Neandertal butchery traditions
**Corresponding Author:**	Delphine Vettese<br>University of Ferrara<br>Ferrara, ITALY
**Keywords:**	Middle Palaeolithic; Neandertal; Long bone breakage patterns; Carcass processing; Traditions; Taphonomy
**Abstract:**	Long bone breakage for bone marrow recovering is a commonly observed practice in Middle Palaeolithic contexts, whatever the climatic conditions. While lithic technology has been largely used to define cultural patterns in human groups, butchering techniques has been rarely evaluated until now to track traditions. In this paper, we aim to test the hypothesis of butchery traditions and cultural learning among Neandertal groups thanks to the bone assemblages coming from three sites in Western Europe. These sites are located in south-eastern France and Northern Italy and are dated to the Late Middle Palaeolithic: Abri du Maras (MIS 4, Ardèche), Saint-Marcel (MIS 3, Ardèche), and Riparo Tagliente (MIS 4-3, Verona).<br><br>The detection of culturally-induced patterns on bone breakage involves differentiating them from intuitively generated patterns. To tackle this issue, we used a zooarchaeological approach focusing on the percussion marks produced during the bone breakage process.<br><br>Data demonstrate that the Neandertal groups occupying levels 4.1 and 4.2 of the Abri du Maras and levels g and h at Saint-Marcel in France applied similar butchery traditions to recover the yellow marrow from humeri and femurs, while these both sites have diverse traditions from each other. On the contrary, in Riparo Tagliente, in Italy, several groups or individuals of a same group did not share the same butchery traditions over time. Our research demonstrates that Neandertal groups applied intense standardized bone breakage, far from intuitive practice, related to bone density and/or skeletal morphology, as observed experimentally. These standardized patterns, which are systematic and counter-intuitive, can be interpreted as culturally induced, for the Abri du Maras and Saint Marcel cave. The diversity of traditional Neandertal behaviours should be considered by taking into account the butchery, in particular the practice of bone marrow recovery, and not only technological behaviors and types of tool kits.
**Order of Authors:**	Delphine Vettese
	Antony Borel
	Ruth Blasco
	Louis Chevillard
	Trajanka Stavrova
	Ursula Thun Hohenstein
	Marta Arzarello
	Marie-Hélène Moncel
	Camille Daujeard
**Additional Information:**	
**Question**	**Response**

Powered by Editorial Manager® and ProduXion Manager® from Aries Systems Corporation

**Financial Disclosure**<br><br>Enter a financial disclosure statement that describes the sources of funding for the work included in this submission. Review the submission guidelines for detailed requirements. View published research articles from *PLOS ONE* for specific examples.<br><br>This statement is required for submission and **will appear in the published article** if the submission is accepted. Please make sure it is accurate.<br><br>**Unfunded studies**<br>Enter: *The author(s) received no specific funding for this work.*<br><br>**Funded studies**<br>Enter a statement with the following details:<br>• Initials of the authors who received each award<br>• Grant numbers awarded to each author<br>• The full name of each funder<br>• URL of each funder website<br>• Did the sponsors or funders play any role in the study design, data collection and analysis, decision to publish, or preparation of the manuscript?<br>• **NO** - Include this sentence at the end of your statement: *The funders had no role in study design, data collection and analysis, decision to publish, or preparation of the manuscript.*<br>• **YES** - Specify the role(s) played.<br><br>* typeset	RB:Ramón y Cajal research contract by the Ministry of Economy and Competitiveness (RYC2019-026386-I) and AEI/FEDER, EUproject PID2019-104949GB-I00, and the Generalitat de Catalunya projects 2017 SGR 836 and CLT009/18/00055; CD, DV, TS and LC was supported by the Fondation Nestlé France (SJ 671–16) (https://fondation.nestle.fr/); DV is supported by the Centre d'Information des Viandes – Viande, sciences et société (SJ 334–17); AB, CD, DV, LC, MHM and TS are supported by the Muséum national d'Histoire naturelle.<br><br>The funders had no role in study design, data collection and analysis, decision to publish, or preparation of the manuscript.
**Competing Interests**<br><br>Use the instructions below to enter a competing interest statement for this submission. On behalf of all authors, disclose any competing interests that could be perceived to bias this work—acknowledging all financial support and any other relevant financial or non-financial competing interests.<br><br>This statement is required for submission	The authors have declared that no competing interests exist.

Powered by Editorial Manager® and ProduXion Manager® from Aries Systems Corporation

and **will appear in the published article** if the submission is accepted. Please make sure it is accurate and that any funding sources listed in your Funding Information later in the submission form are also declared in your Financial Disclosure statement.<br><br>View published research articles from *PLOS ONE* for specific examples.<br><br>**NO authors have competing interests**<br><br>Enter: *The authors have declared that no competing interests exist.*<br><br>**Authors with competing interests**<br><br>Enter competing interest details beginning with this statement:<br><br>*I have read the journal's policy and the authors of this manuscript have the following competing interests: [insert competing interests here]*<br><br>* typeset	
**Ethics Statement**<br><br>Enter an ethics statement for this submission. This statement is required if the study involved:<br><br>• Human participants<br>• Human specimens or tissue<br>• Vertebrate animals or cephalopods<br>• Vertebrate embryos or tissues<br>• Field research<br><br>Write "N/A" if the submission does not require an ethics statement.<br><br>General guidance is provided below. Consult the submission guidelines for detailed instructions. **Make sure that all information entered here is included in the Methods section of the manuscript.**	N/A

**Format for specific study types**<br><br>**Human Subject Research (involving human participants and/or tissue)**<br>• Give the name of the institutional review board or ethics committee that approved the study<br>• Include the approval number and/or a statement indicating approval of this research<br>• Indicate the form of consent obtained (written/oral) or the reason that consent was not obtained (e.g. the data were analyzed anonymously)<br><br>**Animal Research (involving vertebrate animals, embryos or tissues)**<br>• Provide the name of the Institutional Animal Care and Use Committee (IACUC) or other relevant ethics board that reviewed the study protocol, and indicate whether they approved this research or granted a formal waiver of ethical approval<br>• Include an approval number if one was obtained<br>• If the study involved *non-human primates*, add *additional details* about animal welfare and steps taken to ameliorate suffering<br>• If anesthesia, euthanasia, or any kind of animal sacrifice is part of the study, include briefly which substances and/or methods were applied<br><br>**Field Research**<br><br>Include the following details if this study involves the collection of plant, animal, or other materials from a natural setting:<br>• Field permit number<br>• Name of the institution or relevant body that granted permission	
**Data Availability**<br><br>Authors are required to make all data underlying the findings described fully available, without restriction, and from the time of publication. PLOS allows rare exceptions to address legal and ethical concerns. See the PLOS Data Policy and FAQ for detailed information.	Yes - all data are fully available without restriction

A Data Availability Statement describing where the data can be found is required at submission. Your answers to this question constitute the Data Availability Statement and **will be published in the article**, if accepted.<br><br>**Important:** Stating 'data available on request from the author' is not sufficient. If your data are only available upon request, select 'No' for the first question and explain your exceptional situation in the text box.<br><br>Do the authors confirm that all data underlying the findings described in their manuscript are fully available without restriction?	
**Describe where the data may be found in full sentences. If you are copying our sample text, replace any instances of XXX with the appropriate details.**<br><br>• If the data are **held or will be held in a public repository**, include URLs, accession numbers or DOIs. If this information will only be available after acceptance, indicate this by ticking the box below. For example: *All XXX files are available from the XXX database (accession number(s) XXX, XXX.*).<br>• If the data are all contained **within the manuscript and/or Supporting Information files**, enter the following: *All relevant data are within the manuscript and its Supporting Information files.*<br>• If neither of these applies but you are able to provide **details of access elsewhere**, with or without limitations, please do so. For example:<br><br>*Data cannot be shared publicly because of [XXX]. Data are available from the XXX Institutional Data Access / Ethics Committee (contact via XXX) for researchers who meet the criteria for access to confidential data.*<br><br>*The data underlying the results presented in the study are available from (include the name of the third party*	All relevant data are within the manuscript and its Supporting Information files.

and contact information or URL).<br>• This text is appropriate if the data are owned by a third party and authors do not have permission to share the data.<br><br>* typeset	
Additional data availability information:	

Powered by Editorial Manager® and ProduXion Manager® from Aries Systems Corporation

Manuscript

Click here to access/download;Manuscript;New evidence of butchery traditions within the Neandertal.docx

New evidence of Neandertal butchery traditions in Southwestern Europe (MIS 3-5)

Delphine Vettese[1,2,3], Antony Borel[1,4], Ruth Blasco[5,6], Louis Chevillard[1], Trajanka Stavrova[1], Ursula Thun Hohenstein[2], Marta Arzarello[2], Marie-Hélène Moncel[1], Camille Daujeard[1]

[1]Histoire Naturelle de l'Homme Préhistorique (HNHP, UMR 7194), Muséum national d'Histoire naturelle (MNHN), CNRS, Université de Perpignan Via Domitia, Institut de Paléontologie Humaine, 1 rue René Panhard, 75013 Paris, France
[2]Dipartimento di Studi Umanistici, Università degli Studi di Ferrara, Corso Ercole I d'Este 32, 44121 Ferrara, Italia
[3]Grupo de I+D+i EVOADAPTA (Evolución Humana y Adaptaciones Económicas y Ecológicas durante la Prehistoria). Dpto. Ciencias Históricas. Universidad de Cantabria, Santander, Spain
[4]Institute of Archeological Sciences, Eötvös Loránd University, Múzeum krt. 4/B, 1088 Budapest, Hungary
[5] Institut Català de Paleoecologia Humana i Evolució Social (IPHES-CERCA), Zona Educacional 4, Campus Sescelades URV (Edifici W3), 43007 Tarragona, Spain
[6]Universitat Rovira i Virgili, Departament d'Història i Història de l'Art, Avinguda de Catalunya 35, 43002 Tarragona, Spain

Abstract:

Long bone breakage for bone marrow recovering is a commonly observed practice in Middle Palaeolithic contexts, whatever the climatic conditions. While lithic technology has been largely used to define cultural patterns in human groups, butchering techniques has been rarely evaluated until now to track traditions. In this paper, we aim to test the hypothesis of butchery traditions and cultural learning among Neandertal groups thanks to the bone assemblages coming from three sites in Western Europe. These sites are located in south-eastern France and Northern Italy and are dated to the Late Middle Palaeolithic: Abri du Maras (MIS 4, Ardèche), Saint-Marcel (MIS 3, Ardèche), and Riparo Tagliente (MIS 4-3, Verona).

The detection of culturally-induced patterns on bone breakage involves differentiating them from intuitively generated patterns. To tackle this issue, we used a zooarchaeological approach focusing on the percussion marks produced during the bone breakage process.

Data demonstrate that the Neandertal groups occupying levels 4.1 and 4.2 of the Abri du Maras and levels g and h at Saint-Marcel in France applied similar butchery traditions to recover the yellow marrow from humeri and femurs, while these both sites have diverse traditions from each other. On the contrary, in Riparo Tagliente, in Italy, several groups or individuals of a same group did not share the same butchery traditions over time. Our research demonstrates that Neandertal groups applied intense standardized bone breakage, far from intuitive practice, related to bone density and/or skeletal morphology, as observed experimentally. These standardized patterns, which are systematic and counter-intuitive, can be interpreted as culturally induced, for the Abri du Maras and Saint Marcel cave. The diversity of traditional Neandertal behaviours should be considered by taking into account the butchery,

in particular the practice of bone marrow recovery, and not only technological behaviors and types of tool kits.

Keywords: Middle Palaeolithic, Neandertal, Long bone breakage patterns, Carcass processing, Traditions, Taphonomy

Introduction

Over the last two decades, the diversity and flexibility of the Neandertal diet have been widely demonstrated [1–9]. The ability to use and to cook all the available resources in the environment suggests a combination of complex subsistence behaviours and opportunism [10–13]. Isotopic analyses show a dietary strategy based ~~above all~~ on animal protein[14–16]. In addition to the consumption of meat from large and small game, bone assemblages in Neandertal sites indicate widespread fat consumption [9,17–21]. For example, red and yellow bone marrow ~~were~~ an important food resource, especially in ~~a~~ dry and cold environment[22–25]. Given ~~that to~~ the large accumulation of broken bones in Neandertal sites, focusing on the modalities of bone fracturing seems to be an innovative way of tracking traditional butchery practices among Neandertal groups [1,26–32].

For decades, analyses of Neandertal traditions were based on studies of lithic assemblages, which were assumed to record the characteristics of a group with the inter-generational transmission of knowledge [42–44]. Stone tools are even used as markers of a specific group or regional traditions. More and more, lithic analyses focus on the hypothesis that stone tool corpuses may indicate both activities and traditions. Regarding the analyses of faunal assemblages, the identification of traditions is mostly tested through studies of hunting strategies [30,45–48] while butchery techniques are ~~very~~ rarely studied.

In this paper, we consider that a butchery tradition is brought to light by observing standardized and counter-intuitive patterns showing that an inter-generational group share the same butchery know-how. Intuitiveness is the immediate apprehension of a non-trained butcher to break a bone. It could be influenced by anatomical constraints and other characteristics of the bone [33,49]. Some experiments focusing on the intuitive way to extract marrow highlighted intuitive patterns of percussion mark distribution [50]. Thus, the development of specific butchery skills, which could be applied to Neandertals who regularly broke long bones to extract yellow marrow, is a valuable hypothesis [51]. Hence, transmitted skills include habits enhanced by experience and/or group traditions. Butchery traditions imply know-how

(sequences of gestures), dedication (time involved in the activity) and skill (ability to reproduce gestures and to correct them) [52].

Blasco et al. [33] ~~applied,~~ for the first time, the hypothesis of butchery practices to two Middle Palaeolithic Spanish sites (MIS 5-9): Bolomor (levels IV, IX, XIIa and XIIc) and Gran Dolina (TD10-1 [faunal sample from field-work seasons 2000-2001]). They highlighted the presence of systematic and counter-intuitive percussion mark patterns in level IV of Bolomor, indicating specific butchery traditions. On the contrary, in the earlier TD10-1 level of Gran Dolina, they could not demonstrate cultural know-how but brought to light a probable palimpsest combining various practices. After their innovative paper, a few other analyses tried to demonstrate the existence of traditional butchery practices to recover yellow marrow in the Southern Mediterranean area [26,34].

Unlike percussion marks, cut marks appeared ~~to be more difficult to analyse~~ standardized butchery practices, mainly because of the role of the butcher's dexterity, animal morphology and size, the raw materials and tools used, and the frequency and location of cutting incisions [35,36]. Indeed, cut marks made during carcass processing may be scarce for experimented butchers and are mainly accidental because most of the cutting focus on soft tissues and not directly on bones. However, the direct percussion of long bones is a direct action on bone when almost all the soft tissue are already removed [24,26,33,37,38]. Besides, percussion marks are part of a complex "*chaîne opératoire*" where the bone can be considered as a raw material both for alimentary purposes and for tool production, i.e., bone retouchers or hammers for Neandertals [39–41].

Considering the degree of cognition, ~~specific~~ butchery traditions should be embedded ~~in~~ the socio-cultural practices of Neandertal groups, in the same way as hunting strategies and the cultural traditions of lithic tools. To test this hypothesis, we focus on three late Neandertal sites with anthropogenic faunal accumulations: Abri du Maras and Saint-Marcel Cave in south-eastern France, and Riparo Tagliente in Italy. In order to monitor possible behavioural changes in butchery practices over time, we selected two layers from each site, all dated between MIS 4 and 3 (Figure 1). We only focus on percussion mark distribution produced by yellow marrow recovery, after the evaluation of the impact of taphonomic alterations on the archaeological series.

The Middle Palaeolithic faunal assemblages of Abri du Maras, Saint-Marcel Cave and Riparo Tagliente are appropriate because they are almost exclusively anthropogenic accumulations, with a virtual absence of carnivore marks on bones (e.g.,[53–56]). The faunal remains are well preserved and a complete butchery "*chaine opératoire"* was conducted *in situ*. Moreover, the assemblages come mainly from recent

excavations where all the bones were systematically recorded, including the smallest bone fragments. For almost all of them, the faunal spectrum is practically monospecific (except the level 4.2 of Maras), with the predation of cervids (roe deer, red deer and reindeer), which is propitious to the comparison of hunting strategies. Our results are then compared with previous data published by Blasco et al. [23]and with our own experimental data *e.g.*: [50].

Figure 1: Location of the studied sites with the hunted species and an illustration of the lithic culture. Drawing by M. Arzarello, R. Gilles and A. Theodoropoulou.

Archaeological background

Abri du Maras layers 4.1 and 4.2

The site of Abri du Maras is located on the right bank of the Middle Rhône Valley, in a small dry valley of the Ardèche River, 70 m above the river. It constitutes the remains of a huge, former south-east facing rock shelter[27,43,53,57]

The first archaeological investigations revealed eight layers with Middle Palaeolithic deposits, with Levallois laminar debitage for layer 1 [57–60]. The new excavation only concerned the middle and lower parts of the sequence, from previously assigned layers 4 to 8. The stratigraphic unit 6 was at the bottom of the sequence. The overlying unit 4 consisted of a silt and sandy-silty sedimentary accumulation (0.5 to 1 m thick), with two main phases of human occupations (4.1 and 4.2), with traces of combustion and occasional diffuse ash lenses. Unit 4 was excavated over an area of more than 50 m² [61]. There was no significant orientation of the material suggesting that no major disturbance of the archaeological remains occurred[62].

U/Th dating applied to bones from the top of unit 5/bottom of unit 4 yielded ages of 72 ± 3 ky, 87 ± 5 ky, 89 ± 4 ky, and 91 ± 4 ky [60,63]. New preliminary ESR-U/Th dating of ungulate teeth confirmed the chronology of unit 5 (90 ± 9 ky), but gave more recent ages for unit 4, attributed to MIS 3. Layer 4.1 (upper part of unit 4) thus dates to between 40 ± 3 ky and 46 ± 3 ky (n=2) (also MIS 3), while samples from layer 4.2 provide ages ranging from 42 ± 3 ky to 55 ± 2 ky (n=3) (MIS 3) [64].

The varied faunal spectrum of the unit 4 is composed, in order of abundance, of *Rangifer tarandus, Equus ferus* cf. *germanicus, Cervus elaphus, Bison priscus, Capra ibex, Equus hydruntinus and Megaloceros giganteus* [27,43,54,55]. Carnivore remains were lacking. Contrary to layer 4.1, layer 4.2 does not display

a mono-specific spectrum [54]. Large ungulates as horses and bisons, and middle-sized ungulates as reindeer and red deer are the most predominate the faunal spectrum.

Lithic analysis revealed that the technical strategies applied mainly to flint and secondarily to other stones (quartz, quartzite and granite) involved the fragmentation of reduction processes into local and semi-local perimeters around the site [61]. The main methods of core exploitation are Levallois, discoidal and expedient, generally on cortical flint cores on flakes. Flint flakes, secondary blades and points are the main components. The largest flint flakes, Levallois blades and points were brought to the site from up to 20-30 km away (north and south) from the site. Flake-tools are very rare (3% for layer 4.1 and 4.8% for layer 4.2), and the retouch never modified the shape of the pieces, except for one and two Quina scrapers made on a thick and wide flint flake. In layer 4.1, 32 pebbles and in layer 4.2, 21 pebbles, sometimes broken or with a single removal, were introduced, possibly as hammerstones, although they bear few percussion marks. These quartz, quartzite, limestone, basalt, granite and rarely flint pebbles were collected in the surrounding rivers. They measure mainly between 60 and 120 mm and are round or oval. The lithic analyses showed the anticipation of domestic needs for butchery, plant and woodworking [65–67]. Neandertals exploited a wide range of resources including large mammals, fish, ducks, raptors, rabbits, mushrooms, plants and wood. Evidence of impact fractures suggests that some points could have been used as projectile tips. They were either brought to the site as points or prepared on the site [65].

Saint Marcel cave layers g and h

The site of Saint-Marcel is located at the end of the Ardèche Gorges, not far from the Abri du Maras, on the north side of the gorge, approximately 200 m inland from the Ardèche River. The deposits lie under the cave overhang, in a very large, sub-horizontal, south-facing section, 12.5 m wide, 7 m deep and 3 m high. Excavations of the Mousterian layers were carried out by R. Gilles from 1974 to 1988, during which a surface area of 30 m^2 was exposed, reaching a depth of nearly 7 m. Two main stratigraphic units were defined. The upper one contains the twelve Mousterian occupation phases [57,68].

The lower layers (k to u) were attributed to MIS 5 based on the sedimentological analysis [57]. The paleontological analysis of the faunal remains clearly set layer u apart from all the others, given the abundance of fallow deer in this layer, with *Hemitragus cedrensis*, as well as a similar red deer to that found at the site of Les Cèdres (Var, France). These point to an Eemian occupation for layer u [69–71]. Paleontological, environmental and sedimentological analyses attribute Unit 7 (layers j to g) to a warm episode, either MIS 3 or the end of MIS 5 [55,57,72–75]. There seemed to be no hiatus in sedimentation

between layers j and g, nor between g and f [55,57,73]. ^{14}C results on bones from layer f [76] were statistically identical to each other: 37 850 ± 550 BP and 37 850 ± 600 BP. The third, 41 300 ± 1700 BP, overlaps them at two standard deviations. These results indicated that layer f of Saint-Marcel did indeed date to the late Mousterian.

In all of the upper layers at Saint-Marcel, red deer represents more than 80 % of the number of identified specimens (NISP), with an exception of the level u where the fallow deer is dominant. Neandertals practised selective hunting of prime-aged adult red deer [55,72,73]. Carcass butchery was particularly intensive. Cut marks and percussion marks were recorded on just over half the identified specimens in the faunal assemblage and more than 70 % bore evidence of green-bone fractures. Topographical and zooarchaeological data showed that all of the layers were associated with long-term occupation. In addition, the high proportion of bone retouchers (n = 303, representing nearly 6 % of the total number of remains with a size superior to 5 cm (NR)) makes this site distinctive.

The lithic assemblages show marked homogeneity throughout the stratigraphic sequence. Flint, which was collected in various forms (as pebbles, nodules and tablets) was the main raw material used, and was procured from a variety of sectors (in the Barremian-Bedoulian from the plateau to the north, from the Orgnac plateau to the south and in the Rhone Valley to the east) [68,77]. The richest phase of occupation correspond to the layers i and h, in the middle part of the sequence. Most of the flint processing probably took place at the site; the entire *chaîne opératoire* is represented. The lithic assemblage from all the layers consisted mainly of debitage products, especially flakes. These were small in size, ranging from 30 to 50 mm long, on average. They could mostly be attributed to discoid debitage (centripetal, unipolar or bipolar method), carried out especially on the ventral surface of flakes. In some cases, they strike along a third plane, resulting in the extraction of thick flakes with a triangular cross-section ("crested flake") [68]. Very few retouched tools were found at this site (approximately 5%), consisting primarily of scrapers, in addition to some convergent pieces. Retouch was light and did not modify the blank, although some thinning is present.

For the other raw materials, the number of lithic objects is low and consists of some entire pebbles and above all pebble fragments/flakes in quartz, limestone and quartzite. Little evidence of the role of these raw materials was identified with remains of hammerstones or introduction of some flakes.

Riparo Tagliente layers 35 and 37

Riparo Tagliente is a rock shelter located in the city of Stallavena di Grezzana (Verona, N-E Italy). It is located on the west slope of Valpantena, one of the main valley-bottom of the pre-Alpine massif of

Monti Lessini at an altitude around 250 m. Situated at the bottom of a rock wall, it was formed by limestone, the valley bottom [78–80].

The site was discovered in 1958 by F. Tagliente and excavated by the Museo di Storia Naturale di Verona from 1962 to 1964 [81–84]. In 1967, the University of Ferrara taken over the excavation of the site and pursue it until now. The systematic excavation provided an important stratigraphic series of deposit attributed to the Upper Pleistocene, within a high anthropic occupation[85].

Until the mid-seventies research focused mostly on the excavation of a long trench running transversally to the rock-shelter and on a test-pit located in the most internal area (Southern sector, cf. Figure 1 in [86]). Two archaeological units separated by a river escarpment were excavated. The lower unit was occupied during the MIS 4-3, this was characterized by Mousterian and Aurignacian lithic industries and an upper unit occupied during the Late Glacial, which provided an Epigravettian industry. The layer 35 and 37 belonged to the upper layers of the Mousterian deposit, characterised by loess sediment. No radiometric dating was performed yet, the dating estimation of the upper unit was based on the analyses of the lithic and faunal assemblages [79].

Regarding the small mammals, the remains found were mostly teeth [79]. The assemblages of the upper Mousterian layer were dominated by *Microtus arvalis* but some species indicated, especially for the layer 37, a cold environment (*Microtus oeconomus, Microtus gregalis, Ochotona sp.* and *Sicista sp*.). While, in the layer 35 these species were replaced by species indicating a few increase of the temperatures (*Chionomys nivalis* and *Apodemus sp*.) [87].

The ungulates dominated the faunal spectrum of the two studied layers. *Capreolus capreolus* is the most represented species, *Cervus elaphus, Capra ibex and Rupicapra rupicapra* are following regarding the Number of Identified Species. Among the few remains of carnivores, *Canis lupus* and *Ursus arctos* were the most represented. Few remains of *Marmota marmota* have been identified, ~~whom~~ a mandible with cut marks and few undetermined remains of *Aves* and only one of *Lepus sp.* The presence of foetal or neonatal cervid bones suggest an occupation of the rock shelter mainly during the spring [56,88]. Long bones constitute the majority of the faunal assemblage ~~remains~~ of the layer 35 and 37. Some isolated teeth and remains of axial element were identified. In addition, few tarsals and carpals were characterized and numerous phalanxes.

Within the layer 37, two teeth attributed to *Homo neanderthalensis* were identified [86].

In layer 37 and the layer 35, the use of different reduction methods on local raw materials (chert) characterized the Mousterian sequence. These cherts were collected in the neighborhood of the site mostly in secondary position. The opportunistic method was the best represented (c.f. S.S.D.A, [89]). Lithic analyses highlight also the presence of discoid and Levallois method with the lineal and recurrent modalities, in the upper layers the unipolar recurrent became predominant. The retouched blanks are very numerous and mainly represented by side scrapers and denticulates [90–92]. The presence of a volumetric laminar debitage starting from layer 37 represented one of the main particularities of the lithic assemblage [91].

Very few cobbles have been found in the Mousterian layers and they all show percussion marks; they have consequently been interpreted as hammerstone. All pebbles are mostly spherical and have a diameter between 60 and 85 mm. They have been collected in the surrounding of the site and are mainly in quartzite (only one is on limestone). In the layer 35 any cobble has been found and in the layer 37, within the internal sequences, 3 pebbles have been found.

Material and Methods

Samples selection

The Number of Remains (NR); the Number of anatomically Identified Specimens (NISPa); the Minimum Number of Elements (MNE); the Minimum Number of Individuals (MNI) and the Minimum Animal Unit (MAU) were the count indexes used in this work [24,37,93]. ~~In addition~~, the MNE was calculated based on the count of the preserved areas (cf. Figure 2 in [26]) [33]. ~~For purposes of the~~ taxonomical and anatomical determination, we used both anatomical atlas and comparative collections located at the University of Ferrara and at the IPH (Institut de Paléontologie Humaine, Paris France), e.g. [94–96]. ~~In addition~~, we based our determination on paleontological and previous zooarchaeological studies [54,55,97]. Each identified faunal remains were grouped in three main size category: Large-sized ungulates (LUNG), Medium-sized ungulates (MUNG) and Small-sized ungulates (SUNG) [47,98] (S1 Table).

We follow the methodology established by Vettese et al. [99] to analyze percussion mark distribution. Our work is a continuation of these papers, focusing on the comparison of percussion mark location on long bone shafts. For this reason, only long bone remains identified at least anatomically are taken into account, in order to accurately position percussion traces on bone. In our work, we distinguish

long bone size categories for analytic purposes, i.e., SUNG, MUNG and LUNG. Within a given class size category, only samples with an MNE greater than 100 are selected.

For this study, we took into account the faunal assemblages from Abri du Maras (layers 4.1 and 4.2), Saint Marcel Cave (layers g and h) and Riparo Tagliente (layers 35 and 37). For the purposes of an intra-site comparison, we selected two relatively stratigraphically close layers from each site, coming from the same unit. The layers chosen were highly anthropized, with butchery marks and the virtual absence of carnivore activity. The whole material represented more than 2,180 remains.

The results concerning the percussion marks in layer 4.1 of the Abri du Maras were previously presented in [26]. For comparative purposes, we included the results relating to fragmentation, taphonomy and percussion mark location in the present analyses. Regarding the faunal spectrum of layer 4., we divided this assemblage into two different samples named 4.2LUNG and 4.2MUNG. They constituted respectively 39% and 51% of the Number of taxonomically Identified Specimens (NISPt). The sample from this layer provided 570 remains, including 203 4.2LUNG and 367 4.2MUNG.

The studied material from Saint Marcel Cave comes from layers g and h. These two layers were dominated by the MUNG, in particular by the red deer (layer g: $NISPt_{MUNG}$ 91% of the $NISPt_{Total;}$ layer g: $NISPt_{MUNG}$ 87% of the $NISPt_{Total}$) [55]. Our analyses were carried out on 258 remains from layer g and 303 long bone remains from layer h.

The studied material from Riparo Tagliente comes from layers 37 and 35. Most of the sample consists of roe deer for both layers and is completed by ibex and chamois remains (layer 37: $NISPt_{SUNG}$ 44% of the $NISPt_{Total;}$ layer 35: $NISPt_{SUNG}$ 90% of the $NISPt_{Total}$). SUNG long bone remains dominated the faunal assemblages of these layers. ~~Therefore,~~ we took into account 176 SUNG remains from layer 35 and 138 remains from layer 37.

Taphonomy

The cortical surface of each remain was observed with the naked eye and a 15-20x lens, under low-angled light. When necessary, a stereomicroscope (Leica S8 APO 10-80x) was used for the identification and recording of bone surface modifications. Different taphonomical alterations (edaphic and biologic) were recorded based on the criteria identified in the scientific literature e.g., [24,37,100–105]. In order to identify the impact of taphonomical alterations, we recorded which part of the cortical surface was altered by each physico-chemical taphonomic modification, following [55]. More specifically,

for each alteration modifying the surface of the fragment, we attributed a scale code from 0 to 3. The code was 3 when the alteration covered the entire bone surface. The code was 2 if more than half of the entire bone surface was modified. If less than half the surface was affected, the code was 1. Finally, if no modification was observable to the naked eye, the code was 0. Taking into account all the alterations, we noted that cortical surfaces could be completely dissolved or concreted until they were illegible. Therefore, illegible remains were also counted for each assemblage. We compared the different surface modifications for the studied sites and layers.

We paid special attention to anthropogenic modifications and in particular to percussion marks. Burnt damage was also recorded [106]. We divided the cutmarks into two categories, according to the criteria established by [107]; incisions and scraping marks. We excluded the illegible remains from the percentages of the cut mark analysis. In the studied faunal assemblages, bone retouchees were recorded, and again, the illegible remains were excluded from the percentages. They were identified based on recent works setting out new criteria [41,108–110].

The long bone fragmentation of our assemblages was compared in terms of length: these classes are 0–25 mm, 26–50 mm, 51–75 mm, 75– 100 mm, 101–125 mm, >125 mm, bone part (complete epiphyses) and whole bones [111]. We measured the three dimensions (length, width, thickness) for each element. According to their size and circumference, we categorized the shaft fragments [112]: $l1 < 1/4 \leq l2 < 1/2 \leq l3 < 3/4 \leq l4 = 1$ (complete) and $0 < C1 < 0.5 \leq C2 < 1$; $C3 = 1$ (complete). Based on fracture features, we distinguished between dry bone fractures, recent brekage, gelifraction and green bone fractures [111,112].

The percussion marks were recorded following the terminology of [99]: crushing marks, notches, adhering flakes, pits and grooves. In this work, we did not include flakes because it was virtually impossible to locate them precisely on the long bone shaft. In order to identify preferentially impacted areas, each identified fragment was positioned in an area according to its long bone portion and side (cf. Figure 2 in [26]). Some bone fragments could be identified based on an anatomical particularity, such as the metatarsal gutter, but it was not possible to locate precise areas. In such cases, they were excluded from percussion mark distribution analyses.

The number of areas preserved by element varies according to the studied faunal assemblages. Therefore, we weighted the number of areas with percussion marks with the number of areas preserved for each area. The percussion mark ratio is calculated by: Number of areas with percussion marks / Number of preserved areas.

Experimental data

For the purposes of comparison, we included experimental data from three ~~major~~ experiments [33,49,50,113]. The main aim of these experiments was to record percussion mark location when novice experimenters broke bones to extract the yellow marrow. The analysis of percussion mark distribution along the long bone shaft provided data on intuitive patterns. These three experiments enable us to compare two different species ([33] and [113] for cow, and [49] for red deer) and the technique used to break the bones. Indeed, some experimenters used the hammerstone on anvil technique [33,49,50,113]; while the rest of them used the batting technique [33,113]. The analyses conducted to record percussion mark distribution are similar to those applied to the archaeological samples, presented above.

Data analysis

We used an alpha of 0.05 for all the tests. Spearman's rho was used to test the correlation between long bone density indexes and percussion mark frequency by portion to test whether the densest parts or the least dense zones were the most impacted. Bone density indexes were estimated by [37] and [114] LUNG for *Equus*, MUNG for *Cervus* and SUNG for *Ovis*. We also employed this bivariate test to cross the %MAU and the medullary cavity volume of ungulate long bones to explore human carcass transport strategies [115]. Finally, we tested the correlation between the numbers of percussion marks per element with its NISPa to determine whether the number of identified fragments affected the number of percussion marks recorded per element. The correlation coefficients and tests were computed using R software [116].

The chi-square test of independence was employed to verify if percussion mark locations were randomly distributed along the diaphysis or not. The chi-square test of conformity was used to verify if the observed distributions are similar to intuitive butchery distribution.

Multiple Correspondence Analysis (MCA) was used to investigate the relationship between the impacted and non-impacted bone portions (the three diaphyseal portions) and bone sides and to examine whether butchery patterns could be identified for specific sites and/or levels and bone elements. Therefore, the site, the level and the bone elements are displayed as illustrative variables. MCA was computed using the FactoMineR [117] library in R. When Cochran's rules were not respected, we used Fisher exact tests with R to test the independence between the locations of impacts on the long bone

shafts of each assemblage according to the different long bones. When necessary, pair wise multiple testing was performed. In such cases, the Holm correction of multiple testing was applied to the p-values.

In the MCA, our samples were compared with archaeological samples from Bolomor (IV, XVIIa, XVIIc) and Gran Dolina (TD-10-1 [upper part]) and with four experimental series [33,49,50,113],). The comparison of the results of [33] with two Middle Palaeolithic Spanish sites (MIS 5-9): Bolomor (levels IV, IX, XIIa and XIIc) and Gran Dolina (TD10-1), enables us to test the use of a standardized method for recovering marrow. Their results highlighted the presence of percussion mark patterns in Bolomor (level IV), indicating specific butchery traditions. On the contrary, in level 10-1 of Gran Dolina, the study did not demonstrate the presence of a tradition. We compared all the long bones. Metapodials were absent in the experimental samples, therefore, the comparison of these elements was only carried out for the archaeological samples.

Results

Taphonomic preservation of the bone samples

Bone surface preservation

The taphonomic alterations affect our various bone assemblages differently (Table 1), but they are quite similar within a same sequence. The vast majority of the long bone surfaces of Saint Marcel and Riparo Tagliente are legible, while one-third of the bone surfaces are illegible at Abri du Maras in both layers. This illegibility derives mainly from root etching dissolution, and in some cases prevents the identification of cut marks, bone retouchers or percussion pits. Notches are not usually affected by surface illegibility. Bone cracking is recorded on all the assemblages. Concretions affect bone surfaces to a lesser extent, in particular at Abri du Maras layer 4.2 and Riparo Tagliente.

Concerning animal-induced modifications, digested elements and carnivore or rodent tooth marks are either scarce or inexistent in all the sites. The site of Riparo Tagliente is the most affected, with only three elements having carnivore marks [26,55,56].

	sites	Saint Marcel cave				Riparo Tagliente				Abri du Maras					
	layer	g	%	h	%	35	%	37	%	4.1 MUNG	%	4.2 LUNG	%	4.2 MUNG	%
Cracking	f0	146	56.59%	198	65.35%	88	63.77%	104	59.09%	452	60.92%	20	9.85%	75	20.44%
	f1	112	43.41%	105	34.65%	31	22.46%	53	30.11%	247	33.29%	59	29.06%	160	43.60%
	f2	50	19.38%	69	22.77%	14	10.14%	12	6.82%	40	5.39%	65	32.02%	93	25.34%
	f3	4	1.55%	27	8.91%	5	3.62%	7	3.98%	3	0.4%	59	29.1%	39	10.63%

Concretion	c0	226	87.60%	241	79.54%	112	81.16%	110	62.5%	646	87.06%	17	8.37%	63	17.17%
	c1	30	11.63%	47	15.51%	18	13.04%	56	31.82%	85	11.46%	157	77.34%	281	76.57%
	c2	2	0.78%	15	4.95%	6	4.35%	8	4.55%	11	1.48%	29	14.29%	23	6.27%
	c3	0	0%	0	0%	2	1.45%	2	1.14%	0	0%	0	0%	0	0.00%
Dissolution	s0	185	71.71%	222	73.27%	26	18.84%	59	33.52%	18	2.43%	2	0.99%	6	1.63%
	s1	58	22.48%	68	22.44%	110	79.71%	114	64.77%	281	37.87%	26	12.81%	93	25.34%
	s2	23	8.91%	13	4.29%	2	1.45%	3	1.7%	267	35.98%	75	36.95%	144	39.24%
	s3	0	0%	0	0%	0	0%	0	0%	166	22.37%	100	49.26%	124	33.79%
Blunting	b0	208	80.62%	189	62.38%	129	93.48%	135	76.7%	688	92.72%	98	48.28%	232	63.22%
	b1	44	17.05%	103	33.99%	9	6.52%	3	1.7%	51	6.87%	79	38.92%	124	33.79%
	b2	6	2.33%	11	3.63%	0	0%	0	0%	3	0.4%	24	11.8%	11	3.00%
	b3	0	0.00%	0	0.00%	0	0%	0	0%	0	0%	2	1%	0	0.00%
Desquamation	d0	207	80.23%	251	82.84%	72	52.17%	51	28.98%	206	27.76%	40	19.70%	91	24.80%
	d1	35	13.57%	44	14.52%	95	68.84%	69	39.20%	170	22.91%	108	53.20%	151	41.14%
	d2	14	5.43%	8	2.64%	8	5.80%	18	10.23%	200	26.95%	45	22.17%	100	27.25%
	d3	2	0.78%	0	0%	1	0.72%	0	0.00%	166	22.37%	10	4.93%	25	6.81%
Root etching	r0	0	0%	0	0%	35	25.36%	81	46.02%	29	3.91%	4	1.97%	13	3.54%
	r1	0	0%	0	0%	102	73.91%	94	53.41%	246	33.15%	33	16.26%	96	26.16%
	r2	0	0%	0	0%	1	0.72%	1	0.57%	270	36.39%	80	39.41%	160	43.60%
	r3	0	0%	0	0%	0	0%	0	0%	197	26.55%	86	42.36%	98	26.70%
Black coloring		0	0%	0	0%	116	84.06%	159	90.34%	590	79.51%	183	90.15%	331	90.19%
Orange coloring		0	0%	0	0%	80	57.97%	119	67.61%	365	49.19%	82	40.39%	139	37.87%
Illegible		2	0.78%	1	0.33%	5	3.62%	4	2.27%	180	24.26%	87	42.86%	123	33.51%
**Total**		**258**	**100%**	**303**	**100%**	**138**	**100%**	**176**	**100%**	**742**	**100%**	**203**	**100%**	**367**	**100%**

Table 1: Bone surface alterations (NR and τ) with for each of ~~them~~ the stages (3 to 0) of surface modifications for each level of each site, Saint Marcel cave layer g and h; Riparo Tagliente layer 35 and 37); Abri du Maras layer 4.1, 4.2 LUNG and 4.2 MUNG

.

Long bone element distribution and differential bone preservation

The ~~number of each~~ long bone ~~element~~ varies according to the layers and ungulate class-size. The %MAU of the long bones of Riparo Tagliente is the highest since almost all the elements have a ratio superior to 50% (Table 2 and S1 Figure). Tibias are the most abundant long bones for all the assemblages, except in layer 4.1 (Abri du Maras), where metatarsals are the most frequent (Figure 2). Radio-ulnas, with the exception of AM-4.2LUNG assemblage, are also numerous.

The %MAU of each long bone ~~element~~ and the marrow cavity volume (ml) are not significantly correlated (Spearman correlation: df = 5; p-value > 0.05). On the contrary, the %MAU and the bone density are significantly and positively correlated for: Abri du Maras, series 4.1 and 4.2MUNG; Saint-Marcel Cave g and h (Spearman correlation: df = 25; p-value < 0.01; $r_s < 0.01$) and Riparo Tagliente layer 35 (Spearman correlation: df = 25; p-value = 0.01; $r_s = 0.45$). No significant correlation is noted for layer 37 of Riparo Tagliente and the 4.2LUNG assemblage of Abri du Maras (Spearman correlation: df = 25; p-value > 0.05).

Figure 2: % MAU of the long bone elements for each series: A- Saint Marcel g, B- Saint Marcel h, C- Riparo Tagliente 35, D- Riparo Tagliente 37, E- Abri du Maras 4.1, F & G- Abri du Maras 4.2.

Sites	Element	NISPa	% NISPa	Right	Left	juv	NME	% NME	MAU	% MAU	Max (mm)	Min (mm)	Mean (mm)	SD
Saint-Marcel Layer g MUNG	Humerus	18	7%	12	6	2	9	8%	4.5	24%	119	39	69.89	20.06
	Radio-ulna	41	16%	21	14	1	24	20%	12	65%	157	30	88.68	28.59
	Metacarpal	31	12%	7	9	0	13	11%	6.5	35%	143	33	71.81	29.69
	Femur	32	12%	12	10	2	16	13%	8	43%	149	36	88.88	28.07
	Tibia	64	25%	18	35	0	37	31%	18.5	100%	181	38	92.82	29.89
	Metatarsal	47	18%	6	12	1	18	15%	9	49%	156	32	76.71	27.52
	Metapodial	25	10%	3	3	1	3	3%	1.5	8%	159	9	77.56	28.82
	**Total**	**258**		**79**	**89**	**7**	**120**		**60**		**181**	**9**	**83.09**	**29.34**
Saint-Marcel Layer h MUNG	Humerus	30	10%	12	14	1	17	13%	8.5	52%	138	39	73.06	23
	Radio-ulna	43	14%	19	19	1	17	13%	8.5	52%	137	34	73.58	25.89
	Metacarpal	45	15%	7	7	2	13	10%	6.5	39%	128	30	72.02	22.42
	Femur	36	12%	14	14	0	18	14%	9	55%	154	29	82.31	29.43
	Tibia	60	20%	18	31	6	33	25%	16.5	100%	157	43	88.95	25.18
	Metatarsal	76	25%	19	19	0	33	25%	16.5	100%	150	29	80.66	29.17
	Metapodial	13	4%	0	0	2	1	1%	0.5	3%	121	22	62.38	21.18
	**Total**	**303**		**89**	**104**	**12**	**132**		**66**		**157**	**22**	**79.48**	**27.01**
Riparo Tagliente Layer 35 SUNG	Humerus	18	13%	6	5	0	11	13%	5.5	69%	56	26	40.5	12.35
	Radio-ulna	5	4%	4	1	0	9	10%	4.5	56%	74	18	51.63	18.42
	Metacarpal	32	23%	4	3	2	16	19%	8	100%	88	14	39.31	13.58
	Femur	15	11%	1	2	0	11	13%	5.5	69%	77	25	38.72	11.26
	Tibia	20	14%	9	7	0	16	19%	8	100%	89	25	57.48	19.2
	Metatarsal	39	28%	1	2	2	15	17%	7.5	94%	85	14	37.46	17.2
	Metapodial	9	7%			0	8	9%	4	50%	73	15	40.67	12.55

	**Total**	**138**		**25**	**20**	**4**	**86**		**43**		**89**	**14**	**42.86**	**16.64**
Riparo Tagliente Layer 37 SUNG	Humerus	18	10%	9	4	0	9	17%	4.5	82%	65	19	59.6	9.65
	Radio-ulna	13	7%	9	2	1	6	12%	3	55%	75	31	51.79	13.76
	Metacarpal	33	19%	4	3	0	9	17%	4.5	82%	75	14	42.38	14.21
	Femur	22	13%	4	10	0	5	10%	2.5	45%	55	22	42.86	13.75
	Tibia	39	22%	15	16	2	11	21%	5.5	100%	99	29	50.23	16.24
	Metatarsal	30	17%	2	2	1	10	19%	5	91%	95	14	41.38	15.99
	Metapodial	21	12%			1	2	4%	1	18%	64	19	41.67	16.98
	**Total**	**176**		**43**	**37**	**5**	**52**		**26**		**99**	**14**	**44.45**	**15.21**
Abri du Maras Layer 4.1 MUNG	Humerus	97	13%	32	40	0	34	18%	17	55%	123	23	54.89	17.91
	Radio-ulna	123	17%	34	32	1	41	21%	20.5	66%	193	22	59.64	26.36
	Metacarpal	58	8%	13	14	2	19	10%	9.5	31%	181	16	60.25	34.07
	Femur	45	6%	8	12	0	17	9%	8.5	27%	110	28	61.44	21.66
	Tibia	184	25%	53	58	3	41	21%	20.5	66%	166	16	63.52	24.6
	Metatarsal	167	23%	24	33	2	62	32%	31	100%	150	16	56.91	25.07
	Metapodial	68	9%	1	1	3	11	6%	5.5	18%	101	16	45.19	20.92
	**Total**	**742**		**165**	**190**	**11**	**225**		**112.5**		**193**	**16**	**57.41**	**25.1**
Abri du Maras Layer 4.2 MUNG	Humerus	41	31433%	13	21	0	20	12%	10	44%	130	32	62.66	21.92
	Radio-ulna	79	60567%	16	11	0	43	33618%	21.5	96%	177	23	62.09	31.75
	Metacarpal	23	17633%	1	10	0	8	6255%	4	18%	171	21	71.09	27.6
	Femur	32	24533%	10	10	0	15	11727%	7.5	33%	105	26	72.41	32.54
	Tibia	95	72833%	32	40	2	45	35182%	22.5	100%	161	34	76.97	36.1
	Metatarsal	72	55200%	10	7	0	29	22673%	14.5	64%	219	23	59.77	23.89
	Metapodial	25	19167%	1	0	1	8	6255%	4	18%	105	35	60.16	18.64
	**Total**	**367**		**83**	**99**	**3**	**168**		**84**		**219**	**21**	**66.9**	**29.88**
Abri du Maras Layer 4.2 LUNG	Humerus	32	16%	11	11	0	16	16%	8	35%	147	48	92.06	23.92
	Radio-ulna	29	28356%	3	3	0	13	7511%	6.5	28%	240	41	100.59	46.03
	Metacarpal	6	5867%	0	4	0	4	2311%	2	9%	245	52	134	28.99
	Femur	29	28356%	7	6	0	14	8089%	7	30%	149	27	95.93	33.85
	Tibia	93	90933%	25	35	1	46	26578%	23	100%	309	28	101.67	49.13
	Metatarsal	8	7822%	1	1	0	3	1733%	1.5	7%	132	39	77.88	34.25
	Metapodial	6	5867%	0	0	0	2	1156%	1	4%	114	26	78	39.88
	**Total**	**203**		**47**	**60**	**1**	**98**		**49**		**309**	**26**	**98.52**	**42.49**
	Total	2187	0	531	599	43	881	0	440.5	0	309	9	67.53	9.14758412

Table 2: Number of anatomically Identified Specimen (NISPa), Left (L) and Right (R) sides, Number of Remains belonging to juveniles (juv), Minimum Number of Element (MNE), Minimum Animal Unit (MAU), maximal length (Max), minimal length (Min) mean size of long bones (Mean) and standard deviation (SD).

Long bone breakage

All the long bones ~~series~~ are highly fragmented (S4-9 Figures). No complete long bones were found at Riparo Tagliente and Saint Marcel. At Abri du Maras, we only identified metapodials: one complete reindeer metacarpal in layer 4.2, and three reindeer metapodials undetermined and a horse metacarpal in layer 4.1 [26]. No complete bone circumferences were preserved. For all the series, the majority of the remains have a c1 circumference and a l1/l2 length (S2 Figure).

Shaft fragments are very well represented for all the studied series (Table 3). The epiphysis-diaphysis ratio is 0.17 (323/1917). The scarcity of epiphyses could be explained by numerous factors, such as differential conservation, anthropogenic breakage to recover bone grease or the use of bone cancellous as fuel [118–121].

	Saint Marcel cave		Riparo Tagliente		Abri du Maras		
	g	h	35	37	4.1 MUNG	4.2 LUNG	4.2 MUNG
Epiphysis	25	41	13	24	162	27	31
Diaphysis	282	273	109	169	888	196	282
**Epiphysis/Diaphysis**	**0.089**	**0.150**	**0.119**	**0.142**	**0.182**	**0.138**	**0.110**

Table 3: Number of epiphysis and diaphysis long bone portions and ratios of epiphysis on diaphysis (E/D).

Regarding the dimensions of bone fragments, most of the remains are between 25 mm and 100 mm long (Tables 2 and 4, S3 Figure). In addition, we note a differential fragmentation rate according to ungulate class size. None of the 4.2LUNG remains from Abri du Maras is smaller than 25 mm in length, whereas the SUNG remains from Riparo Tagliente are the most abundant for this length interval. The SUNG remains from Riparo Tagliente 35 and 37 are never longer than 100 mm while almost 50% (89/203) of the remains from Abri du Maras 4.2LUNG are greater than 100 mm long.

Sites	Saint Marcel cave				Riparo Tagliente				Abri du Maras					
Layer	g		h		35		37		4.1MUNG		4.2LUNG		4.2MUNG	
	NISP	%	NISP	%	NISP	%	NISP	%	NISP	%	NISP	%	NISP	%
**Green bone fracture**	252	97.67%	283	93.4%	136	98.55%	163	92.61%	682	91.91%	192	94.58%	358	97.55%
**Dry bone fracture**	71	27.52%	152	50.17%	34	24.64%	20	11.36%	222	29.92%	53	26.11%	75	20.44%
**Und**	2	0.78%	4	1.32%	9	6.52%	16	9.09%	88	11.86%	45	22.17%	61	16.62%
**Gelifraction**	0	0%	0	0%	5	3.62%	0	0%	17	2.29%	8	3.94%	38	10.35%
**Nr burnt**	40	15.50%	107	35.31%	7	5.07%	4	2.27%	10	1.35%	1	0.49%	1	0.27%
**Cutmarks**	175	68.36%	166	54.97%	64	48.12%	91	52.9%	211	37.54%	59	50.86%	83	34.02%
**Percussion marks**	146	56.59%	136	44.88%	56	40.58%	69	39.2%	197	26.55%	77	37.93%	112	30.52%
**Retoucher**	23	8.98%	37	12.25%	8	6.0%	11	6.40%	0	0%	5	4%	6	2.46%
**Total**	**258**	**100%**	**303**	**100%**	**138**	**100%**	**176**	**100%**	**742**	**100%**	**203**	**100%**	**367**	**100%**

Table 4: Frost, green, dry and indeterminate long bone breakage (NISP and τ); measurement intervals along the length of the long bone (NR and τ); anthropogenic marks (NR and τ).

Fracture profiles are mainly curved/V-shaped with oblique angles and smooth edges. Thus, green bone fractures are the most numerous, affecting more than 90% of the number of the studied remains for each layer. Dry bone fractures are recorded on approximatively 25% of all samples (Table 4). The number of indeterminate fractures varies from one site to another from 1 to 22%. Gelifraction is a minor factor in the fragmentation of bone assemblages, and only appears at Abri du Maras and Riparo Tagliente layer 35.

Anthropogenic modifications

Cut marks are the best represented anthropogenic marks in our assemblages, followed by percussion marks (Table 4). We recorded a lower cut mark rates in layers 4.1 and 4.2 of Abri du Maras than in the other sites. In most of the layers of Riparo Tagliente and Saint-Marcel Cave, we identified cut marks on more than 50% of the identified fragments.

For the Abri du Maras and Riparo Tagliente samples, the number of burnt remains is very low (< 5 %). However, in these two sites, we observe numerous indeterminate burnt splinters [97]. At Saint-Marcel, burnt elements are more abundant, with percentages between 15 to 35 % among our samples (Table 4).

With the exception of layer 4.1 of Abri du Maras, where no retouchers were identified regarding our sample, in the other studied samples their number varies according to the layers (Table 4). Saint-Marcel Cave is the site with the most bone retouchers (between 9 and 12%). At Riparo Tagliente, despite the predominance of small ungulates, percentages are around 6%.

Percussion marks analysis

Percussion marks type

Notches are the predominant percussion marks for the studied samples, followed by pits. Adhering flakes and grooves are the least numerous. Crushing marks are totally absent from our assemblages (Table 5).

In the Abri du Maras assemblages, almost all the percussion marks are notches, but we also observed pits of ovoid morphology and adhering flakes. A slightly higher number of pits and adhering flakes was identified for 4.2LUNG layer than for layer 4.1. No grooves were observed (Table 5).

In the Saint-Marcel series (Table 5), around two-thirds of the percussion marks are notches and around a quarter are pits. The rate of adhering flakes and grooves is lower, at around 10%. Most of the notches, in both layers, are conchoidal and a minority present an internal triangular pit or groove. Triangular percussion pits are more numerous in both layers.

In the Riparo Tagliente layers, grooves and adhering flakes constitute around 1% of all percussion marks. Again, notches are the most numerous type of marks identified. They are all conchoidal whereas a minority identified in layer 35 present an internal triangular pit or a groove. Pits are the second most represented type of mark, generally with an ovoid morphology (Table 5).

Site	Saint Marcel cave				Riparo Tagliente				Abri du Maras					
Layer	**g**		**h**		**35**		**37**		**4.1MUNG**		**4.2LUNG**		**4.2MUNG**	
	**NISP**	**%**	**NISP**	**%**	**NISP**	**%**	**NISP**	**%**	**NISP**	**%**	**NISP**	**%**	**NISP**	**%**

Percussion marks	253		242		88		107		219		97		148	
**Notch**	**171**	**67.6%**	**151**	**62.4%**	**58**	**65.9%**	**90**	**84.1%**	**218**	**99.5%**	**93**	**95.9%**	**144**	**97.3%**
triangular	21	12.3%	25	16.6%	6	10.3%	0	0%	0	0%	0	0%	0	0%
ovoide	150	87.7%	126	83.4%	52	89.7%	90	100%	0	0%	94	101%	144	100%
**Pit**	**59**	**23.3%**	**69**	**28.5%**	**29**	**33.0%**	**16**	**15.0%**	**1**	**0.5%**	**4**	**4.1%**	**2**	**1.4%**
triangular	32	54.2%	57	82.6%	9	31.0%	7	43.8%	0	0%	0	0%	0	0%
ovoide	27	45.8%	12	17.4%	20	69.0%	9	56.3%	1	100%	4	100%	2	100%
**Groove**	**2**	**0.8%**	**5**	**2.1%**	**1**	**1.1%**	**0**	**0%**	**0**	**0%**	**0**	**0%**	**0**	**0%**
**Crushing marks**	**0**	**0%**	**3**	**1.2%**	**0**	**0%**	**0**	**0%**	**0**	**0%**	**0**	**0%**	**0**	**0%**
**Adhering flake**	**21**	**8.3%**	**17**	**7%**	**0**	**0%**	**1**	**0.9%**	**0**	**0%**	**10**	**10.3%**	**2**	**1.4%**
Humerus	23	9.1%	22	9.1%	10	11.4%	25	23.4%	44	20.1%	22	22.7%	19	12.8%
Radio-ulna	40	15.8%	23	9.5%	3	3.4%	3	2.8%	24	11.0%	11	11.3%	26	17.6%
Metacarpal	61	24.1%	47	19.4%	19	21.6%	15	14%	14	6.4%	3	3.1%	8	5.4%
Femur	26	10.3%	26	10.7%	13	14.8%	14	13.1%	21	9.6%	26	26.8%	15	10.1%
Tibia	55	21.7%	38	15.7%	12	13.6%	25	23.4%	77	35.2%	34	35.1%	56	37.8%
Metatarsal	31	12.3%	78	32.2%	29	33%	22	20.6%	37	16.9%	0	0%	20	13.5%
Metapodial	17	6.7%	8	3.3%	2	2.3%	3	2.8%	2	0.9%	1	1.0%	4	2.7%
**Total**	**253**		**242**		**88**		**107**		**219**		**97**		**148**	

Table 5: Number of percussion marks by layer of the studied sites, by type of percussion mark and by element

The number of percussion marks by elements

At Abri du Maras, the three faunal samples show a relatively similar distribution of percussion marks per element (Table 5). One third of the percussion marks are located on tibias, while only a few percussion marks were identified on metacarpal remains. More specifically, for layer 4.2LUNG, none of the metatarsal remains present percussion marks. Percussion marks are equally represented for the other long bones.

Similarly, at Saint-Marcel Cave, percussion mark distribution is equivalent per element for both levels. Tibias and metapodials are the most impacted fragments.

For the Riparo Tagliente layers, tibias and metapodials are also the bone elements with the most marked areas.

The number of percussion marks and NISPa by element are significantly positively correlated in Abri du Maras 4.2LUNG (Spearman correlation: p-value = 0.032, r_s = 0.82), Abri du Maras 4.2MUNG (Spearman correlation: df = 6, p-value < 0.01, r_s = 0.96) and Saint Marcel cave level h (Spearman correlation: df = 6, p-value = 0.007, rs = 0.93) and Riparo Tagliente 35 (Spearman correlation: df = 6, p-value = 0.012, r_s = 0.86). In the other assemblages, they were not significantly correlated (Abri du Maras, 4.1; Saint Marcel Cave, g and Riparo Tagliente, 37: Spearman correlation: df = 6, p-value > 0.05). In terms of the whole assemblage of each site, only Saint Marcel Cave showed a positively significant correlation between these variables, while the Abri du Maras and Riparo Tagliente faunal assemblages did not show a correlation (Spearman correlation: df = 6, p-values > 0.05).

The percussion marks frequency by bone areas

The frequencies of the impacted portions and long bone density indexes are not significantly correlated for each series (Spearman correlation: df = 25; p-value > 0.05). None of the studied assemblages or long bones show a systematic distribution of percussion marks, i.e., only one or two struck areas (Figures 3, 4, 5, 6, 7 and 8). The number of areas with at least one percussion mark varied between two and 14 areas per bone element. Thus, in order to identify tendencies, we took into account frequencies equal to or higher than 0.33 and counted their occurrence per element, so that we could carry out inter- and intra-site comparisons. The lateral side of the medial portion (p3l) of the humerus shows higher proportions in five faunal assemblages (layers g, 35, 37, 4.1 and 4.2MUNG, 71.4%) (Figure 3). We record high frequencies for the femur in five series on the proximal portion (p2l) of the lateral side (layers g, h, 35, 4.1 and 4.2LUNG; 71.4%) (Figure 6). For the radio-ulna, the most impacted area is the anterior side of the distal portion (p4a)

(layers g, h, 4.1 and 4.2LUNG; 42.9%) (Figure 4). For the metacarpal, we identified two areas with high frequencies of impact traces: the lateral side of the proximal portion (p2l) and the medial side of the medial portion (p3m), for three of our samples (layers g, h, and 4.1 and layers g, 4.1 and 4.2MUNG; 42.9%) (Figure 5). On the metatarsal, five areas were identified. Two tibia series do not show high frequencies (Saint-Marcel Cave layer g and Abri du Maras layer 4.2LUNG) (Figure 8). For the others, only 28.5% show a tendency with higher frequencies. The areas with higher frequency tendencies are always located on the diaphysis. Within the same site, the most impacted areas do not systematically display similar locations.

Figure 3: Frequencies of number of percussion marks (% and Number of area with percussion mark(s) / Number of area preserved) by portion on humerus from Saint Marcel cave level g (A) and level h (B), Riparo Tagliente level 35 (C) and level 37 (D), and Abri du Maras level 4.1 (E), level 4.2 LUNG (F), level 4.2 MUNG. The absence of portion in each assemblage is grey.

Figure 4: Frequencies of number of percussion marks (% and Number of area with percussion mark(s) / Number of area preserved) by portion on radio-ulna from Saint Marcel cave level g (A) and level h (B), Riparo Tagliente level 35 (C) and level 37 (D), and Abri du Maras level 4.1 (E), level 4.2 LUNG (F), level 4.2 MUNG. The absence of portion in each assemblage is grey.

Figure 5 Frequencies of number of percussion marks (% and Number of area with percussion mark(s) / Number of area preserved) by portion on metacarpal from Saint Marcel cave level g (A) and level h (B), Riparo Tagliente level 35 (C) and level 37 (D), and Abri du Maras level 4.1 (E), level 4.2 LUNG (F), level 4.2 MUNG. The absence of portion in each assemblage is grey.

Figure 6 : Frequencies of number of percussion marks (% and Number of area with percussion mark(s) / Number of area preserved) by portion on femur from Saint Marcel cave level g (A) and level h (B), Riparo Tagliente level 35 (C) and level 37 (D), and Abri du Maras level 4.1 (E), level 4.2 LUNG (F), level 4.2 MUNG. The absence of portion in each assemblage is grey.

Figure 7 : Frequencies of number of percussion marks (% and Number of area with percussion mark(s) / Number of area preserved) by portion on humerus from Saint Marcel cave level g (A) and level h (B), Riparo Tagliente level 35 (C) and level 37 (D), and Abri du Maras level 4.1 (E), level 4.2 LUNG (F), level 4.2 MUNG. The absence of portion in each assemblage is grey.

Figure 8: Frequencies of number of percussion marks by portion on metatarsal of each sample (% and Number of area with percussion mark(s) / Number of area preserved); Saint Marcel cave level g (A), level h (B); Riparo Tagliente level 35 (C), level 37 (D); Abri du Maras level 4.1 (E), level 4.2 LUNG (F), level 4.2 MUNG. The absence of portion in each assemblage is grey.

Non-random and counter-intuitive distribution of percussion marks

We compare our results with those from [33] two Middle Palaeolithic Spanish sites (MIS 5-9): Bolomor (levels IV, IX, XIIa and XIIc) and Gran Dolina (TD10-1). For the experimental data, we used the intuitive pattern presented in [113] (Figure 9, 10 and 11).

The hypothesis tested on the archaeological samples, including comparative data, is the presence of a random pattern of percussion mark distribution. When data are quantitatively limited, we group the series from the same site together. The results show that at Bolomor, percussion mark areas are not randomly distributed, in contrast to Gran Dolina with the previously published chi-square. Regarding our studied sites, Riparo Tagliente presents a probable random distribution of the marked areas (Chi-square: p-value > 0.05). Nonetheless, in the site of Abri du Maras, four long bones: humerus (Chi-square: p-value < 0.001, $\chi^2 = 40.86$), radio-ulna (Chi-square: p-value $= 0.002$, $\chi^2 = 29.53$), femur (Chi-square: p-value < 0.001, $\chi^2 = 21.11$) and metatarsal (Chi-square: p-value $= 0.001$, $\chi^2 = 31.08$) show that bone areas are not equally impacted. At Saint-Marcel Cave, three long bones: tibia (Chi-square: p-value $= 0.031$, $\chi^2 = 21.16$), metacarpal (Chi-square: p-value $= 0.019$, $\chi^2 = 22.76$) and metatarsal (Chi-square: p-value < 0.001, $\chi^2 = 40.06$) yield similar test results.

Figure 9: Number of areas with percussion marks for each archaeological site and for the intuitive experiment model for humerus and radio-ulnas. Portion 2 (P2): proximal diaphysis; Portion 3 (P3): medial diaphysis; Portion 4 (P4): distal diaphysis; sides: anterior (a), lateral (l), posterior (p) and medial (m).

Figure 10: Number of areas with percussion marks for each archaeological site and for the intuitive experiment model for femurs and tibias. Portion 2 (P2): proximal diaphysis; Portion 3 (P3): medial diaphysis; Portion 4 (P4): distal diaphysis; sides: anterior (a), lateral (l), posterior (p) and medial (m).

Figure 11: Number of areas with percussion marks for each archaeological site for metacarpals and metatarsals. Portion 2 (P2): proximal diaphysis; Portion 3 (P3): medial diaphysis; Portion 4 (P4): distal diaphysis; sides: anterior (a), lateral (l), posterior (p) and medial (m).

In order to develop the analysis at an intra-site scale, we need to test each assemblage independently. For the sake of representativeness, we only considered series with at least 15 areas with percussion marks (Figure 9, 10 and 11). Except the radio-ulna and the metatarsal of Abri du Maras 4.2LUNG, all the elements from the Abri du Maras and Saint-Marcel Cave levels were eligible to be tested. The three Abri du Maras assemblages show differences. The level 4.1 sample presents a non-random distribution of the impacted areas for the humerus (Chi-square: p-value < 0.001, $\chi^2 = 29.11$), the femur (Chi-square: p-value $= 0.002$, $\chi^2 = 32$) and the metatarsal (Chi-square: p-value < 0.001, $\chi^2 = 38$). In level 4.2, the radio-ulnas of the 4.2MUNG assemblage (Chi-square: p-value $= 0.001$, $\chi^2 = 30.74$) and the humeri of the 4.2LUNG sample (Chi-square: p-value $= 0.003$, $\chi^2 = 21.2$) present proportions of bone areas unequally marked by percussion. The Saint-Marcel Cave series shows differences depending on the element. The metatarsal for both series presents a non-random distribution (Chi-square: level g: p-value $= 0.02$, $\chi^2 = 22$ and level h: p-value < 0.001, $\chi^2 = 35.73$). In layer g, percussion marks are randomly distributed on the metacarpal (Chi-square: p-value $= 0.01$, $\chi^2 = 24.64$), whereas this is not the case in level h. Finally, bone areas are equally marked for the tibias from each level.

If we only consider the series with non-random distribution, it is possible to discuss butchery traditions or intuitive patterns. Some works highlight the existence of intuitive patterns in percussion mark distribution for marrow recovery, based on experiments involving non-trained experimenters [50,113,122]. Using the results of this experiment and chi-square analysis, we compared whether the non-random distribution of percussion marks is statistically different or not from intuitive pattern distribution.

The Bolomor site reveals a different distribution from the intuitive one (Chi-square: p-value < 0.01, humerus: $\chi^2 = 158.58$; femur: $\chi^2 = 39.52$; tibia: $\chi^2 = 39.57$), except for the radio-ulna (Chi-square: p-value > 0.05, $\chi^2 = 19.3$). For radio-ulnas, the same results were noted as for radio-ulnas[113]. In the Abri du Maras and Saint-Marcel Cave series, only the elements with non-random percussion mark distribution were tested with intuitive patterns. All of them show a different distribution to the intuitive pattern (Chi-square: Maras: humerus, p-value < 0.001, $\chi^2 = 51.86$; radius, p-value $= 0.033$, $\chi^2 = 21.01$ and femur, p-value $= 0.006$, $\chi^2 = 26.1$; Saint-Marcel: tibia, p-value $= 0.008$, $\chi^2 = 25.53$). Moreover, at Abri du Maras, the elements with results showing unequal marked areas also display a different distribution from an intuitive pattern (Chi-square: Maras, 4.1: humerus, p-value < 0.001, $\chi^2 = 38.43$ and femur, p-value < 0.001, $\chi^2 = 38.82$; 4.2MUNG: radius, p-value < 0.001, $\chi^2 = 22.69$ and 4.2LUNG: humerus, p-value $= 0.002$, $\chi^2 = 21.96$).

Significant differences between assemblages

The MCA includes both the bone areas with and without percussion marks (S10 Figure). The first axis, accounting for around 17% of the total variance, is related to the impact traces presence, with impacted areas on the right of the chart and non-impacted areas on the left. Predictably, the experiment yielded more preserved and impacted areas than the archaeological assemblages did. Also, the most impacted portion and side seem to be respectively portion 3 and the medial side. In the controlled experiment, all the remains were kept and conserved, whereas several remains are missing from the archaeological series due to differential conservation or problems of identification (S2 Table) (Figures 9, 10 and 11).

In order to examine in more details the relation between the portions and the sides bearing percussion marks depending on the sites and bone elements, we excluded areas without percussion marks from the analysis. The location of percussion marks on the portions and sides are used to build the axes of the MCA while the sites and their levels are displayed as illustrative variables. Separate MCA are computed for each bone element (S10 and S11 Figures). In most of the plots, most of the sites are very close to the centre of the graph, showing that it is difficult to differentiate the sites and the experiments based on these variables. For the humerus plot, the Abri du Maras seems to be different to the intuitive experiment, but the series are close to the other sites. On the radio-ulna graph, the Abri du Maras and Saint-Marcel series show some differences, but they are encompassed in the ellipse of Gran Dolina and Riparo Tagliente. Regarding the femur, the ellipses of the Saint-Marcel, Abri du Maras and Riparo Tagliente assemblages intersect but they present differences. Nevertheless, the series of Saint-Marcel and Abri du Maras are included in the ellipse of Gran Dolina. However, the site of Bolomor is always far removed from the other experimental and archaeological groups.

Regarding the MCA including experimental data, we observe that the batting experiment is distant from the other experiments [33,123]. The most impacted areas are on radio-ulna, the portion 2 and posterior side, on femur the portion 2 and anterior side and on tibia with the more impacted area the portion 2 and lateral side (lateral side has a low $\cos^2$) (Figure 10 and 11). The other experiment of [33] is quite distant from the center (humerus, radio-ulna and femur), but less than the batting series. The other experimental assemblages are grouped towards the plot center, showing that the considered variables (the portion or the side) are not discriminatory. Besides, the sample from Riparo Tagliente level 35 is distant from the other archaeological assemblages for the femur and humerus.

Regarding the metapodials, the absence of experiments seems to influence plot distribution (Figure 14). For the metapodial graphs, Bolomor shows a certain proximity to the Gran Dolina assemblage

with more impact traces on portion 3 and the anterior side (but the anterior side has a low cos²) on the metacarpal and on portion 3 and the lateral side on the metatarsal. Bolomor is set apart from our series.

In order to test if the tendencies observed in the MCA are statistically significant (in other words, if they could be due to chance or not), we used the Fisher exact test. The results display significant differences for the area with impact distribution on the metatarsal between Abri du Maras and Saint-Marcel (p3a/p3l, p-value < 0.001 and p3a/p4l, p-value < 0.001).

Figure 12: MCA of the areas with percussion marks by bone portion and side for humerus (A-B) and radio-ulnas (C-D). Sites are included as illustrative variable only. Cos² are displayed following a color gradient.

Figure 13: MCA of the areas with percussion marks by bone portion and side for femur (A-B) and tibia (C-D). Sites are included as illustrative variable only. Cos² are displayed following a color gradient

Figure 14: MCA of the areas with percussion marks by bone portion and side for metacarpal (A-B) and tibia (C-D). Sites are included as illustrative variable only. Cos² are displayed following a color gradient

Discussion

Through the analysis of seven faunal assemblages from three South European Middle Palaeolithic sites: Abri du Maras, Saint Marcel and Riparo Tagliente, we conducted a large comparative analysis of the percussion marks distribution on long bones. In all these series, we observed distinct and homogeneous long bone fragmentation. We also identified a high number of green bone fractures and numerous percussion marks of different types (notches, adhering flakes, pits and grooves) as the result of anthropogenic breakage. The sites record intensive human occupation and the number and diversity of the percussion marks attest to systematic long bone breakage for marrow removal.

Percussion marks are never systematically located on the same specific bone area in the different layers and sites. However, some tendencies emerge and the heterogeneous percussion mark distribution highlights some more marked areas than others do. These trends differ according to the elements and faunal series. Our results show that the humeri and the femurs of layer 4.1 of Abri du Maras present a non-random percussion mark distribution, which is different from that obtained in an intuitive context. Likewise, the analyses demonstrate a similarly non-random and counter-intuitive pattern for the percussion mark distribution of the ~~series~~ 4.2LUNG (large-sized ungulate series) humeri and the 4.2 MUNG

(middle-sized ungulate series) tibias of Abri du Maras, and also of the Saint-Marcel Cave radio-ulnas. Thus, this particular distribution of percussion marks shows the probable existence of butchery traditions at Abri du Maras layers 4.1, 4.2, and Saint-Marcel layers g, and h. At Riparo Tagliente, this is not obvious, due perhaps to statistical biases. However, it was not possible to discriminate sites on the basis of percussion mark distribution, except for the metatarsals from Saint-Marcel and Abri du Maras, indicating a statistically significant difference between them.

Taphonomic biases in the identification of percussion marks

In order to analyse ~~deeper~~ the distribution of percussion marks and its meaning, it is necessary to estimate the impact of the different taphonomic factors on faunal assemblages.

Taphonomic differences clearly exist between our sites. The percussion marks ratios from both layers at Abri du Maras are lower than at Riparo Tagliente and Saint Marcel. This is partly due to the greater illegibility of the Abri du Maras bone surfaces (root etching dissolution and desquamation). Indeed, the greatest difference between the series is related to the ratio of pits. While the pits are the second most frequent traces at Saint Marcel and Riparo Tagliente, at Abri du Maras, they are scarce or absent. Pits only alter the superficial cortical surface, whereas notches and adhering flakes penetrate the whole thickness and reach the medullary cavity [99,124]. Thus, the degree of bone surface preservation may partly explain the limited number of pits in this case. Concretions may also sometimes partially or completely cover the surfaces. The same is true for abrasion alterations, such as polishing. However, oxide coloration, as well as fire surface alterations, do not seem to affect percussion marks.

The degree of post-depositional bone fragmentation should also be considered in the percussion marks analysis. In our series, breakage was mainly perimortem, as evidenced by the high proportion of green bone fractures. However, some dry bone fractures were also observed, sometimes related to green bone fractures. Post-depositional cracking, dry bone fractures, thermo-clastic can duplicate some percussion marks, or affect adhering flakes.

Concerning the impact of differential preservation on percussion mark distribution, we can correlate the absence of crushing marks in our series with the lack of epiphyses. Whether this results from natural or anthropogenic differential preservation (bone fat recovery or fuel), we observed that~~,~~ shaft fragments are much more numerous than epiphyses. Yet, this type of mark is usually located close to the spongy portions [113].

To conclude, despite all these taphonomic biases, the percussion mark ratios (Number of remains with percussion marks / NISPa) recorded in our assemblages are still higher than in other sites, such as Fumane [125], Saint-Germain-la-Rivière [34] or Abric Romani [126]. This indicates systematic marrow extraction by percussion at the three compared sites.

Percussion marks: a valuable indicator of butchery traditions?

None of our assemblages shows a systematic location of the percussion marks (Figure 4-12). Thus, our results seem to be more similar to those observed at Gran Dolina TD10-1 [33]. Percussion mark frequencies indicate some tendencies depending on the samples, but their location varies according to the elements or the series, for example, the humerus (p3l) at Saint-Marcel (layer g) and Abri du Maras (series 4.2LUNG) (Figure 4). No correlation was observed between distribution and bone density or cortical thickness. However, percussion mark tendencies were not sufficient to evoke the presence of standardization. Areas with percussion marks also show tendencies rather than systematization. Before attempting to interpret them in terms of traditions, we would like to assess whether these observed tendencies are randomly caused, and if we can rule out the standardization of percussion mark distribution.

In our studied samples, we observe two cases: the first with random distribution of percussion marks, and the second with standardized distribution. In the first case, such as at Riparo Tagliente, we can suggest that several groups or individuals extracted marrow differently during recurrent occupations – palimpsest [53,54,97,127]. Due to the limited number of remains at Riparo Tagliente, for example, both layers were analysed together, erasing possible differences. The high fragmentation of the bones to extract yellow and red marrow, in order to use the cancellous portions as fuel or to make tools, such as retouchers, could also erase the systematic percussion areas produced during yellow marrow extraction. Indeed, these activities could produce more percussion marks in different locations depending on the use of the bone fragment. The entire butchery '*chaîne opératoire*' of the long bone before and after the marrow recovery should be ~~taking~~ into account in each site and assemblage. Otherwise, respectively for the levels 35 and 37 37.4 % and 35.0 % of the bones were burnt, almost all of them undetermined taxonomically. This shows a probable used of bone as fuel resulting in a loss of information with a supplementary breakage caused by the heat. Moreover, in both levels, a non-negligible number of retouchers were identified. Supplementary fractures and percussion marks could be the result of the use of the bone ~~remain~~ as a tool.

The second case refers to bones where percussion marks are not randomly distributed, such as at Abri du Maras or Saint-Marcel (tibias only) regarding the all levels studied. Such cases suggest a yellow marrow extraction tradition or an intuitive way to break bones. We tested whether the identified standardization is similar to the intuitive pattern or not, in order to interpret the standardization as culturally induced. Some features of percussion mark distribution at Abri du Maras are non-random and are very different to the intuitive pattern (layer 4.1, humerus and femur; layer 4.2 of humerus of the LUNG series and radio-ulna of the MUNG series). This seems to imply probable butchery traditions for some elements, as suggested by our experimental data. Nevertheless, in layer 4.2, this non-random feature only concerns one bone of each sample, i.e., the LUNG (humerus) and the MUNG (radio-ulna) series. This difference could be explained by a dissimilar treatment of the two different-sized ungulates in the two layers. Data from the Abri du Maras assemblages demonstrate that one assemblage with non-random and counter-intuitive percussion mark distribution in one level could influence a larger assemblage of one site. In other words, standardization could be highlighted in the occupation palimpsest. The association of two assemblages can be relevant, given the statistically significant results, as observed at Abri du Maras. However, standardization could only be highlighted by taking into account several layers, as at Saint Marcel.

In the case of the absence of randomization and counter-intuitive patterns, the hypothesis of a butchery tradition can be reasonably assumed when distribution is standardized. Specific and systematic yellow marrow extraction know-how could be partially erased by taphonomy or additional activities after marrow recovery. Furthermore, the marrow extraction process could necessitate breaking the diaphysis several times to recover the highest quantity of marrow [113,128]. Consequently, it could be possible to identify standardized practices for some long bones, but not for all of them.

This methodological approach, which tests the standardized and counterintuitive distribution with the chi-square test allows us to reveal possible butchery traditions in the assemblage of Abri du Maras layer 4.1, whereas the analysis of percussion mark frequencies failed to do so [26]. Together, the two Saint-Marcel layers demonstrate the existence of one tradition only for tibias. Thus, groups with similar practices may have occupied the cave, regarding the tibia. They could have different practices on the other long bones.

At Abri du Maras, Neandertal groups with marrow recovery skills may have transmitted standardized practices and gestures. Furthermore, the practices of the group(s) in layer 4.1 seem to have been different from those of the group(s) in layer 4.2. This latter group processed bones differently,

depending on the size of the ungulate. The absence of a clear systematization of percussion marks location in the Abri du Maras series could be explained in two ways: a complex "*chaîne opératoire*", or the occasional passage of groups with different butchery practices, or both.

However, this methodology is limited to the number of preserved areas with percussion marks. In our study, the emblematic example is the layers of Riparo Tagliente. Percussion mark frequencies indicate differences in bone treatment. However, due to the scarcity of remains, the chi-square analyses do not discern differences between layers 35 and 37. Thus, it is not possible to establish whether the lack of differences is due to the combined study of both layers or to the absence of butchery traditions. The methodology thus needs to be refined to resolve such questions.

The distribution of the recorded percussion marks is never systematic at all the sites where percussion mark distribution was studied: in Abri du Maras, Saint-Marcel Cave, Riparo Tagliente, Gran Dolina[33], Fumane [125] and Saint-Germain-la-Rivière [34] with the exception of Bolomor (layer IV). In this specific layer of Bolomor, we observed one or two systematic areas with percussion marks. This could highlight a very original marrow extraction practice with specific marrow consumption, perhaps heated, that can be supported by the higher proportion of mixed angles [128].

Interrelated traditions among technological and subsistence strategies

The hunting strategies of the three sites are different, as Neandertal groups selected different cervid species according to the sites. However, almost all the samples were practically monospecific, except the 4.2LUNG (Abri du Maras). Technical behaviour varied from one site to another with changes between some layers. The comparison of these differences with possible butchery traditions enables us to conduct an in-depth investigation of the diversity of Neandertal behaviour at the intra or inter-site scale.

At Riparo Tagliente, technical strategies change between layer 35 and 37 with the emergence of laminar core technology, whereas hunting strategies remained similar[15,129,130]. The Neandertal groups occupying those layers chose to hunt only roe deer, focusing on the ecological specialization of medium to large ungulates. It was not possible to identify specific standardized marrow recovery.

At the inter-site scale, the sites of Abri du Maras and Saint-Marcel are relatively close both geographically and chronologically (Figure 1). It is possible to propose the presence of the same groups with similar butchery traditions. However, the main core technology is Levallois/other technologies (cores on flakes or nodules, discoid-type cores) for Abri du Maras and discoid for Saint Marcel. Neandertal groups

were highly mobile, and each assemblage can be due to complex occupation events depending on the season, animal migration or raw material requirements [131,132]. Furthermore, each layer was an occupation palimpsest showing several successive occupations by one or several groups. Hunting strategies are characterized by an almost mono-specific spectrum of middle-sized ungulates for layer 4.1 of Abri du Maras and both layers of Saint-Marcel Cave. The site was occupied at a particular period of the year: autumn for Abri du Maras layer 4.1 and ~~the~~ spring-summer ~~period~~ for Saint-Marcel Cave and for layer 4.2 of Abri du Maras. However, yellow marrow is extracted differently at those two sites, in particular for metapodials. Thus, these results show that the two sites were occupied by different groups with no marrow recovery traditions at a regional scale, between Abri du Maras and Saint-Marcel Cave. Moreover, technical strategies were quite different in the two sites (Levallois and discoid), corroborating the hypothesis of groups with different cultural traditions and hunting and butchery strategies [43]. Besides, the study only concerned two sites in that region, therefore, it should be expanded to other regional sites. Moreover, the contemporaneity of the sites is relative, since we are considering on large time scales. Nevertheless, these first results are promising, showing the importance of such a study for understanding the dynamics of occupation of Neanderthal sites, in particular.

Regarding the intra site-layer comparison, the technical strategies are similar between both layers g and h of Saint-Marcel cave. Moreover, hunting strategies are also similar. The red deer were the most hunted species in the two layers [73]. These common strategies devoted to one species could be reflected by similar butchery practices, as attested by the tibias and probably the metapodials.

At the Abri du Maras layers 4.1 and 4.2, technological behaviours differ slightly from one layer to the other (same diversity of core technologies but more retouched material for layer 4.2). We observe also a change of the prey choices regarding hunting strategies. Layer 4.1 is characterized by selective reindeer hunting [54]. We note standardized practices and thus possible specialization in marrow extraction regarding the stylopodials. The hunting and marrow recovery strategies are different in layer 4.2. Reindeer still dominates the faunal spectrum, but the proportions of other large ungulates are higher. We observe distinct marrow extraction methods for the different ungulate class-sizes for each assemblage. The breakage of the humerus is more standardized for large ungulates and the breakage of the radio-ulna is more standardized for middle-sized ungulates. This site illustrates slight changes in butchery traditions over time between two different phases of occupations. The groups occupying layer 4.2 extracted marrow differently depending on the size of the herbivores. Therefore, it was probable that two different groups with their own butchery traditions occupied the site over time.

Only some long bones from Abri du Maras and Saint-Marcel Cave were broken in a standard way. Moreover, the standardly broken bones were different depending on the site or the series. This bone treatment could point to task division or time-delayed consumption [24,133,134]. Based on ethnographic studies, we can propose several hypotheses to explain differential bone treatment. Several works focusing on current hunter-gatherer societies report that some bones were cracked directly on the kill-site for immediate marrow consumption (“snack”), while others were packed and transported to the base camp for delayed consumption; e.g. [24,133–137]. Hunters seemed to prefer zeugopodials and metapodials for snacking and raw consumption. In this case, the bone remains were abandoned on the site. The femurs and humeri were transported to the base camp to be shared by the group. They were cooked and later broken to be eaten. Binford [119] observed task distribution for femurs, which were broken by women at the camp base. Some groups, such as the Hadza, San, Maasai or Siberian groups as Evenks or Evens process carcasses differently, depending on the size of the hunted animals and/or depending on the skeletal element [134–136,138–143]. Nevertheless, the complexity of dynamic of carcass transport and therefore, carcass processing proves to be of an obvious degree of difficulty. If major trends can emerge, this dynamic could vary according to each carcass or situational event. Moreover, these current hunters-gatherers groups use modern tools and adapt their behavior to them, like container to cook or steel and iron axe or knife. It is complex to compare directly the carcass processes and in particular the fracture patterns. We could not apply this observation directly to our Neandertal group assemblages. However, the hypothesis of differential long bone treatment depending on the type of occupation could be backed up by the results from layer 4.1 of Abri du Maras. Only the stylopodials were traditionally broken to recover marrow, which could then have been cooked (see references above). The different treatment depending on species size observed in layer 4.2 between the 4.2MUNG and the 4.2LUNG was also observed in the current ethnographical groups (for example [136]) and also within archaeological assemblage Qesem regarding the treatment of the metapodial [140].

Our data attest to variability in Neandertal behaviour during the Middle Palaeolithic, and not only for subsistence patterns. This variability can be partly explained by the geographical extension and duration of this period associated with the diversity of environmental conditions. Each context seems to represent a balanced response to specific conditions. Based on this assumption, the existence of different butchery practices is highly plausible. Variability in traditions could be noticed in subsistence, including both hunting and butchery strategies, and technical strategies, as observed in the sites and layers of Abri du Maras, Saint-Marcel Cave and Riparo Tagliente.

Conclusion

Our results suggest the probable presence of butchery traditions in the Abri du Maras and Saint Marcel sites, particularly at Abri du Maras, where the level 4.1 series presents standardized and counter-intuitive distributions on humeri and femurs. The samples from level 4.2 show that tibias from medium-sized ungulates were processed traditionally (mainly reindeer), whereas for large ungulates, this was only the case for the humerus. This suggests a difference in marrow recovery processes related to animal size. For Saint-Marcel, the distribution of percussion marks on radio-ulnas shows standardized marrow extraction practices for the whole series, but this standardization disappears when the levels are tested separately. This difference points to a relatively minor trend in both levels, which may not be distinguishable for a single level.

The distribution of percussion marks on the metapodials of ~~the~~ Maras and Saint-Marcel is not random. However, no comparative experimental sample is available for these elements, so it is not possible to determine if the marrow was extracted intuitively or counter-intuitively.

The groups that occupied these French sites processed some long bones in a standard way. However, these elements are different depending on the series. This first analysis does not yield any information on the specificity of the characteristic patterns of these sites or levels. It was not possible to put forward hypotheses regarding Riparo Tagliente, in particular because of the limited number of bones studied. Through the comparison of all the sites, as well as intuitive experiments, based on the analyses of percussion mark distribution using exploratory analysis (MCA), we showed that for each element the Bolomor site almost always differs from the other sites and experiments. Those assemblages seem to be different from the other sites, showing particular areas marked by percussion. The additional analysis of the Fisher Exact test did not reveal a site-specific percussion pattern based on a particular percussion zone. Nevertheless, we identified one exception: the Saint-Marcel and Abri du Maras metatarsals, where the analysis revealed significant differences in the number of traces observed. For metatarsals, in these two sites, despite the absence of comparative experimental data, we can suggest the presence of traditional marrow extraction. In addition, for this element only, the traditions appear to be different from each other. For other sites and elements, it is not possible to discriminate between one site and another. These results suggest that despite relatively poor surface conservation, percussion marks are robust enough to demonstrate the existence of butchery traditions in anthropogenic accumulations during the Middle Palaeolithic. In this paper, we established how several taphonomic modifications could affect percussion

traces in an archaeological context in Abri du Maras, Saint-Marcel and Riparo Tagliente. We suggest taking into consideration the degree of surface illegibility in percussion mark counts, especially for pits and grooves. Furthermore, differential preservation influences some traces, in particular, crushing marks.

Such work based on the distribution of percussion marks is certainly time-consuming, but it enables us to further the debate on the socio-cultural practices of Neandertal groups. We need to pursue this research and increase the corpus of sites to enhance our understanding of the different standardized and counter-intuitive practices for the Middle and Upper Palaeolithic in order to discern differences between Neandertal and modern human groups. Beside, a current works has highlighted differences in thumb morphology implying that Neandertals have a better grip on voluminous objects than modern human [144]. These results could question the influence of the grip in the way to extract marrow with hammerstone, and how this could shape some traditional practices.

In the future, a new methodological approach based on spatial analysis to study percussion mark distribution should be tested in archaeological context [50]. This method takes into account the spatial relation of percussion traces and the preservation of long bone remains, and facilitates analyses of the marks without the arbitrary division of long bones. Moreover, this method, using GIS analyses, has provided results for intuitive experiments. The comparison between the intuitive pattern and the archaeological series could fine-tune our comprehension of Neandertal and modern human butchery behaviours. It may also be necessary to carry out complementary experiments based on intuitive ways of recovering yellow marrow, in particular, on metapodials.

This study brings new data relating to the social structure of Neandertal groups. The group composition of le Rozel with a majority of children and adolescents bring new evidence to interrogate the relation between the group members [145]. This group is composed of a relatively reduced number of individuals, who repeatedly occupied a site and a layer, with different subsistence or technical strategies. The study of this site will put our results into perspective and further our understanding of socio-cultural dynamics. We can now consider the practice of bone marrow recovery as one of the structuring elements of a more global reflection on interactions and intergenerational transmission.These butchery traditions highlighted complex practices within human groups and practices that may diverged between layers of a site or show a certain continuity of butchering know-how. Our results interrogate if the traditions of lithic, hunting and butchery could be intertwined.

Acknowledgments

Fieldworks at Abri du Maras were supported by the Regional Office of Archaeology in Rhône-Alpes, the French Ministry of Culture and Communication and the Ardèche Department through several scientific programs. L. Byrne, an official translator and native English speaker, edited the English manuscript.

Conflict of interest disclosure

The authors of this preprint declare that they have no financial conflict of interest with the content of this work

Funding

CD, DV, TS and LC was supported by the Fondation Nestlé France (SJ 671–16) (https://fondation.nestle.fr/); DV is supported by the Centre d'Information des Viandes – Viande, sciences et société (SJ 334–17); RB is supported by a Ramón y Cajal research contract by the Ministry of Economy and Competitiveness (RYC2019-026386-I) and develops her work within the AEI/FEDER, EUproject PID2019-104949GB-I00, and the Generalitat de Catalunya projects 2017 SGR 836 and CLT009/18/00055; AB, CD, DV, LC, MHM and TS are supported by the Muséum national d'Histoire naturelle.

The funders had no role in study design, data collection and analysis, decision to publish, or preparation of the manuscript.

References

1. Romandini M, Thun Hohenstein U, Fiore I, Tagliacozzo A, Perez A, Lubrano V, et al. Late Neandertals And The Exploitation Of Small Mammals In Northern Italy: Fortuity , Necessity Or Hunting Variability ? Quaternaire. 2018;29: 61–67.

2. Fiore I, Gala M, Romandini M, Cocca E, Tagliacozzo A, Peresani M. From feathers to food: Reconstructing the complete exploitation of avifaunal resources by Neanderthals at Fumane cave, unit A9. Quat Int. 2016;421: 134–153. doi:10.1016/j.quaint.2015.11.142

3. Hardy BL, Moncel MH. Neanderthal use of fish, mammals, birds, starchy plants and wood 125-250,000 years ago. PLoS One. 2011;6: 0–9. doi:10.1371/journal.pone.0023768

4. Henry AG, Brooks AS, Piperno DR. Microfossils in calculus demonstrate consumption of plants and cooked foods in Neanderthal diets (Shanidar III, Iraq; Spy I and II, Belgium). Proc Natl Acad Sci U S A. 2011;108: 486–491. doi:10.1073/pnas.1016868108

5. Blasco R, Fernández Peris J, Rosell J. Several different strategies for obtaining animal resources in the late Middle Pleistocene: The case of level XII at Bolomor Cave (Valencia, Spain). Comptes Rendus Palevol. 2010;9: 171–184. doi:10.1016/j.crpv.2010.05.004

6. Guillaud E, Béarez P, Daujeard C, Defleur AR, Desclaux E, Roselló-Izquierdo E, et al. Neanderthal foraging in freshwater ecosystems: A reappraisal of the Middle Paleolithic archaeological fish record from continental Western Europe. Quat Sci Rev. 2021;252: 106731. doi:10.1016/j.quascirev.2020.106731

7. Zilhão J, Angelucci DE, Araújo Igreja M, Arnold LJ, Badal E, Callapez P, et al. Last Interglacial Iberian Neandertals as fisher-hunter-gatherers. Science (80-). 2020;367. doi:10.1126/science.aaz7943

8. Gómez-Olivencia A, Sala N, Núñez-Lahuerta C, Sanchis A, Arlegi M, Rios-Garaizar J. First data of Neandertal bird and carnivore exploitation in the Cantabrian Region (Axlor; Barandiaran excavations; Dima, Biscay, Northern Iberian Peninsula). Sci Rep. 2018;8: 10551. doi:10.1038/s41598-018-28377-y

9. Romandini M, Crezzini J, Bortolini E, Boscato P, Boschin F, Carrera L, et al. Macromammal and bird assemblages across the late Middle to Upper Palaeolithic transition in Italy: an extended zooarchaeological review. Quat Int. 2020;551: 188–223. doi:10.1016/j.quaint.2019.11.008

10. Bocherens H, Díaz-Zorita Bonilla M, Daujeard C, Fernandes P, Raynal JP, Moncel MH. Direct isotopic

evidence for subsistence variability in Middle Pleistocene Neanderthals (Payre, southeastern France). Quat Sci Rev. 2016;154: 226–236. doi:10.1016/j.quascirev.2016.11.004

11. Estalrrich A, El Zaatari S, Rosas A. Dietary reconstruction of the El Sidrón Neandertal familial group (Spain) in the context of other Neandertal and modern hunter-gatherer groups. A molar microwear texture analysis. J Hum Evol. 2017;104: 13–22. doi:10.1016/j.jhevol.2016.12.003

12. Weyrich LS, Duchene S, Soubrier J, Arriola L, Llamas B, Breen J, et al. Neanderthal behaviour, diet, and disease inferred from ancient DNA in dental calculus. Nature. 2017;544: 357–361. doi:10.1038/nature21674

13. Power RC, Salazar-García DC, Rubini M, Darlas A, Havarti K, Walker M, et al. Dental calculus indicates widespread plant use within the stable Neanderthal dietary niche. J Hum Evol. 2018;119: 27–41. doi:10.1016/j.jhevol.2018.02.009

14. Richards MP, Schmitz RW. Isotope evidence for the diet of the Neanderthal type specimen. Antiquity. 2008;82: 553–559. doi:10.1017/S0003598X00097210

15. Bocherens H, Drucker DG, Billiou D, Patou-Mathis M, Vandermeersch B. Isotopic evidence for diet and subsistence pattern of the Saint-Césaire I Neanderthal: Review and use of a multi-source mixing model. J Hum Evol. 2005;49: 71–87. doi:10.1016/j.jhevol.2005.03.003

16. Jaouen K, Colleter R, Pietrzak A, Pons ML, Clavel B, Telmon N, et al. Tracing intensive fish and meat consumption using Zn isotope ratios: Evidence from a historical Breton population (Rennes, France). Sci Rep. 2018;8: 1–12. doi:10.1038/s41598-018-23249-x

17. Morin E, Meier J, Guennouni K El, Moigne A, Lebreton L. New evidence of broader diets for archaic Homo populations in the New evidence of broader diets for archaic Homo populations in the northwestern Mediterranean. 2019. doi:10.1126/sciadv.aav9106

18. Blasco R, Fernández Peris J. A uniquely broad spectrum diet during the Middle Pleistocene at Bolomor Cave (Valencia, Spain). Quat Int. 2012;252: 16–31. doi:10.1016/j.quaint.2011.03.019

19. Pelletier M, Desclaux E, Brugal J-P, Texier P-J. The exploitation of rabbits for food and pelts by Last Interglacial Neandertals. Quat Sci Rev. 2019;224: 105972. doi:10.1016/j.quascirev.2019.105972

20. Blasco R, Rosell J, Smith KT, Maul LC, Sañudo P, Barkai R, et al. Tortoises as a dietary supplement: A view from the Middle Pleistocene site of Qesem Cave, Israel. Quat Sci Rev. 2016;133: 165–182.

doi:10.1016/j.quascirev.2015.12.006

21. Lloveras L, Salazar R, García-Argüelles P, Nadal J. Birds and Epipalaeolithic hunter-gatherers in northeast Iberia. The case of the Balma del Gai site. Quat Int. 2020. doi:10.1016/j.quaint.2020.03.010

22. Costamagno S, Rigaud J-P. L'exploitation de la graisse au Paléolithique. In: Costamagno S, editor. Histoire de l'alimentation humaine : entre choix et contraintes. Paris: CTHS (Actes des congrès des sociétés historiques et scientifiqu; 2013. pp. 134–152.

23. Speth JD. The Paleoanthropology and Archaeology of Big-Game Hunting Protein, Fat, or Politics? Springer. 2010.

24. Binford LR. Bones: Ancient Men and Modern Myths. Academic P. New-York; 1981.

25. Jin JJH, Mills EW. Split phalanges from archaeological sites: Evidence of nutritional stress? J Archaeol Sci. 2011;38: 1798–1809. doi:10.1016/j.jas.2011.03.013

26. Vettese D, Daujeard C, Blasco R, Borel A, Caceres I, Moncel MH. Neandertal long bone breakage process: Standardized or random patterns? The example of Abri du Maras (Southeastern France, MIS 3). J Archaeol Sci Reports. 2017;13: 151–163. doi:10.1016/j.jasrep.2017.03.029

27. Daujeard C, Moncel MH. On Neanderthal subsistence strategies and land use: A regional focus on the Rhone Valley area in southeastern France. J Anthropol Archaeol. 2010;29: 368–391. doi:10.1016/j.jaa.2010.05.002

28. Costamagno S, Liliane M, Cédric B, Bernard V, Bruno M. Les Pradelles (Marillac-le-Franc , France): A mousterian reindeer hunting camp ? 2006;25: 466–484. doi:10.1016/j.jaa.2006.03.008

29. Patou-Mathis M. Neanderthal Subsistence Behaviors in Europe. Int J Osteoarchaeol. 2000;10: 379–395.

30. Niven L, Steele TE, Rendu W, Mallye J, Mcpherron SP, Soressi M, et al. Neandertal mobility and large-game hunting : The exploitation of reindeer during the Quina Mousterian at Chez-Pinaud Jonzac (Charente-Maritime , France). J Hum Evol. 2012;63: 624–635. doi:10.1016/j.jhevol.2012.07.002

31. Gaudzinski-Windheuser S, Niven L. Hominin Subsistence Patterns During the Middle and Late Paleolithic in Northwestern Europe. 2009; 99–111. doi:10.1007/978-1-4020-9699-0_7

32. Castel JC, Discamps E, Soulier MC, Sandgathe D, Dibble HL, McPherron SJP, et al. Neandertal subsistence strategies during the Quina Mousterian at Roc de Marsal (France). Quat Int. 2017;433: 140–156. doi:10.1016/j.quaint.2015.12.033

33. Blasco R, Rosell J, Domínguez-Rodrigo M, Lozano S, Pastó I, Riba D, et al. Learning by Heart: Cultural Patterns in the Faunal Processing Sequence during the Middle Pleistocene. Petraglia MD, editor. PLoS One. 2013;8: e55863. doi:10.1371/journal.pone.0055863

34. Masset C, Costamagno S, Cochard D, Laroulandie V. La fracturation osseuse : du fait technique à l’essai d’interprétation sociétale L’exemple de l’antilope saïga du gisement magadelenien de Saint-Germain -la-Rivière (Gironde). Bull la Société préhistorique française. 2016;113: 691–712.

35. Soulier MC. Exploring meat processing in the past: Insights from the Nunamiut people. PLoS One. 2021;16: 1–21. doi:10.1371/journal.pone.0245213

36. Pizarro-Monzo M, Prendergast ME, Gidna AO, Baquedano E, Mora R, Gonzalez-Aguilera D, et al. Do human butchery patterns exist? A study of the interaction of randomness and channelling in the distribution of cut marks on long bones. J R Soc Interface. 2021;18: 20200958. doi:10.1098/rsif.2020.0958

37. Lyman RL. Vertebrate Taphonomy. Cambridge: Cambridge University Press; 1994.

38. Soulier M-C, Costamagno S. Let the cutmarks speak! Experimental butchery to reconstruct carcass processing. J Archaeol Sci Reports. 2017;11: 782–802. doi:10.1016/j.jasrep.2016.12.033

39. Daujeard C, Abrams G, Germonpré M, Le Pape J-M, Wampach A, Di Modica K, et al. Neanderthal and animal karstic occupations from southern Belgium and south-eastern France: Regional or common features? Quat Int. 2016. doi:10.1016/j.quaint.2016.02.009

40. Abrams G. Palaeolithic Bone Retouchers From Belgium: A Preliminary. In: Hutson JM, García-moreno A, Noack ES, Turner E, Villaluenga A, Gaudzinski-Windheuser S, editors. The Origins of Bone Tool Technologies. Mainz: Verlag des Römiisch-Germanischen Zentralmuseums; 2018. pp. 197–214.

41. Patou-Mathis M, Schwab C. Retouchoirs, compresseurs, percuteurs... Os a impressions et éraillures? Cahier X. Société pr. Paris; 2002.

42. Moncel M-H, Daujeard C. The variability of the Middle Palaeolithic on the right bank of the Middle

Rhône Valley (southeast France): Technical traditions or functional choices? Quat Int. 2012;247: 103–124. doi:10.1016/j.quaint.2010.10.030

43. Moncel M-H, Allué E, Bailon S, Barshay-Szmidt C, Béarez P, Crégut É, et al. Evaluating the integrity of palaeoenvironmental and archaeological records in MIS 5 to 3 karst sequences from southeastern France. Quat Int. 2015;378: 22–39. doi:10.1016/j.quaint.2013.12.009

44. Stapert D. Neanderthal children and their flints. PalArch's J Archaeol Northwest Eur 1. 2007;2: 16–40.

45. Conard NJ, Serangeli J, Böhner U, Starkovich BM, Miller CE, Urban B, et al. Excavations at Schöningen and paradigm shifts in human evolution. J Hum Evol. 2015;89: 1–17. doi:10.1016/j.jhevol.2015.10.003

46. Delagnes A, Rendu W. Shifts in Neandertal mobility, technology and subsistence strategies in western France. J Archaeol Sci. 2011;38: 1771–1783. doi:10.1016/j.jas.2011.04.007

47. Saladié P, Huguet R, Díez C, Rodríguez-Hidalgo A, Cáceres I, Vallverdú J, et al. Carcass transport decisions in Homo antecessor subsistence strategies. J Hum Evol. 2011;61: 425–446. doi:10.1016/j.jhevol.2011.05.012

48. Stiner MC, Gopher A, Barkai R. Hearth-side socioeconomics, hunting and paleoecology during the late Lower Paleolithic at Qesem Cave, Israel. J Hum Evol. 2011;60: 213–233. doi:10.1016/j.jhevol.2010.10.006

49. Moclán A, Domínguez-Rodrigo M. An experimental study of the patterned nature of anthropogenic bone breakage and its impact on bone surface modification frequencies. J Archaeol Sci. 2018;96: 1–13. doi:10.1016/j.jas.2018.05.007

50. Stavrova T, Borel A, Daujeard C, Vettese D. A GIS based approach to long bone breakage patterns derived from marrow extraction. Petraglia MD, editor. PLoS One. 2019;14: e0216733. doi:10.1371/journal.pone.0216733

51. Pickering TR, Egeland CP. Experimental patterns of hammerstone percussion damage on bones: Implications for inferences of carcass processing by humans. J Archaeol Sci. 2006;33: 459–469. doi:10.1016/j.jas.2005.09.001

52. Forte V. Skilled people or specialists? Knowledge and expertise in copper age vessels from central

Italy. J Anthropol Archaeol. 2019;55: 101072. doi:10.1016/j.jaa.2019.101072

53. Daujeard C, Fernandes P, Guadelli JL, Moncel MH, Santagata C, Raynal JP. Neanderthal subsistence strategies in Southeastern France between the plains of the Rhone Valley and the mid-mountains of the Massif Central (MIS 7 to MIS 3). Quat Int. 2012;252: 32–47. doi:10.1016/j.quaint.2011.01.047

54. Daujeard C, Vettese D, Britton K, Béarez P, Boulbes N, Crégut-Bonnoure E, et al. Neanderthal selective hunting of reindeer? The case study of Abri du Maras (south-eastern France). Archaeol Anthropol Sci. 2019;11: 985–1011. doi:10.1007/s12520-017-0580-8

55. Daujeard C. Exploitation du milieu animal par les Néanderthaliens dans le Sud-Est de la France Tome 1. Lyon 2. 2008.

56. Thun Hohenstein U, Peretto C. The exploitation of the faunal remains in the Mousterian levels at Riparo Tagliente (Verona, Italia). Les premiers peuplements en Eur Colloq Int [.], Rennes, 22-25 Sept 2003. 2005;35: 261–267.

57. Debard É. Le quaternaire du Bas-Vivarais d'après l'étude des remplissages d'avens, de porches de grottes et d'abris sous roche: dynamique sédimentaire, paléoclimatologie et chronologie. Université de Lyon Claude Bernard. 1988.

58. Gilles R. Présentation de silex taillés d un abri sous roche du département de l'Ardèche. Bull la Soc Prehist Fr. 1950;47 (5): 202.

59. Combier J. Le Paléolithique de l'Ardèche dans son cadre paléoclimatique. Impr Delmas. 1967;4.

60. Moncel M-H, Gaillard C, Patou-Mathis M. L'abri du Maras (Ardèche) : une nouvelle campagne de fouilles dans un site Paléolithique moyen (1993). Bull la Société préhistorique française. 1994;91: 363–368. doi:10.3406/bspf.1994.9786

61. Moncel MH, Chacón Navarro MG, La Porta A, Fernandes P, Hardy B, Gallotti R. Fragmented reduction processes: Middle Palaeolithic technical behaviour in the Abri du Maras shelter, southeastern France. Quat Int. 2014;350: 180–204. doi:10.1016/j.quaint.2014.05.013

62. Moncel M, Chacón MG, Vettese D, Courty M, Daujeard C, Eixea A, et al. Late Neanderthal short-term and specialized occupations at the Abri du Maras (South-East France, level 4.1, MIS 3). Archaeol Anthropol Sci. 2021;13: 45. doi:10.1007/s12520-021-01285-5

63. Moncel M-H, Michel V. Première datation radiométrique par U-Th d'un niveau moustérien de l'Abri

du Maras (Ardèche, France). Bull la Société préhistorique française. 2000;97: 371–375. doi:10.3406/bspf.2000.11127

64. Richard M, Falguères C, Pons-Branchu E, Bahain J-J, Voinchet P, Lebon M, et al. Contribution of ESR/U-series dating to the chronology of late Middle Palaeolithic sites in the middle Rhône valley, southeastern France. Quat Geochronol. 2015;30: 529–534. doi:10.1016/j.quageo.2015.06.002

65. Hardy BL, Moncel M-H, Daujeard C, Fernandes P, Béarez P, Desclaux E, et al. Impossible Neanderthals? Making string, throwing projectiles and catching small game during Marine Isotope Stage 4 (Abri du Maras, France). Quat Sci Rev. 2013;82: 23–40. doi:10.1016/j.quascirev.2013.09.028

66. Hardy BL, Moncel M-H, Kerfant C, Lebon M, Bellot-Gurlet L, Mélard N. Direct evidence of Neanderthal fibre technology and its cognitive and behavioral implications. Sci Rep. 2020;10: 4889. doi:10.1038/s41598-020-61839-w

67. Miras Y, Barbier-Pain D, Ejarque A, Allain E, Allué E, Marín J, et al. Neanderthal plant use and stone tool function investigated through non-pollen palynomorphs analyses and pollen washes in the Abri du Maras, South-East France. J Archaeol Sci Reports. 2020;33: 102569. doi:10.1016/j.jasrep.2020.102569

68. Moncel M-H. Les niveaux moustériens de la grotte de Saint-Marcel (Ardèche). Fouilles René Gilles. Reconnaissance de niveaux à débitage discoïde dans la vallée du Rhône. Bull la Société préhistorique française. 1998;95 (2): 141–170.

69. Moncel M-H, Daujeard C, Cregut-bonnoure É, Boulbes N, Puaud S, Debard É, et al. Nouvelles Données Sur Les Occupations Humaines Du Début Du Pléistocène Supérieur De La Moyenne Vallée Du Rhône (France). Les Sites De L'abri Des Pêcheurs, De La Baume Flandin, De L'abri Du Maras Et De La Grotte Du Figuier (Ardèche). Quaternaire. 2010;21: 385–411.

70. Defleur A, Crégut-Bonnoure E, Desclaux E, Thinon M. Presentation paleoenvironnementale du remplissage de la Baume Moula-Guercy (Soyons, Ardèche). Anthropologie. 2001;105: 69–408.

71. Crégut‐Bonnoure E, Boulbes N, Daujeard C, Fernandez P, Valensi P. Nouvelles données sur la grande faune de l'Éemien dans le Sud-Est de la FranceNew data on the large mammals fauna from the Eemian in the southeast of France. Quaternaire. 2010;21: 227–248. doi:10.4000/quaternaire.5592

72. Moncel M-H, Daujeard C, Crégut-Bonnoure E, Fernandez P, Faure M, Guérin C. L'occupation de la grotte de Saint-Marcel (Ardèche, France) au Paléolithique moyen : stratégie d'exploitation de l'environnement et type d'occupation de la grotte. L'exemple des couches i, j et j'. Bull la Société préhistorique française. 2004;101: 257–304. doi:10.3406/bspf.2004.12993

73. Daujeard C. Stratégies de chasse et modalités de traitement des carcasses par les Néanderthaliens de la grotte Saint-Marcel, Ardèche:(fouilles R. Gilles, ensemble 7). Paleo. 2004;16: 49–70.

74. Crégut‐Bonnoure E. Les Ovibovini et Caprini (Mammalia, Artiodactyla, Bovidae, Caprinae) du Plio-Pléistocène d'Europe: systématique, évolution et biochronologie. University of Claude Bernard, Lyon I. 2002.

75. Evin J, Marechal J, Marien G. Lyon Natural Radiocarbon Measurements X. Radiocarbon. 1985;27: 386–454.

76. Szmidt CC, Moncel M-H, Daujeard C. New data on the Late Mousterian in Mediterranean France: First radiocarbon (AMS) dates at Saint-Marcel Cave (Ardèche). Comptes Rendus Palevol. 2010;9: 185–199. doi:10.1016/j.crpv.2010.05.002

77. Moncel M-H. L'exploration de l'espace et la mobilité des groupes humains au travers des assemblages lithiques à la fin du pléistocène moyen et au début du pléistocène supérieur: la moyenne vallée du Rhône entre Drôme et Ardèche, France. Br Archaeol Reports. 2003;1184.

78. Bartolomei G, Broglio A, Cattani L, Cremaschi M, Guerreschi A, Leonardi P, et al. Paleolitico e mesolitico. Il Veneto nell'antichità. Preist e Protostoria. 1984;2: 167–319.

79. Bartolomei G, Broglio A, Cattani L., Cremaschi M, Guerreschi A, Mantovan E, et al. I depositi würmiani del Riparo Tagliente. Ann dell'Università di Ferrara. 1982;15; III (4: 31–105.

80. Bartolomei G, Broglio A, Guerreschi A, Leonardi P, Peretto C, Sala R. Una sepoltura epigravettiana nel deposito pleistocenico del Riparo Tagliente in Valpantena (Verona). Riv di Sci Preist. 1974;29: 101–152.

81. Zorzi F, Mezzana F. Provincia di Verona. Grezzana,. Riv di Sci Preist. 1962;17: 284–285.

82. Zorzi F, Mezzana F. Provincia di Verona. Grezzana. Riv di Sci Preist. 1963;18: 307–308.

83. Mezzana F. Oggetti d'arte mobiliare del Paleolitico scoperti al Riparo Tagliente in Valpantena (Verona). Riv di Sci Preist. 1964;19: 175–187.

84. Pasa A, Mezzana F. Riparo Tagliente (Grezzana, Verona). Riv di Sci Preist. 1964;19: 295–296.

85. Guerreschi A, Peretto C, Thun Hohenstein U. I depositi di Riparo Tagliente Preistoria veronese, contributi e aggiornamenti. Memorie de. In: Aspes A, editor. Memorie del Museo Civico di Storia Naturale di Verona. Memorie de. Verona; 2002. pp. 15–16.

86. Arnaud J, Peretto C, Panetta D, Tripodi M, Fontana F, Arzarello M, et al. A reexamination of the Middle Paleolithic human remains from Riparo Tagliente, Italy. Quat Int. 2016;425: 437–444. doi:10.1016/j.quaint.2016.09.009

87. Berto C. Distribuzione ed evoluzione delle associazioni a piccoli mammiferi nella penisola italiana durante il Pleistocene superiore. University of Ferrara. 2013.

88. Thun Hohenstein U. Strategie di sussistenza adottate dai Neandertaliani nel sitio di Riparo Tagliente (Prealpi venete). Archaeozoological Stud honour Alfredo Riedel |. 2006; 31–38.

89. Forestier H. Le Clactonien : mise en application d’une nouvelle méthode de débitage s’inscrivant dans la variabilité des systèmes de production lithique du Paléolithique ancien. Paléo. 1993;5: 53–82. doi:10.3406/pal.1993.1104

90. Boëda E. Le concept Levallois: variabilité des méthodes. Archéo édi. Paris; 1994.

91. Arzarello M, Peretto C. Nouvelles données sur les caractéristiques et l’évolution techno-économique de l’industrie moustérienne du Riparo Tagliente (Verone, Italie). In: Molines N, Moncel M-H, Morrier JL, editors. Le Premiers Peuplements En EuropeColloque International; Données Récentes Sur Les Modalités de Peuplement et Sur Le Cadre Chronostratigraphique, Géologique et Paléogéographique Des Industries Du Paléolithique Ancien et Moyen En Europe. Oxford: BAR International Series 1364, Archaeopress; 2005. pp. 281–289.

92. Berruti GLF, Bianchi E, Daffara S, Gomes M, Ceresa Genet AJ, Fontana F, et al. The use of blades and pointed tools during middle palaeolithic, the example of Riparo Tagliente (VR). Quat Int. 2020;554: 45–59. doi:10.1016/j.quaint.2020.07.016

93. Brugal JP, David F, Farizy C. Quantification d’un assemblage osseux : parametres et tableaux,. In: CEDARC, editor. Outillage peu elabore en os et en bois de cervides IV,6eme Table Ronde, Artefacts 9. Paris; 1994. pp. 143–153.

94. Barone R. Anatomie comparée des mammifères domestiques : Tome 1, Ostéologie. Vigot, editor.

Paris; 1976.

95. Lavocat R. Atlas de Préhistoire: Faunes et flores préhistoriques de L'Europe occidentale. Éditions N. Paris; 1966.

96. Pales L, Lambert C. Atlas ostéologique pour servir à l'identification des mammifères du quaternaire. Centre nat. Paris; 1971.

97. Thun Hohenstein U. Strategie di sussistenza e comportamento dell'uomo di Neanderthal. Analisi archeozoologica dei livelli musteriani del Riparo Tagliente, Grotta della Ghiacciaia (Italia) e Abric Romani (Spagna). Unniversity of Ferrara. 2001.

98. Patou-Mathis M. Les grands mammifères de la couche 5 de Müzig I (Bas-Rhin). La subsistance au Paléolithique moyen en Alsace. Anthropozoologica. 1997;25–26: 363–374.

99. Vettese D, Blasco R, Cáceres I, Gaudzinski-Windheuser S, Moncel M, Hohenstein UT, et al. Towards an understanding of hominin marrow extraction strategies: a proposal for a percussion mark terminology. Archaeol Anthropol Sci. 2020;12: 48. doi:10.1007/s12520-019-00972-8

100. Behrensmeyer AK. Taphonomic and ecologic information from bone weathering. Paleobiology. 1978;4: 150–162. doi:10.2307/2400283

101. Blumenschine RJ, Marean CW, Capaldo SD. Blind Tests of Inter-analyst Correspondence and Accuracy in the Identification of Cut Marks , Percussion Marks , and Carnivore Tooth Marks on Bone Surfaces. J Archaeol Sci. 1996;23: 493–507. doi:10.1006/jasc.1996.0047

102. Fernández-Jalvo Y, Andrews P. Atlas of Taphonomic Identifications. 2016. doi:10.1007/978-94-017-7432-1

103. Boulestin B. Approche taphonomique des restes humains. Le cas des mésolithiques de la gotte des Perrats (Agris, Charente). 1998; 435.

104. Denys C, Patou-Mathis M. Manuel de taphonomie. Errance. Paris; 2004.

105. Guadelli JL. La gélifraction des restes fauniques. Expérimentation et transfert au fossile. Ann Paleontol. 2008;94: 121–165. doi:10.1016/j.annpal.2008.05.002

106. Stiner MC, Kuhn SL, Weiner S, Bar-Yosef O. Differential Burning, Recrystallization, and Fragmentation of Archaeological Bone. J Archaeol Sci. 1995;22: 223–237.

107. Lyman RL. Quantitative paleozoology. New York: Cambridge University Press has; 2008. doi:10.2993/0278-0771-30.1.126

108. Mallye J, Thiébaut C, Mourre V, Costamagno S, Claud É, Weisbecker P. The Mousterian bone retouchers of Noisetier Cave: experimentation and identification of marks. J Archaeol Sci. 2012;39: 1131–1142. doi:10.1016/j.jas.2011.12.018

109. Daujeard C, Moncel MH, Fiore I, Tagliacozzo A, Bindon P, Raynal JP. Middle Paleolithic bone retouchers in Southeastern France: Variability and functionality. Quat Int. 2014;326–327: 492–518. doi:10.1016/j.quaint.2013.12.022

110. Moigne A, Valensi P, Auguste P, García-Solano J, Tuffreau A, Lamotte A, et al. Bone retouchers from Lower Palaeolithic sites: Terra Amata, Orgnac 3, Cagny-l'Epinette and Cueva del Angel. Quat Int. 2016;409: 195–212. doi:10.1016/j.quaint.2015.06.059

111. Outram AK. A New Approach to Identifying Bone Marrow and Grease Exploitation : Why the '" Indeterminate "' Fragments should not be Ignored. J Archaeol Sci. 2001;28: 401–410. doi:10.1006/jasc.2000.0619

112. Villa P, Mahieu E. Breakage patterns of human long bones. J Hum Evol. 1991;21: 27–48.

113. Vettese D, Stavrova T, Borel A, Marin J, Arzarello M, Daujeard C. A way to break bones ? The weight of intuitiveness. bioRxiv. 2020. doi:10.1101/2020.03.31.011320

114. Lam YM, Chen X, Pearson OM. Intertaxonomic Variability in Patterns of Bone Density and the Differential Representation of Bovid , Cervid , and Equid Elements in the Archaeological Record. Soc Am Archaeol. 1999;64: 343–362. doi:10.2307/2694204

115. Jones KT, Metcalfe D. Bare bones archaeology: Bone marrow indices and efficiency. J Archaeol Sci. 1988;15: 415–423. doi:10.1016/0305-4403(88)90039-8

116. R Core Team. R: A language and environment for statistical computing. In: Foundation for Statistical Computing, Vienna, Austria. 2019.

117. Lê S, Josse J, Husson F. FactoMineR: an R package for multivariate analysis. J Stat Softw. 2008;25: 1–18.

118. Morin E, Soulier M-C. New Criteria for the Archaeological Identification of Bone Grease Processing. Am Antiq. 2017;82: 96–122. doi:10.1017/aaq.2016.16

119. Auguste P. Introduction générale : la fossilisation. Artefacts. 1994;9: 11–14.

120. Théry-Parisot I, Costamagno S. Propriétés combustibles des ossements : données expérimentales et réflexions archéologiques sur leur emploi dans les sites paléolithiques. Gall préhistoire. 2005;47: 235–254. doi:10.3406/galip.2005.2051

121. Burch ES. The Caribou / Wild Reindeer as a Human. Am Antiq. 1972;37: 339–368.

122. Vettese D. An archaeological experiment focused on the intuitive way of long bones breakage to extract marrow V . 2. 2020. doi:10.1371/journal.pone.0216733.KEYWORDS

123. Blasco R, Rosell J, Gopher A, Barkai R. Subsistence economy and social life: A zooarchaeological view from the 300 kya central hearth at Qesem Cave, Israel. J Anthropol Archaeol. 2014;35: 248–268. doi:10.1016/j.jaa.2014.06.005

124. Capaldo SD, Blumenschine RJ. A Quantitative Diagnosis of Notches Made by Hammerstone Percussion and Carnivore Gnawing on Bovid Long Bones. Am Antiq. 1994;59: 724–748.

125. Romandini M, Nannini N, Tagliacozzo A, Peresani M. The ungulate assemblage from layer A9 at Grotta di Fumane, Italy: A zooarchaeological contribution to the reconstruction of Neanderthal ecology. Quat Int. 2014;337: 11–27. doi:10.1016/j.quaint.2014.03.027

126. Rosell J, Cáceres I, Blasco R, Bennàsar M, Bravo P, Campeny G, et al. A zooarchaeological contribution to establish occupational patterns at Level J of Abric Romaní (Barcelona, Spain). Quat Int. 2012;247: 69–84. doi:http://dx.doi.org/10.1016/j.quaint.2011.01.020

127. Daujeard C. Exploitation intensive des carcasses de Cerf dans Ie gisement paleolithique moyen de la grotte de Saint-Marcel (Ardeche). Congrès du centenaire: Un siècle de construction du discours scientifique en Préhistoire. 2004. pp. 481–497.

128. Chevillard L. Du geste au stigmate : apport de l ’ expérimentation à la caractérisation des impacts de percussion en contexte. Muséum national d’Histoire naturelle. 2018.

129. Gaudzinski-Windheuser S, Kindler L. Research perspectives for the study of Neandertal subsistence strategies based on the analysis of archaeozoological assemblages. Quat Int. 2012;247: 59–68. doi:10.1016/j.quaint.2010.11.029

130. Bocherens H, Drucker DG. Bocherens, H., & Drucker, D. (2003). Reconstructing Neandertal diet from 120,000 to 30,000 BP using carbon and nitrogen isotopic abundances. Bar Int Ser. 2003;1105:

1–8.

131. Rendu W. Hunting behavior and Neanderthal adaptability in the Late Pleistocene site of Pech-de-l'Azé I. J Archaeol Sci. 2010;37: 1798–1810. doi:10.1016/j.jas.2010.01.037

132. Shea JJ. Behavioral differences between Middle and Upper Paleolithic Homo sapiens in the East Mediterranean Levant: The roles of intraspecific competition and dispersal from Africa. J Anthropol Res. 2007;63: 449–488. doi:10.3998/jar.0521004.0063.401

133. O'Connell JF, Hawkes K, Blurton-Jones NG. Hadza Hunting, Butchering, and Bone Transport and Their Archaeological Implications. J Anthropol Res. 1988;44: 113–161.

134. Abe Y. Hunting and Butchery Patterns of the Evenki in Northern Transbaikalia, Russia. Stony brook University. 2005.

135. Binford LR. Nunamiut: Ethnoarchaeology. Academic P. New-York, Chicago, London; 1978.

136. Bunn HT, Bartram LE, Kroll EM. Variability in bone assemblage formation from Hadza hunting, scavenging, and carcass processing. J Anthropol Archaeol. 1988;7: 412–457. doi:10.1016/0278-4165(88)90004-9

137. Bartram LE, Marean CW. Explaining the "Klasies Pattern": Kua ethnoarchaeology, the Die Kelders Middle Stone Age archaeofauna, long bone fragmentation and carnivore ravaging. J Archaeol Sci. 1999;26: 9–29. doi:10.1006/jasc.1998.0291

138. Yellen JE. Cultural patterning in faunal remains: evidence from the !Kung bushmen. Exp Archaeol. 1977;271: 331.

139. Costamagno S, David F. Comparaison des pratiques bouchères et culinaires de différents groupes sibériens vivant de la renniculture. Archaeofauna. 2009;18: 9–25.

140. Blasco R, Rosell J, Arilla M, Margalida A, Villalba D, Gopher A, et al. Bone marrow storage and delayed consumption at Middle Pleistocene Qesem Cave, Israel (420 to 200 ka). Sci Adv. 2019;5: eaav9822. doi:10.1126/sciadv.aav9822

141. Gifford-Gonzalez DP. Observations of modern human settlements as an aid to archaeological interpretation. University of California, Berkeley. 1977.

142. Domínguez-Rodrigo M. The study of skeletal part profiles: an ambiguous taphonomic tool for

zooarchaeology. Complutum. 1999;10: 15–24.

143. O'Connell JF, Hawkes K, Blurton Jones N. Reanalysis of large mammal body part transport among the Hadza. J Archaeol Sci. 1990;17: 301–316. doi:10.1016/0305-4403(90)90025-Z

144. Bardo A, Moncel MH, Dunmore CJ, Kivell TL, Pouydebat E, Cornette R. The implications of thumb movements for Neanderthal and modern human manipulation. Sci Rep. 2020;10: 1–12. doi:10.1038/s41598-020-75694-2

145. Duveau J, Berillon G, Verna C, Laisné G, Cliquet D. The composition of a Neandertal social group revealed by the hominin footprints at Le Rozel (Normandy, France). Proc Natl Acad Sci. 2019;116: 201901789. doi:10.1073/pnas.1901789116

Supplementary Information

S1 Tableau: Mains size categories regarding the species involved in our analyses

S2 Tableau: Main post-depositional modifications (NR/%NR) of each sample; Saint Marcel cave layer g and h; Riparo Tagliente layer 35 and 37); Abri du Maras layer 4.1, 4.2 LUNG and 4.2 MUNG

S1 Figure: % MAU regarding each element for each layer of the studied sites.

S2 Figure: Diagram showing relative frequencies of shaft length for each layers of our sample of long bone elements

S3 Figure: NISP values for each of the measurement intervals along the length and circumference of the all types of bone together

S4 Figure: Percentage of humerus survivorship areas of each sample; Saint Marcel cave layer g (A), layer h (B); Riparo Tagliente layer 35 (C), layer 37 (D); Abri du Maras layer 4.1 (E), layer 4.2 LUNG (F), layer 4.2 MUNG.

S5 Figure: Percentage of radio-ulna survivorship areas of each sample; Saint Marcel cave layer g (A), layer h (B); Riparo Tagliente layer 35 (C), layer 37 (D); Abri du Maras layer 4.1 (E), layer 4.2 LUNG (F), layer 4.2 MUNG.

S6 Figure: Percentage of metacarpal survivorship areas of each sample; Saint Marcel cave layer g (A), layer h (B); Riparo Tagliente layer 35 (C), layer 37 (D); Abri du Maras layer 4.1 (E), layer 4.2 LUNG (F), layer 4.2 MUNG.

S7 Figure: Percentage of femur survivorship areas of each sample; Saint Marcel cave layer g (A), layer h (B); Riparo Tagliente layer 35 (C), layer 37 (D); Abri du Maras layer 4.1 (E), layer 4.2 LUNG (F), layer 4.2 MUNG

S8 Figure: Percentage of tibia survivorship areas of each sample; Saint Marcel cave layer g (A), layer h (B); Riparo Tagliente layer 35 (C), layer 37 (D); Abri du Maras layer 4.1 (E), layer 4.2 LUNG (F), layer 4.2 MUNG.

S9 Figure: Percentage of metatarsal survivorship areas of each sample; Saint Marcel cave layer g (A), layer h (B); Riparo Tagliente layer 35 (C), layer 37 (D); Abri du Maras layer 4.1 (E), layer 4.2 LUNG (F), layer 4.2 MUNG.

S10 Figure: A- MCA of full data, BOL: Bolomor, TD10: Grand Dolina TD10-1, RT: Riparo Tagliente, AM: Abri du Maras, SM: Saint-Marcel and Exp: Experiment; Int: Intuitivity, Moc: Moclan and Dominguez-Rodrigo 2018, Ham: Hammerstone on anvil and Batt: by batting: Blasco et al. 2013. NI: without impact and I: with impact B- Contribution of variables (in blue, the illustrative ones); C- Contribution of the variables for each dimension; D- Contribution for the first and second dimension

S11 Figure: MCA of full data of our site according to the qualitative and illustrative variablesS12 Figure: MCA of all layers and element taking into account only the portion and side with percussion marks

S13 Figure: Number of portion by element (NP), number of percussion marks by portion (NPPM) by element and ratio of NP/NPM; for each site and layer.

Figure 1

Click here to access/download;Figure;Figure_1_Cart_chrono_art_synt.tif

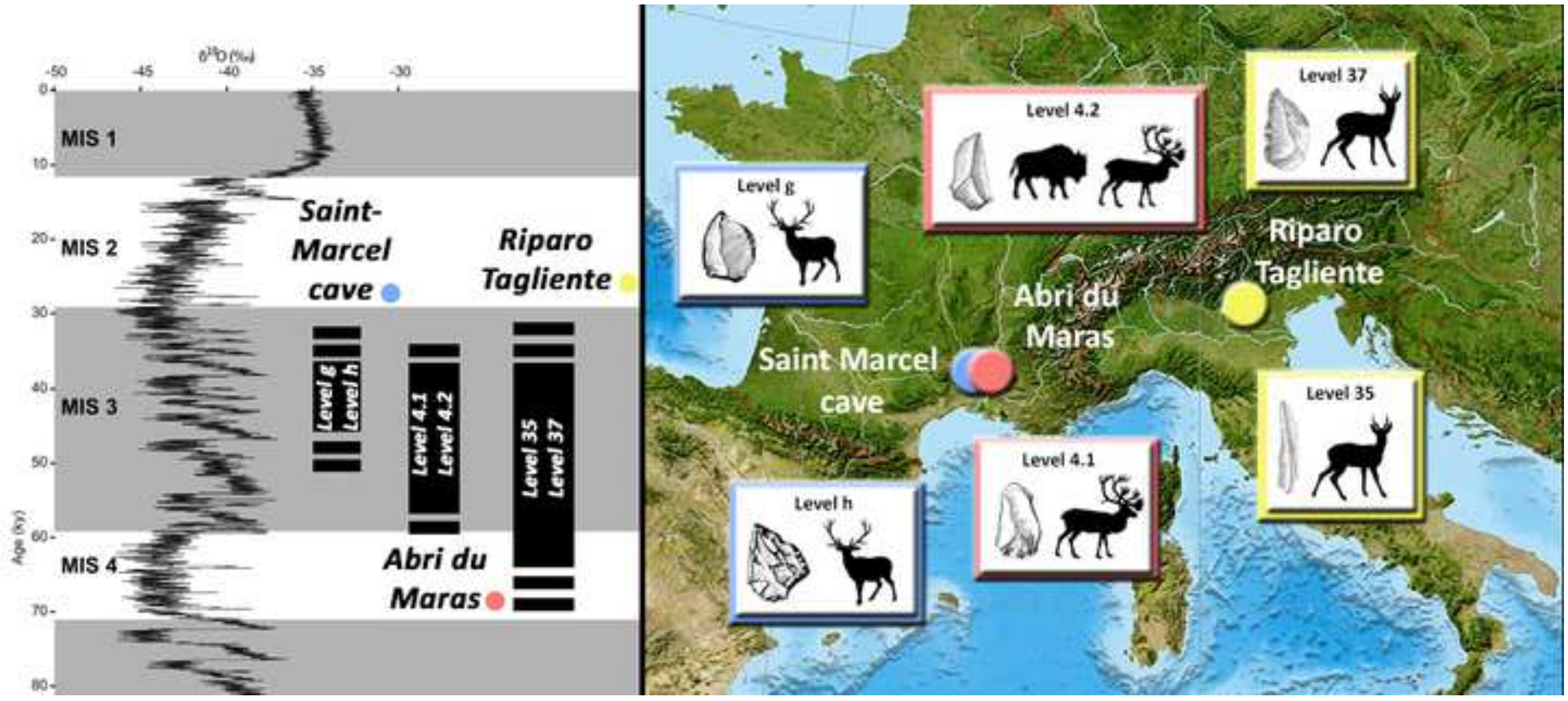

Figure 2

Click here to access/download;Figure;Figure_2_MAU_SM_AM_RT_art_synt.tif

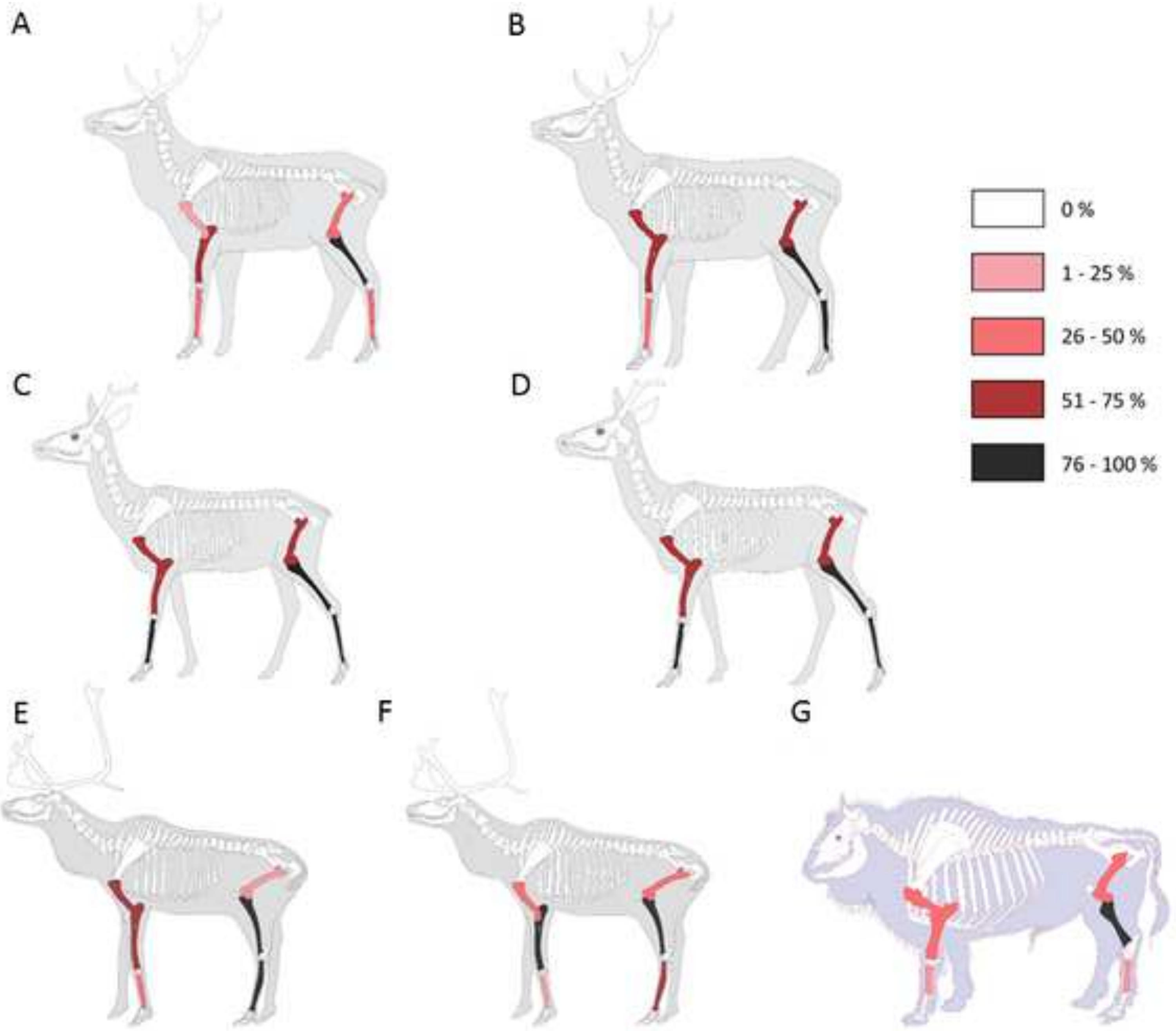

Figure 3

Click here to access/download;Figure;Figure_3_freg_pm_humerus_art_syn.TI

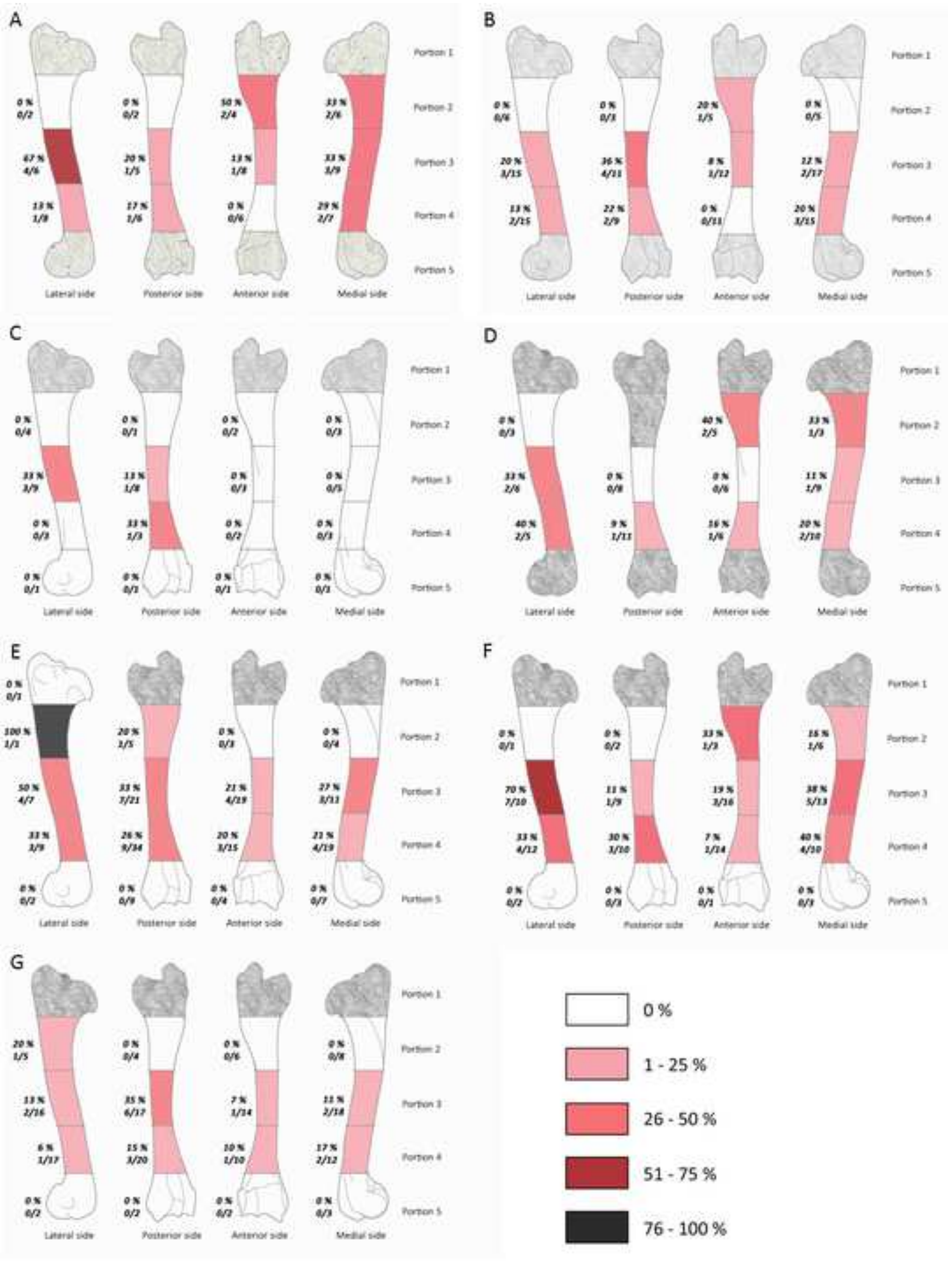

Figure 4

Click here to access/download;Figure;Figure_4_freg_pm_radio-ulna_art_syn.TIF.TIF

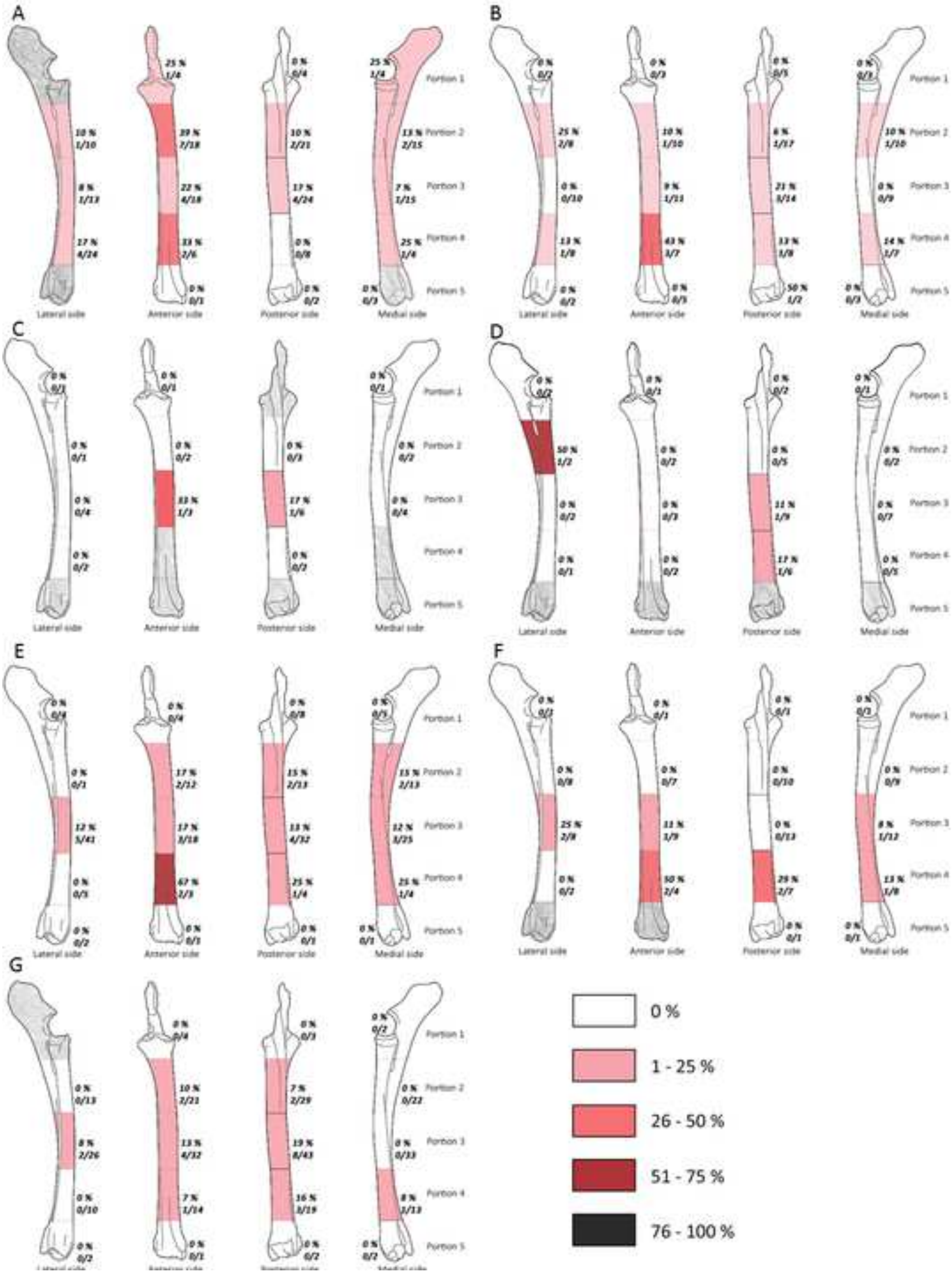

Figure 5

Click here to access/download;Figure;Figure_5_freg_pm_metacarpal_art_syn.

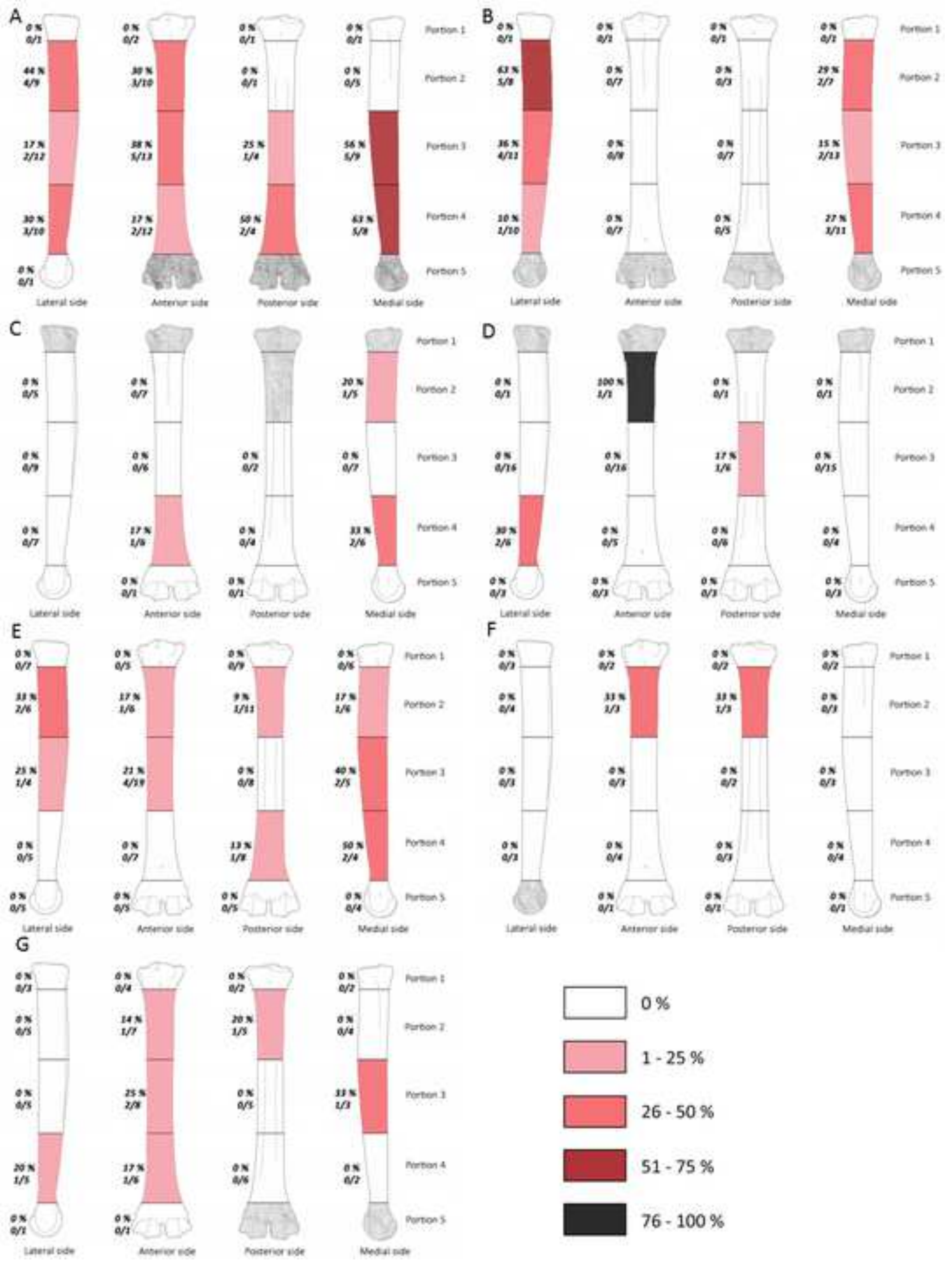

Figure 6

Click here to access/download;Figure;Figure_6_freg_pm_femur_art_syn.TIF.T

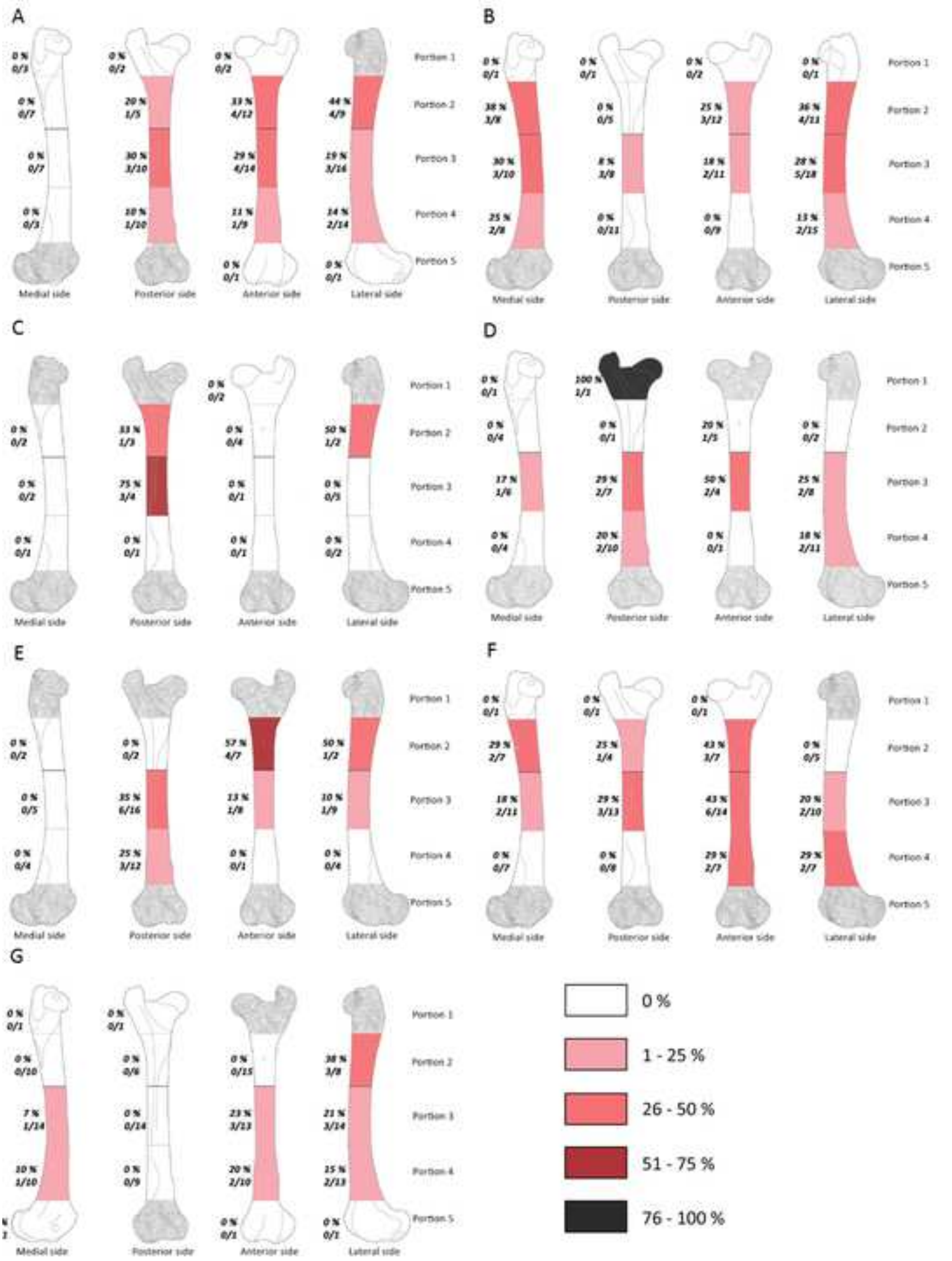

Figure 7

Click here to access/download;Figure;Figure_7_freg_pm_tibia_art_syn.TIF.TIF

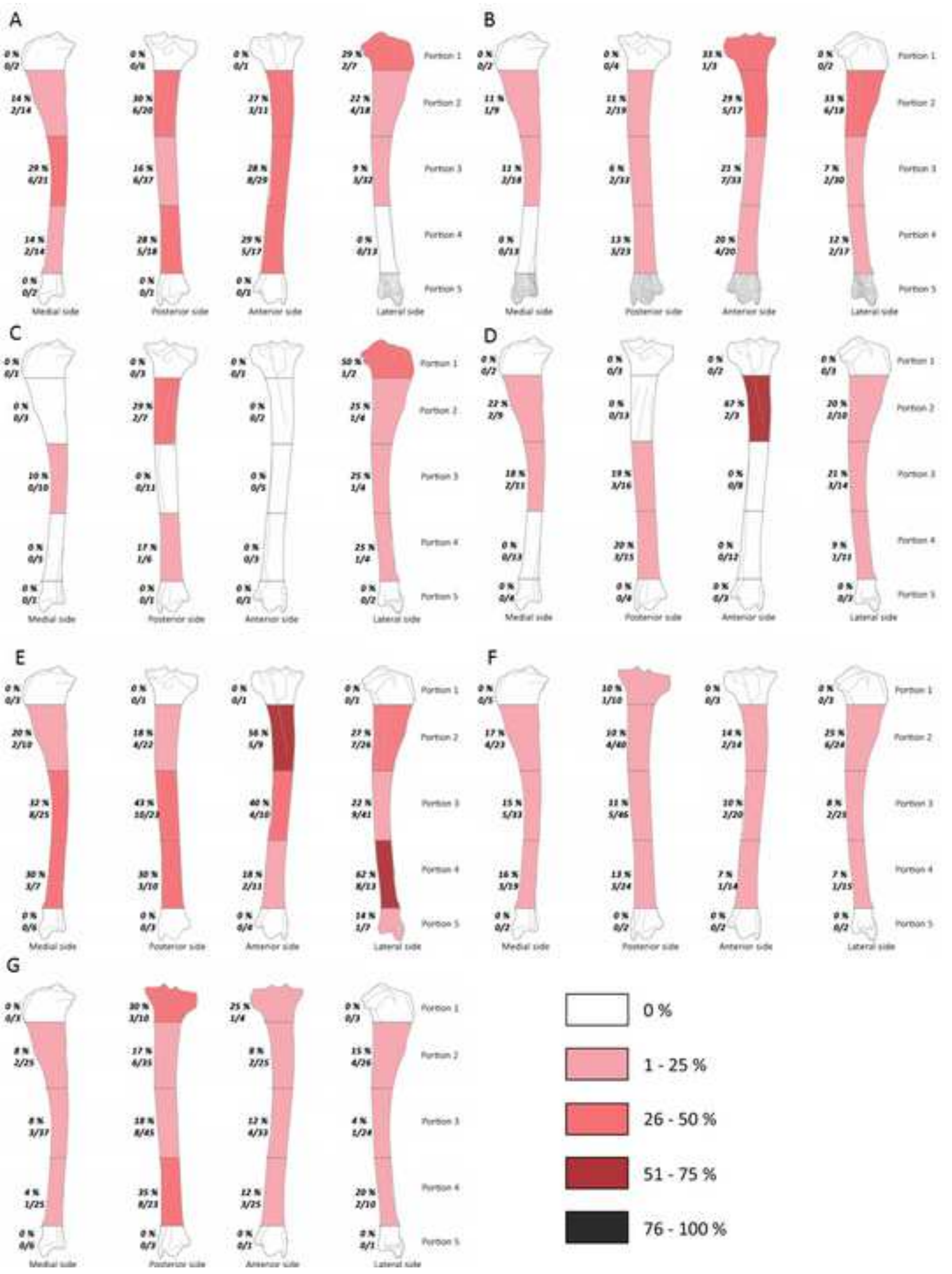

Figure 8

Click here to access/download;Figure;Figure_8_freg_pm_metatarsal_art_syn.

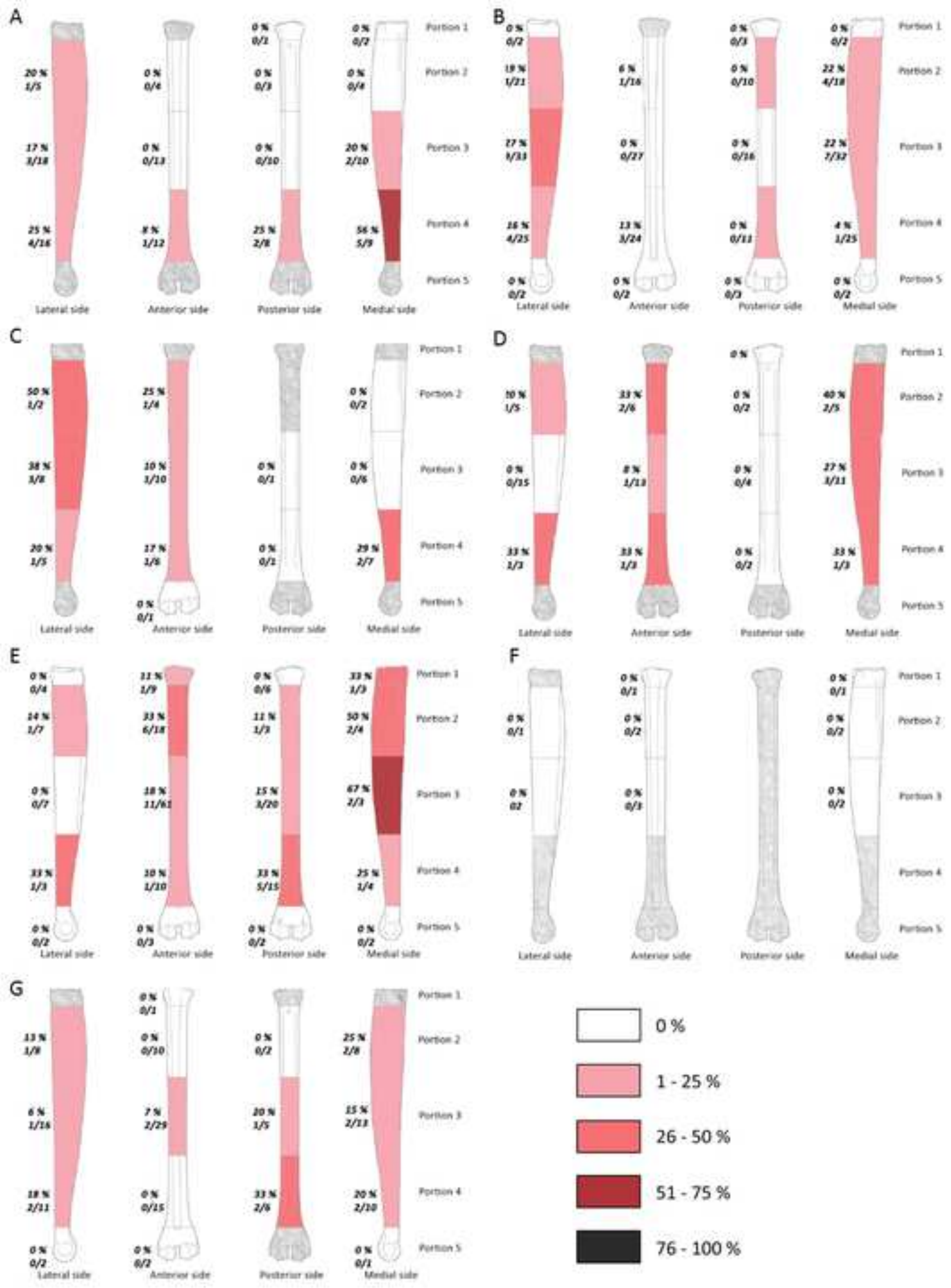

Figure 9

Click here to access/download;Figure;Figure_9_humerus_radio-ulna_barplot.tif

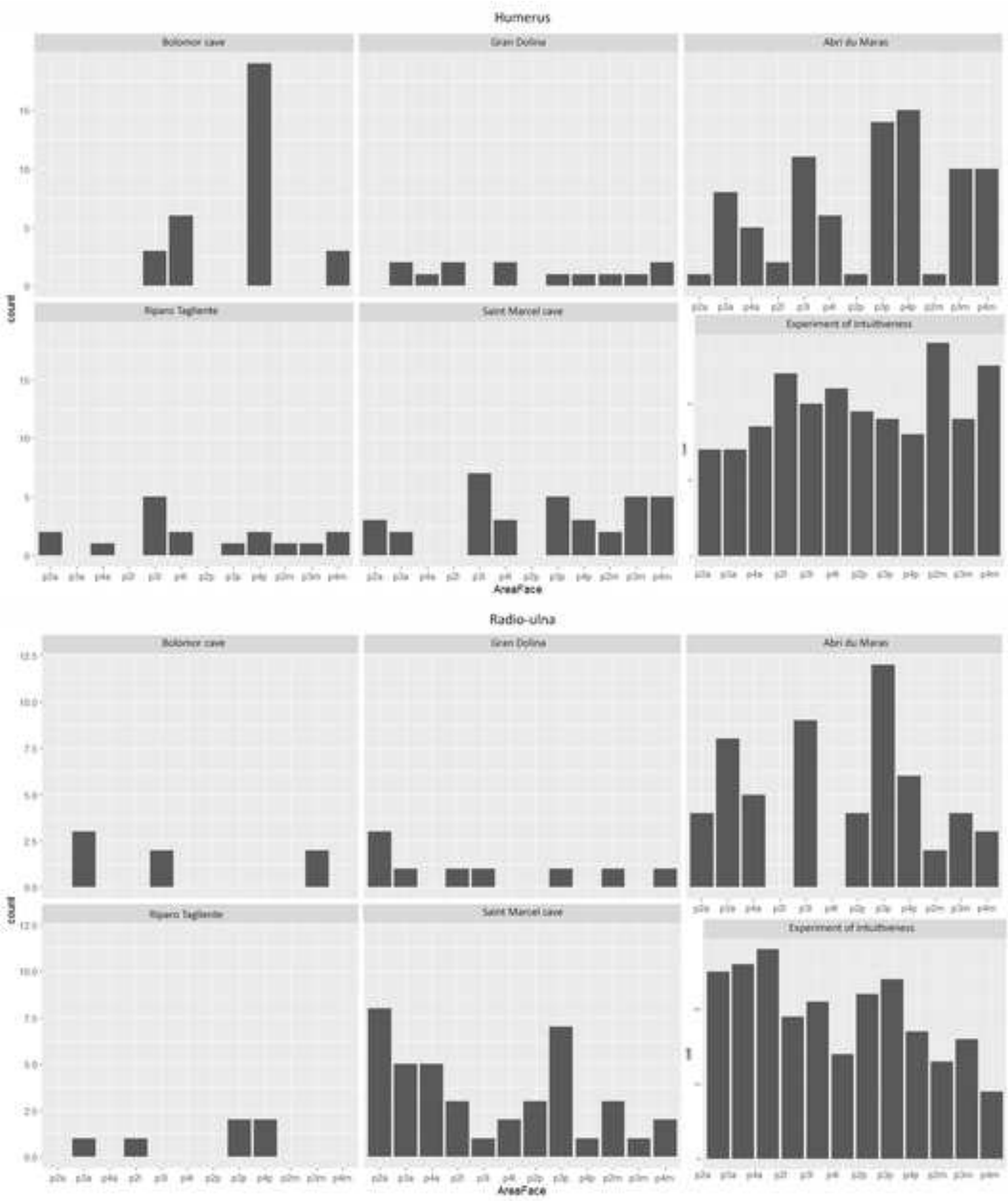

Figure 10

Click here to access/download;Figure;Figure_10_tibia_femur_barplot.tif

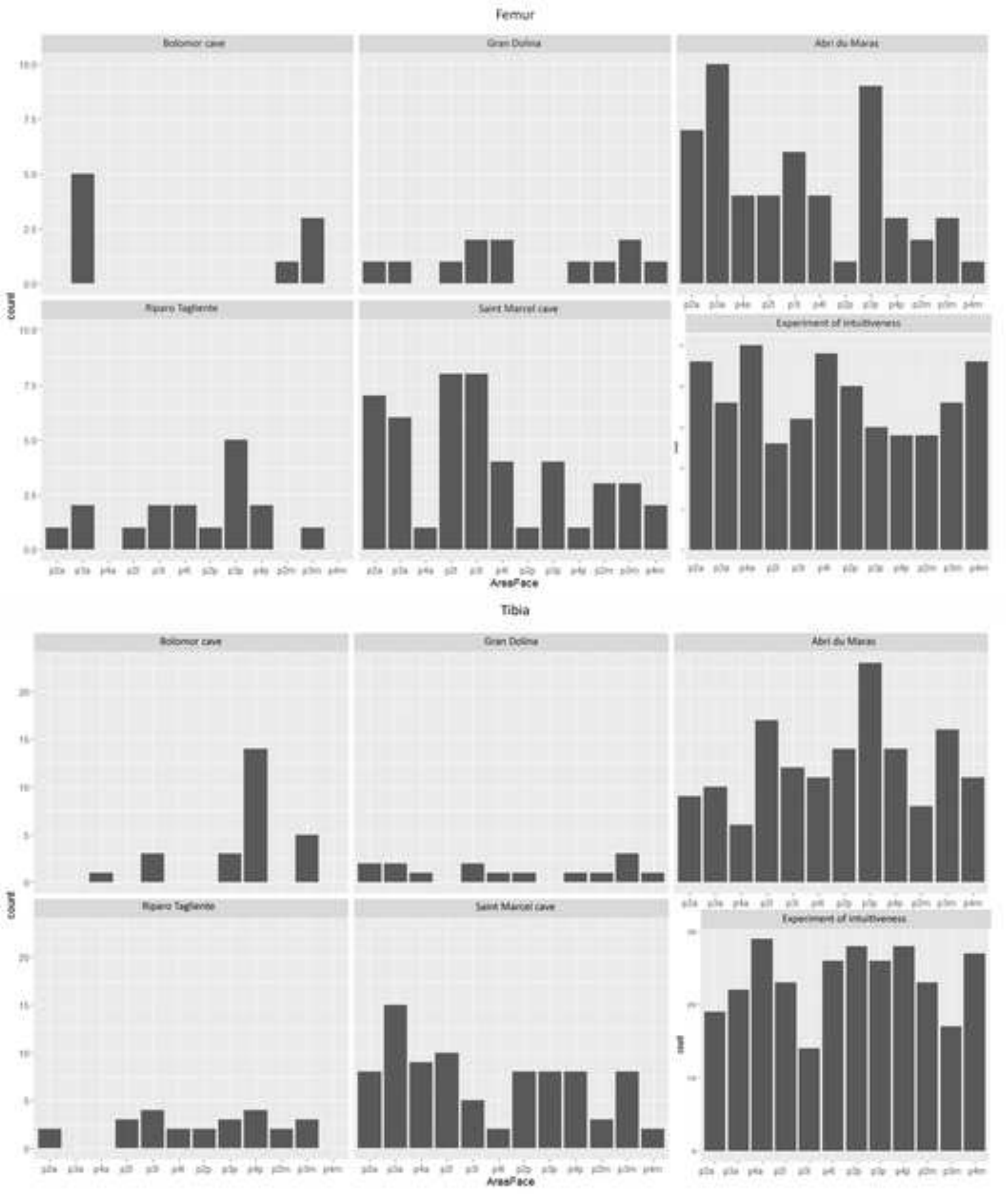

Figure 11

Click here to access/download;Figure;Figure_11_metacarpal_metatarsal_barp

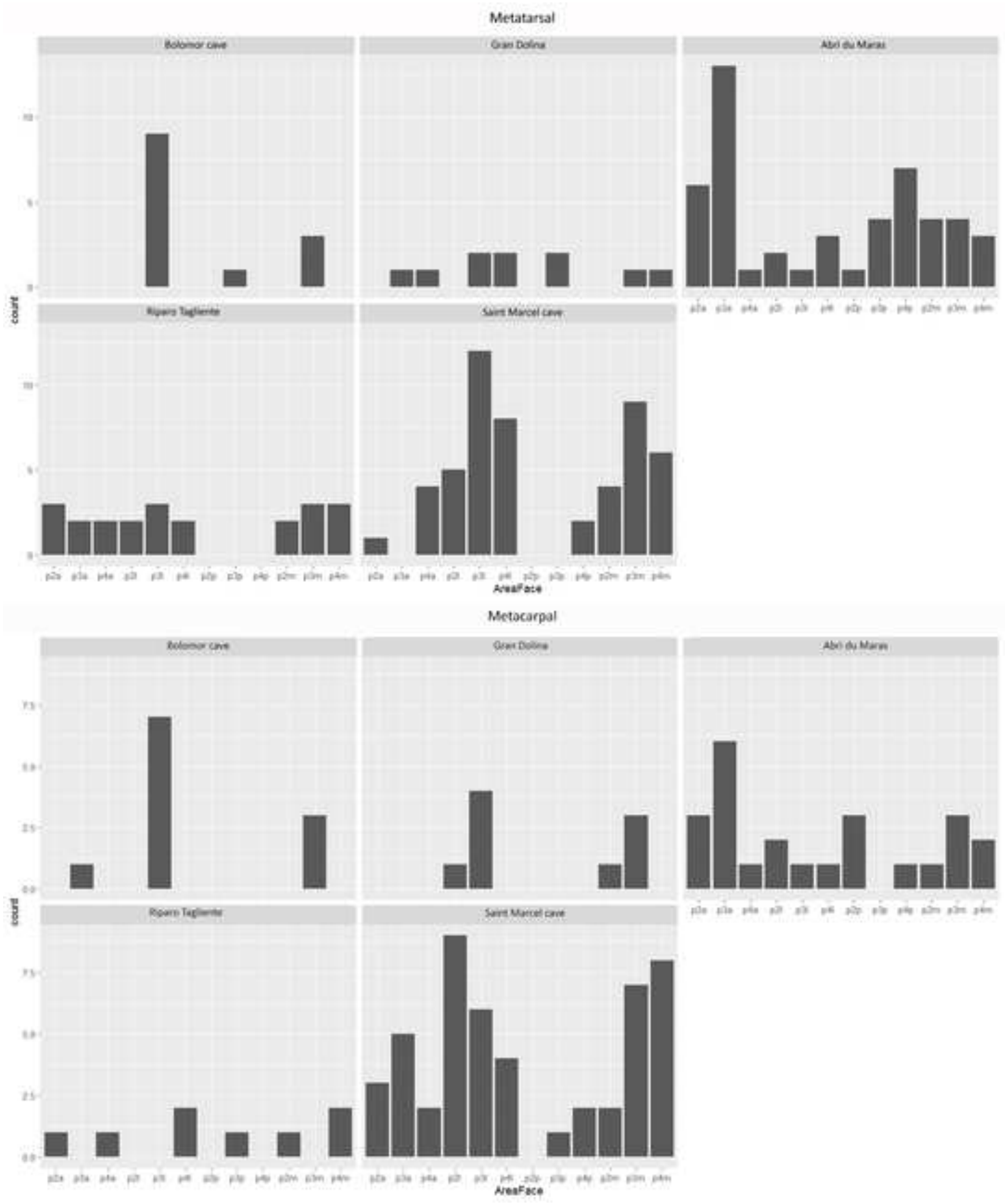

Figure 12

Click here to access/download;Figure;Figure_12_humerus_radio-

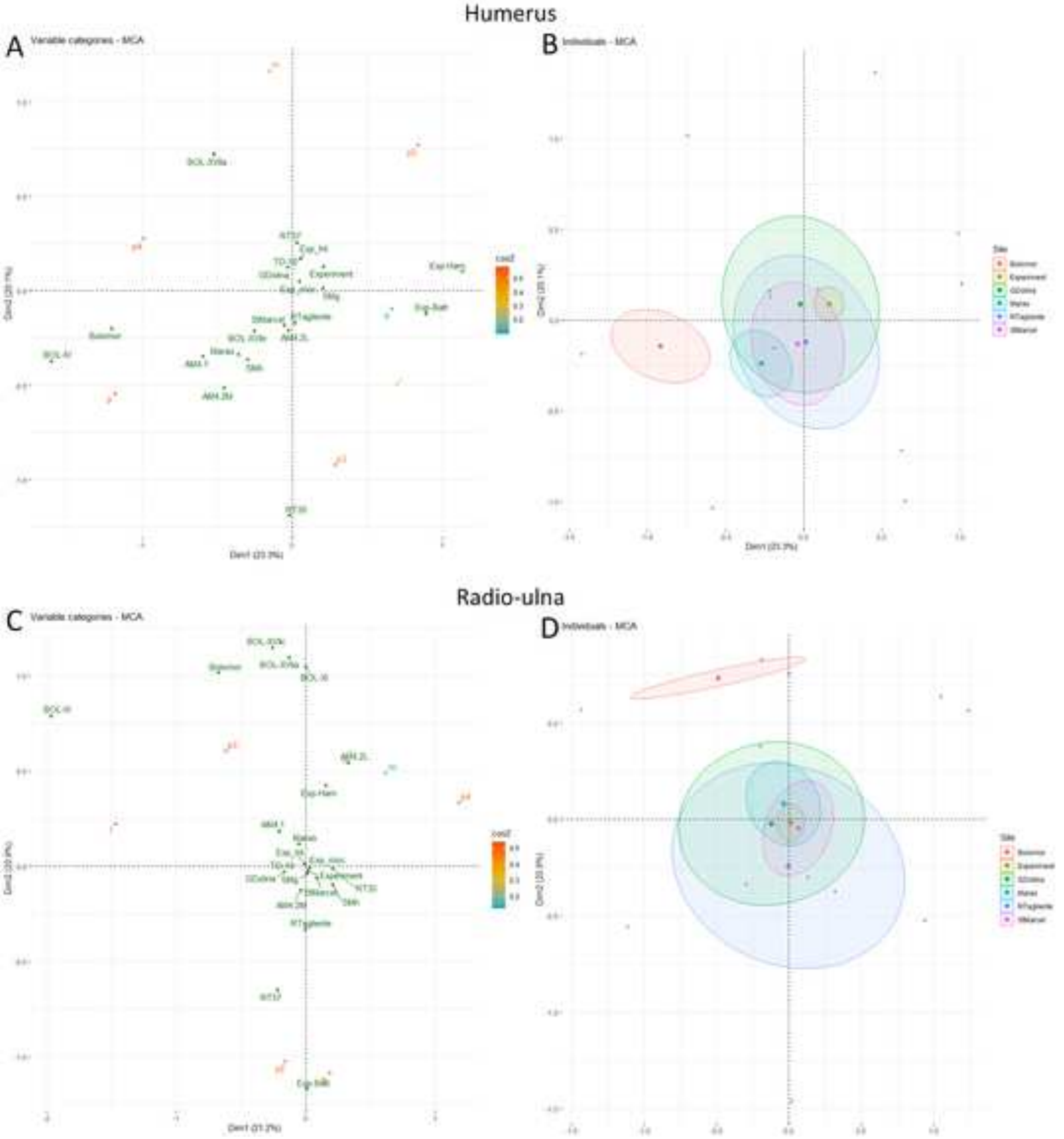

Figure 13

Click here to access/download;Figure;Figure_13_femur_tibia_mca_art_syn.tif

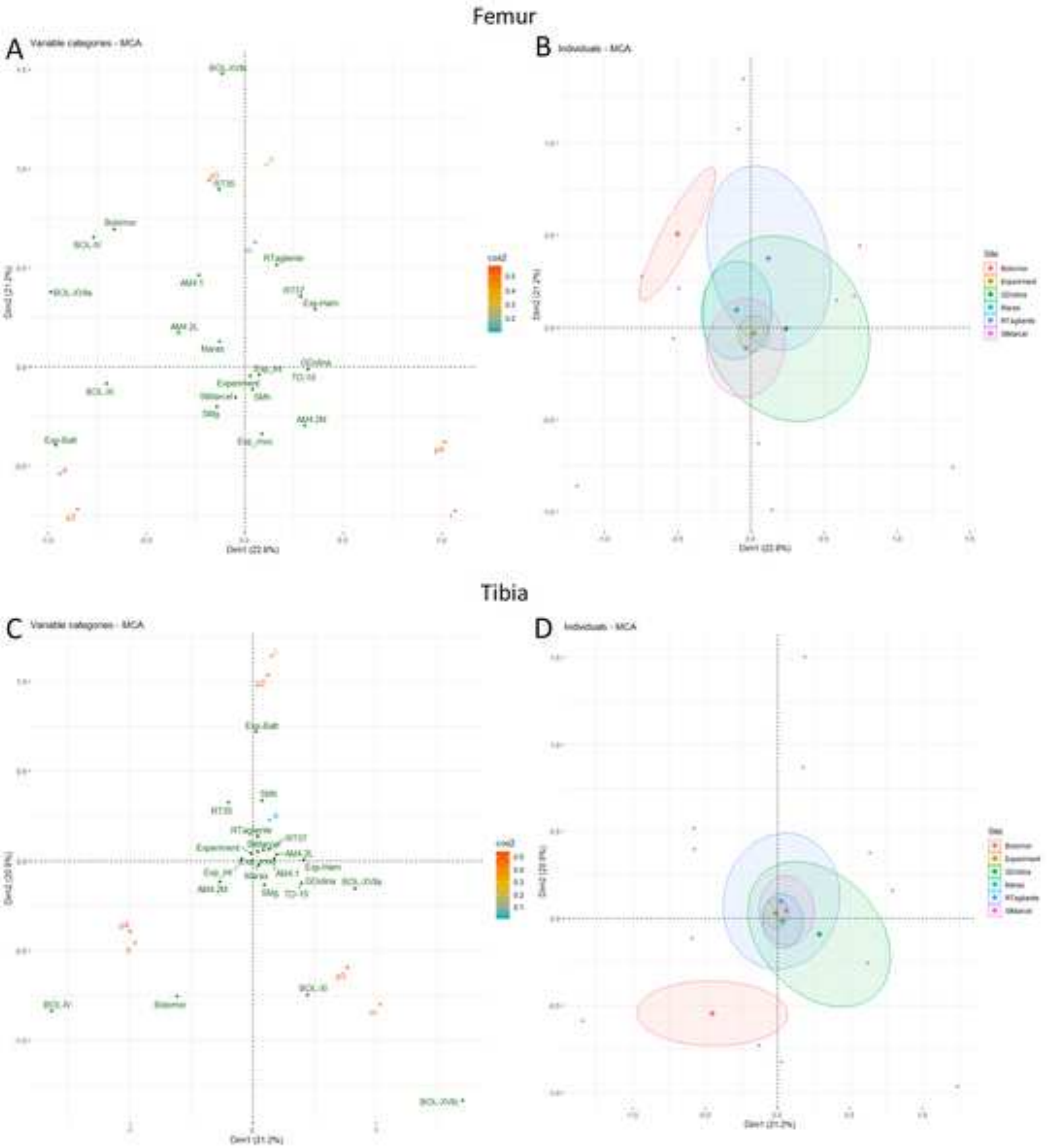

Figure 14

Click here to access/download;Figure;Figure_14_metacarpal_metatarsal_mca

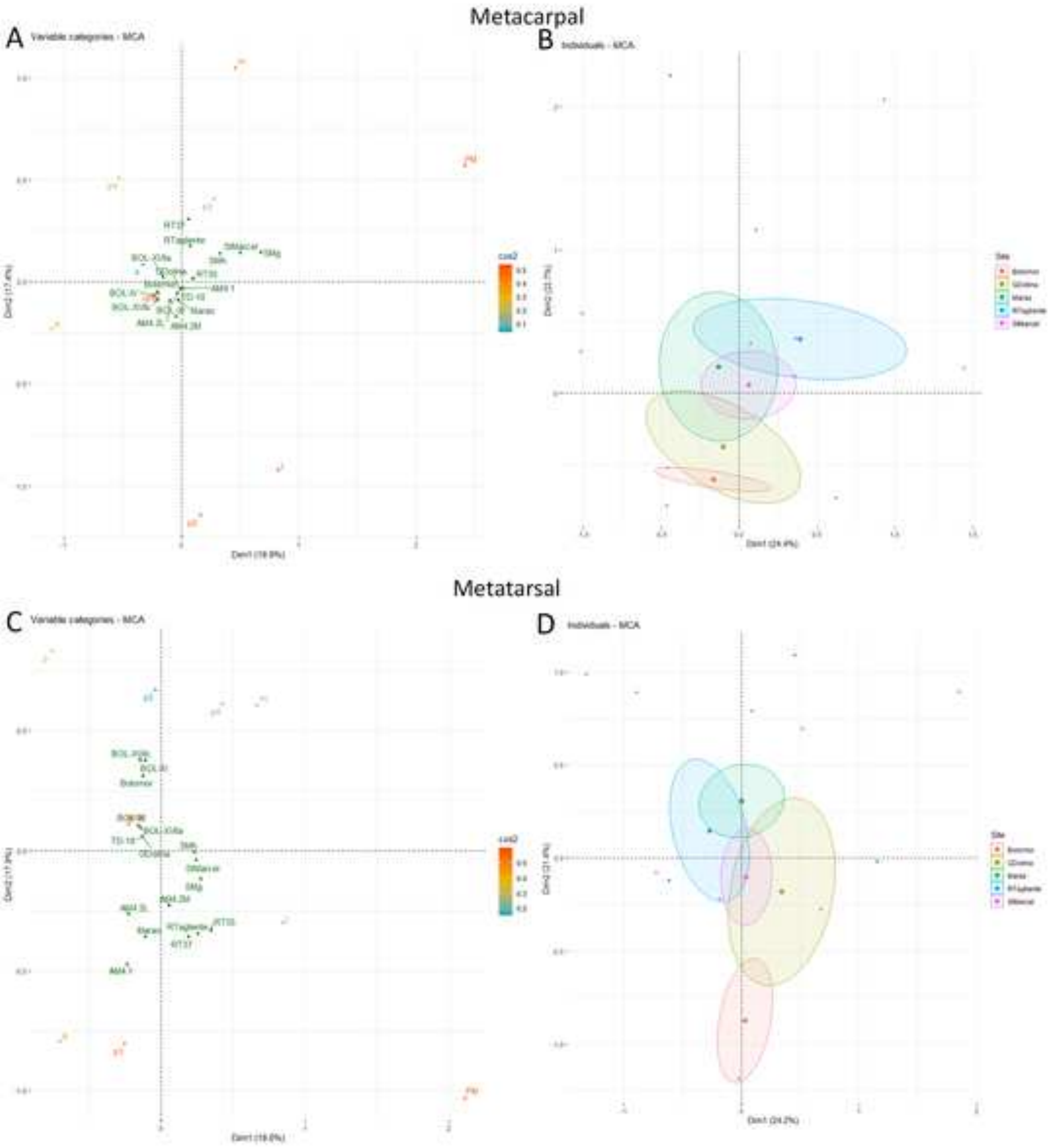

Click here to access/download
Supporting Information
Suporting information.docx
